# Structural Effects of Protein Aging: Terminal Marking by Deamidation in Human Triosephosphate Isomerase

**DOI:** 10.1371/journal.pone.0123379

**Published:** 2015-04-17

**Authors:** Ignacio de la Mora-de la Mora, Alfredo Torres-Larios, Sergio Enríquez-Flores, Sara-Teresa Méndez, Adriana Castillo-Villanueva, Saúl Gómez-Manzo, Gabriel López-Velázquez, Jaime Marcial-Quino, Angélica Torres-Arroyo, Itzhel García-Torres, Horacio Reyes-Vivas, Jesús Oria-Hernández

**Affiliations:** 1 Laboratorio de Bioquímica-Genética, Instituto Nacional de Pediatría, Secretaría de Salud, México, D.F., México; 2 Departamento de Bioquímica Y Biología Estructural, Instituto de Fisiología Celular, Universidad Nacional Autónoma de México, México, D.F., México; Centro Nacional de Biotecnologia - CSIC, SPAIN

## Abstract

Deamidation, the loss of the ammonium group of asparagine and glutamine to form aspartic and glutamic acid, is one of the most commonly occurring post-translational modifications in proteins. Since deamidation rates are encoded in the protein structure, it has been proposed that they can serve as molecular clocks for the timing of biological processes such as protein turnover, development and aging. Despite the importance of this process, there is a lack of detailed structural information explaining the effects of deamidation on the structure of proteins. Here, we studied the effects of deamidation on human triosephosphate isomerase (HsTIM), an enzyme for which deamidation of N15 and N71 has been long recognized as the signal for terminal marking of the protein. Deamidation was mimicked by site directed mutagenesis; thus, three mutants of HsTIM (N15D, N71D and N15D/N71D) were characterized. The results show that the N71D mutant resembles, structurally and functionally, the wild type enzyme. In contrast, the N15D mutant displays all the detrimental effects related to deamidation. The N15D/N71D mutant shows only minor additional effects when compared with the N15D mutation, supporting that deamidation of N71 induces negligible effects. The crystal structures show that, in contrast to the N71D mutant, where minimal alterations are observed, the N15D mutation forms new interactions that perturb the structure of loop 1 and loop 3, both critical components of the catalytic site and the interface of HsTIM. Based on a phylogenetic analysis of TIM sequences, we propose the conservation of this mechanism for mammalian TIMs.

## Introduction

Deamidation is the spontaneous loss of ammonium from the neutral amide side chains of asparagine and glutamine to produce the negatively charged carboxylate forms of aspartic and glutamic acid, respectively. The reaction occurs both *in vitro* and *in vivo*, with deamidation half-times that span from hours to years and depend on protein and milieu factors (For an extensive review on deamidation see [1]). The primary sequence and the three-dimensional structure of the protein are the main factors governing the deamidation rate; but the pH, the temperature and the ionic strength affect the rate of the process too. Deamidation introduces non-native negative charges into the protein sequence, and, in many cases, this modification induces conformational changes that affect the function, the structure and the stability of proteins [1, 2].

The disruptive effects of deamidation reactions provide no clear evolutionary advantage; even so, short-lived amides are preferentially accumulated in proteins with deamidation being the most commonly occurring post-translational modification [2]. Accordingly, it has been proposed that instability is the primary biological function of asparagine and glutamine, with both residues changing their properties with time, and serving as molecular clocks for the regulation of biological processes such as protein turnover, development and aging [2–8]. Until now, atomic-level structural information of deamidated proteins has been limited to structures where isoaspartate or succinimide (both intermediates in the deamidation reaction pathway) replace asparagine residues [9–14]. Only in a few cases, the physiological effects provoked by deamidation have been clearly related with specific structural changes [9, 13].

Triosephosphate isomerase (TIM) is a widely studied enzyme, both at a structural and functional level [15–19]. It was recognized early that human TIM (HsTIM) isolated from diverse tissues showed acidic isoforms, and that these isoforms accumulated with aging. Furthermore, it was demonstrated that these isoforms could be obtained *in vitro* by alkaline incubation of purified HsTIM [20]. Peptide fingerprinting analysis from both *in vivo* and *in vitro* samples indicated that acidic isoforms are the result of deamidation of two specific residues, N15 and N71. In the sequence of HsTIM, both asparagine residues are followed by glycine residues. It has been proven that the main factor related to deamidation propensity is the presence of asparagine-glycine pairs [7].

**Fig 1:**
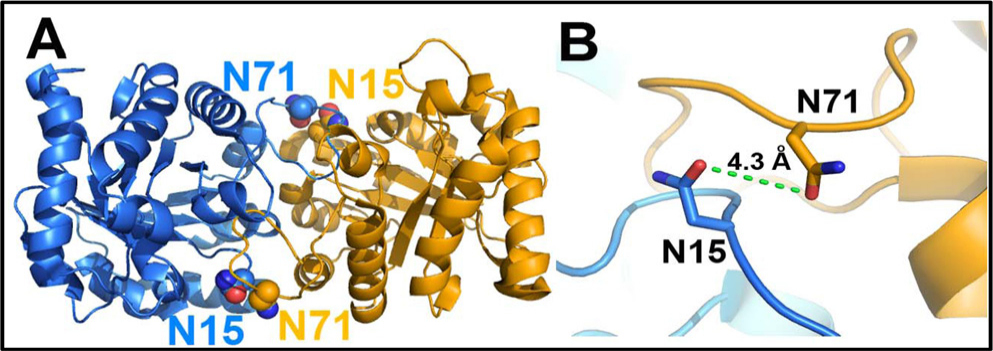
The two sites of deamidation of HsTIM are found close to each other. (A) Overall view of the WT HsTIM dimer (PDB code 2JK2) showing the position of N15 and N71; each subunit is depicted in blue and orange. (B) Close-up view of the two deamidating residues. N15 is found in loop 1 and N71 in loop 3.

It was also suggested that deamidation of HsTIM is sequential beginning at N71 and followed by N15. Even more, it was proposed that deamidation of N71 is a prerequisite for the deamidation of N15 [20]. Based on the crystal structure of the protein, which showed that N15 of one subunit is closely positioned to N71 of the adjacent subunit (Fig 1), it was suggested that the introduction of negative charges into the dimer interface could affect the stability of the enzyme by a mechanism of charge repulsion. In fact, it was shown that deamidated forms of HsTIM were more susceptible to dissociation [20].

Subsequently, it was demonstrated that the presence of substrate enhanced deamidation of HsTIM in a concentration-dependent manner, implicating that the catalytic events increased the probability of deamidation [21]. The substrate-induced deamidated enzyme was more susceptible to denaturing conditions and proteolytic digestion; therefore, it was proposed that HsTIM represents a case of “molecular wear and tear” for which catalysis promotes the terminal marking of the protein for degradation [22]. Additional experimental evidence suggests that deamidated HsTIM can be conjugated to Hsp73 or ubiquitin for its degradation [22]. Additional work with rabbit TIM confirmed the results obtained with HsTIM and supported the paradigm of terminal marking by deamidation of N15 and N71 in mammalian TIMs [23].

In this work, we deamidated HsTIM by changing N15 and N71 to aspartic acid, and demonstrated that the single deamidation of N15 is enough to trigger the disruptive structural and functional effects of deamidation. The crystal structure of the N15D mutant showed that the mutagenized residue adopted a new conformation that establishes alternate stable interactions with the protein, even at the expense of the loss of its original interactions and the disruption of the dimer assembly. Remarkably, this crystal structure provides atomic-level structural information about the mechanism by which deamidation can induce alterations of the structure and function of proteins. Finally, from the analysis of the amino acid sequence of TIMs from diverse phylogenetic groups, we propose that the terminal marking mechanism by deamidation of N15 is conserved in mammalian TIMs. Altogether, the results of this work improve the understanding of the proposed prevailing mechanism of terminal marking by deamidation of HsTIM and extend our knowledge about protein deamidation.

## Material and Methods

### Materials and general procedures

Analytical grade reagents, salts and buffers were acquired from Sigma-Aldrich; glycerol-3-phosphate dehydrogenase (GDH) was from Roche. Molecular biology reagents and enzymes were purchased from New England BioLabs and Invitrogen. Oligonucleotide synthesis and DNA sequencing was provided by the Unidad de Biología Molecular, Instituto de Fisiología Celular, UNAM. Crystallization plates and reagents were obtained from Hampton Research. Protein concentration was determined by bicinchoninic acid assay, or by absorbance at 280 nm considering ε_280_ = 32,595 M^-1^ cm^-1^ for pure HsTIM. SDS-PAGE electrophoresis was performed according to Schägger and von Jagow [24], native electrophoresis was carried out with Tris-Glycine pH 8.5 buffer [25], staining was performed with Coomassie brilliant blue G. The activity of TIM was measured spectrophotometrically at 25°C in the direction of D-glyceraldehyde 3-phosphate (GAP) to dihydroxyacetone phosphate (DHAP) by following the oxidation of NADH at 340 nm in a coupled assay [26]. The standard reaction mixture contained 100 mM triethanolamine/10 mM EDTA pH 7.4 (TE buffer), 1 mM GAP, 0.2 mM NADH and 1 unit of GDH; the reaction was initiated by the addition of 5 ng/mL of WT or N71D HsTIM, 60 ng/mL of N15D or 80 ng/mL of N15D/ N71D. For the WT and the N71D mutant, *V*_*max*_ and K_m_ were obtained by fitting initial velocity data (GAP concentrations from 0.02 to 4 mM), to the Michaelis-Menten equation (*vi* = *V*_*max*_[S]/K_m_+[S]) by non-linear regression calculations. For the N15D or the N15D/N71D mutants no saturation was observed at the highest concentrations of substrate that could be assayed; therefore, data could not be fitted and *V*_*max*_ and K_m_ values were not calculated. For the WT and the N71D mutant, *k*_*cat*_ values were derived from *V*_*max*_ by considering a monomer molecular mass of 26.8 kDa. For the N15D and the N15D/N71D mutants, *k*_*cat*_/K_m_ ratios were approximated from the slope of double reciprocal plots according to *k*_*cat*_/K_m_ = 1/*m* • [enzyme] (where *m* represents K_m_/*V*_*max*_) and by using the stated molecular mass. Turnover induced deamidation was performed essentially as reported by Yüksel and Gracy [21]; briefly, enzyme (1 mg/mL) was incubated in TE buffer at 37°C with 3.5 mM GAP in a temporal course of 5 hours and analyzed by native gel electrophoresis.

### Construction, expression and purification of HsTIM WT and mutants

The WT HsTIM gene [27] was subcloned into the pET-HisTEVP plasmid [28] after digestion with *NdeI* and *BamHI*. The pET-HisTEVP vector introduces an amino-terminal (His)_6_-Tag sequence, which is removable by the tobacco etch virus protease (TEVP). Using this template, the single N15D and N71D mutants were constructed by site-directed mutagenesis. Mutagenic oligonucleotides were Fw 5’-GAAGATGGATGGGCGGAA-3’ and Rv 5′-TTCCGCCCATCCATCTTC-3′ for N15D, and Fw 5′-CAAAGTGACTGATGGGGCT-3’ and Rv 5′-AGCCCCATCAGTCACTTTG-3′ for N71D. In both cases, external T7 promoter and T7 terminator oligonucleotides were used. PCR conditions were as follows, 94°C for 4 min, 25 cycles of 1 min at 94°C, 1 min at 53°C, 1 min at 72°C and a final extension step of 10 min at 72°C. The double mutant N15D/N71D was prepared from single mutant constructions taking advantage of a unique *SacI* site located between the two mutations (at 74 bp from the ATG codon). Both constructions were restricted with *SacI* and *BamHI* and from pET-HisTEVP-N15D, a fragment of 4730 bp comprising the plasmid and the first 74 bp of the gene was purified; a fragment of 670 bp fragment between *SacI* and *BamHI* was obtained from pET-HisTEVP-N71D. The ligation reaction of both fragments renders the complete HsTIM gene, containing both the N15D and the N71D mutations in the pET-HisTEVP vector. The successful construction of all single and double mutants was confirmed by automated DNA sequencing of the complete genes.

Expression was performed in the *E*. *coli* BL21-CodonPlus (DE3)-RIL strain (Stratagene). As previously shown, HsTIM is expressed as two isoforms by misincorporation of lysine for arginine in BL21(DE3)pLys cells [29]; therefore, to avoid sample heterogeneity, the CodonPlus strain containing extra copies of the genes that encode for arginine, isoleucine and leucine tRNAs was used to produce the recombinant proteins. Transformed cells were grown at 37°C until an ΔOD_600nm_ of 0.8 was reached; protein expression was induced with 0.4 mM IPTG and continued for 3 hours at 30°C. Purification of recombinant HsTIM (WT and mutants) was performed using immobilized-metal affinity chromatography as reported for *Giardia lamblia* TIM with minor modifications [28]. Briefly, cells were harvested by centrifugation and broken by sonication. The resultant suspension was centrifuged and the supernatant applied to a Profinity IMAC Resin column (Biorad); after washing, protein was eluted with 250 mM imidazole. The (His)_6_-tag was removed by overnight incubation with His-tagged TEVP. An additional IMAC step was performed to remove TEVP and non-cleaved HsTIM. All purified proteins were concentrated and dialyzed against TE buffer for immediate use, or precipitated in the presence of 75% ammonium sulfate and refrigerated at 4°C for storage. Recombinant His-tagged TEVP was obtained as previously described [27].

### Spectroscopic characterization

Secondary structure was analyzed by circular dichroism in a Jasco J-810 spectropolarimeter equipped with a thermostated Peltier-controlled cell holder. Spectral scans at 25°C were performed from 190 to 260 nm at 0.1nm intervals in a quartz cell with a path length of 0.1 cm. The assays were conducted with 100 μg/mL of protein in 25 mM phosphate buffer, pH 7.4. Tertiary structure was evaluated by protein intrinsic fluorescence in a Perkin-Elmer LS-55 fluorescence spectrometer. The fluorescence emission spectra from 310 to 500 nm were recorded after excitation at 280 nm with excitation and emission slits of 10 and 7.5 nm, respectively. Assays were performed at 25°C in phosphate buffer with 100 μg/mL of protein. For all spectroscopic assays, blanks without protein were subtracted from the experimental samples.

### Evaluation of protein stability

For the evaluation of thermal stability, protein unfolding was followed as the change in the circular dichroism signal at 222 nm in temperature scans ranging from 20 to 90°C in increases of 1°C/2.5 min. The fraction of unfolded protein and the melting temperature (T_m_) values were calculated as reported [30]. Limited proteolysis assays were performed essentially as described [31]. In brief, HsTIM (1mg/mL) and proteinase K from *Tritirachium album* (Sigma-Aldrich) were incubated at 30°C at a molar ratio of 265:1 (TIM:Proteinase K). At the times indicated, reactions were arrested by adding 5 mM phenylmethylsulphonyl fluoride (PMSF); thereafter, aliquots were withdrawn for electrophoresis and residual activity determination. Structural compactness was evaluated by the accessibility of cysteine residues to Ellman's reagent (5,5'-dithiobis-(2-nitrobenzoic acid); DTNB). The time-course of chemical modification of HsTIM (300 μg/mL) by DTNB (1mM) was evaluated by following the absorbance signal at 412 nm in a Varian Cary 50 spectrophotometer [32]. The stability of the HsTIM dimer was assayed by size-exclusion chromatography; protein samples at a concentration of 500 μg/mL were incubated for 3 hours at 30°C and loaded onto a Superdex S-75 column coupled to an Akta FPLC system (Amersham Biosciences‎). The column had been previously equilibrated with 50 mM Tris-HCl pH 8.0, 150 mM NaCl, 5% (v/v) glycerol and calibrated with molecular mass standards.

### Crystallization, data collection, structure determination and refinement

Crystallization assays were performed with the sitting drop vapor diffusion method. For all proteins, one microliter of reservoir solution was mixed with 1 μL of protein solution (N15D 22 mg/mL, N71D 29.5 mg/mL). Crystals grew at 18°C in the following conditions from Hampton Research screens: N15D, 0.1 M Tris pH 8.5, 0.2 M ammonium acetate, 25% w/v polyethylene glycol 3350 (Index screen, condition G9). N71D: 0.1 M Tris pH 8.5, 0.01 M nickel chloride, 20% w/v Polyethylene glycol monomethyl ether 2000 (Crystal Screen, condition H9). Prior to freezing in liquid nitrogen, crystals were cryoprotected by increasing the corresponding concentrations of precipitant to 35% (v/v). Diffraction data were collected at the Life Sciences Collaborative Access Team (LS-CAT), beam-lines 21-ID-F and 21-ID-G, at the Advanced Photon Source (Argonne National Laboratory), using a MarCCD detector. Data were indexed, integrated and scaled with Scala [33] and XDS [34]; diffraction phases were determined by molecular replacement with PHASER [35], using the HsTIM coordinates (PDB code 2JK2) [36] as the starting model. Refinement was made with REFMAC [37] and the model was built with COOT [38]. Five per cent of the data were used to validate the refinement. σA-weighted 2Fo-Fc and Fo-Fc simulated annealing omit maps were used to further validate the quality of the model maps. Figures were prepared with PyMOL (http://www.pymol.org). Data collection and refinement statistics are given in Table 1. The atomic coordinates and structure factors have been deposited in the Protein Data Bank with the accession numbers 4UNK for the N15D and 4UNL for the N71D mutants.

**Table 1:** Data collection, refinement statistics and quality of crystallographic structures.

DATA COLLECTION STATISTICS		
PARAMETERS	HsTIM N15D	HsTIM N71D
Space group	P212121	P212121
Monomers per asymetric unit	2	2
Unit cell: a,b,c (Å)	47.6,70.7,146.4	64.9,72.8,93.3
	90,90,90	90,90,90
Resolution range (Å)	45.29–2.00	39.28–1.50
Unique reflections	34336	67516
Average multiplicity	4.7	3.7(3.6)
Completeness (%)	99.41(100)	96.6(99.1)
*I/σ(I)*	10.8	7.2
*Mean (I/sd(I))*	11.3 (4.0)	10.9(2.3)
R_merge_ (%)	8.8(35.8)	6.4(50.7)
R_work_/R_free_ (%)	17.9/22.4	19.8/22.5
Water molecules per asymmetric unit	452	350
RMSD from ideal: bond lengths (Å)	0.007	0.006
RMSD from ideal: bond angles (°)	1.024	1.025
Mean overall B value (Å^2^)	30.37	18.5
Ramachandran plot (%):		
Preferred regions	96.07	96.69
Allowed regions	3.93	2.90
Outliers	0.00	0.41
PDB code	4UNK	4UNL

Values in parentheses are for the last resolution shell.

### Phylogenetic analysis

Phylogenetic analysis of TIM was performed over 221 sequences retrieved from the Reference Sequence collection (RefSeq) at the National Center for Biotechnology Information (NCBI) [39]. Progressive multiple sequence alignment was calculated with the Clustal_X package [40], using the Gonnet 250 matrix [41]. Phylogenetic analysis was performed with Mega 5.21 software [42] using the Maximum Likelihood method under the Jones–Thornton–Taylor (JTT) substitution model for amino acid sequences.

## Results

### HsTIM mutants were efficiently produced without major alterations on their structure

Deamidations were introduced into HsTIM by site directed mutagenesis generating single (N15D and N71D) and double (N15D/N71D) mutants. Full gene sequencing corroborated the specific substitution of asparagine by aspartic acid at the desired positions (data not shown). The WT and mutant proteins were easily produced; protein expression in the *E*. *coli* CodonPlus strain plus purification with the (His)_6_-Tag and the TEV cleavage system yielded 20 to 25 mg of pure-homogenous recombinant protein per liter of bacterial cell culture. Mutants were distinguishable in native gel electrophoresis due to the gain of one negative charge per monomer in HsTIM N15D and N71D, or two negative charges in the double mutant N15D/N71D (S1 Fig). The spectroscopic characterization of WT HsTIM and the mutants indicates that deamidation induces minor changes in the secondary and tertiary structure, as indicated by the similarity of the far-UV circular dichroism and intrinsic fluorescence spectra (S2 Fig). Altogether, the results indicate that in the single or double deamidated forms of HsTIM, the global structure of the protein is preserved.

### Deamidation of N15 is the main contributor to the perturbation of the catalytic properties of HsTIM

**Fig 2:**
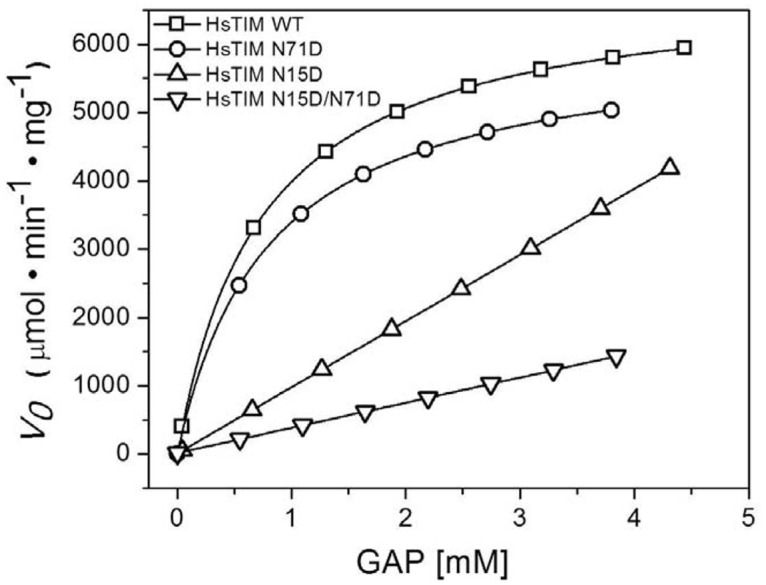
The N15D mutation strongly affects the activity of HsTIM. Initial velocity data were collected in reaction media containing 100 mM triethanolamine/10 mM EDTA pH 7.4, 0.2 mM NADH, 5 unit of GDH and the GAP concentrations indicated in the abscissa. The reaction was initiated by addition of 5 ng/mL of WT or N71D HsTIM, 60 ng/mL of N15D or 80 ng/mL of N15D/ N71D. For the WT and N71D mutant, lines represent the fit to the Michaelis-Menten equation. The data could not to be fitted for the N15D and N15D/N71D mutants; hence, kinetic constants were not obtained. The experiment is representative of independent triplicate assays; the difference in the calculated kinetic constants was less than 5%.

In order to characterize the effect of deamidation on the catalytic properties of HsTIM, the enzyme kinetics of the WT and mutants was explored (Fig 2). The N71D mutation induces minor changes in HsTIM; the WT and N71D enzymes obeys Michaelis-Menten kinetics with *V*_*max*_ values of 6920 and 6090 μmol min^-1^ mg^-1^, respectively, and practically identical K_m_ values (0.74 *vs* 0.79 mM for the WT and the N71D mutant). In contrast, the N15D mutation significantly affects the binding of substrate and catalysis of the enzyme. The most notorious effect of deamidation of N15 can be related to a decreased affinity of the enzyme, since saturation was not observed at the highest concentrations of substrate that could be assayed (Fig 2). The effect of the N15D mutation on the catalytic properties of HsTIM was synergistic with the N71D mutation; the double mutant N15D/N71D showed an additional reduction on the velocity of the enzyme (Fig 2). As the kinetic data for the N15D and the N15D/N71D mutants could not be fitted, *k*_*cat*_/K_m_ ratios for these enzymes were approximated from the slope of linear fits of double reciprocal plots and used for comparison with the WT and the N71D mutant (Table 2). The kinetic characterization of the three mutants indicates a differential effect of the deamidations for HsTIM, with the deamidation of N15 having a preponderant role in the impairment of the catalytic properties of the enzyme.

**Table 2:** Kinetic constants for the WT and HsTIM mutants.

Protein	*k*_*cat*_	*k*_*cat*_/K_m_^MM^	*k*_*cat*_/K_m_^LB^
	(10^5^ min^-1^)	(10^5^ min^-1^ mM^-1^)	(10^5^ min^-1^ mM^-1^)
WT	1.85	2.55	2.50
N71D	1.63	2.10	2.14
N15D	—	—	0.25
N15D/N71D	—	—	0.01

For the WT and the N71D mutant, *k*_*cat*_ and *k*_*cat*_/K_m_^MM^ were derived from *V*_*max*_ and K_m_ values calculated from the non-linear fit of initial velocity data to the Michaelis-Menten equation. For the N15D and the N15D/N71D mutants, *k*_*cat*_/K_m_^LB^ ratios were approximated from the slope of Lineweaver-Burke plots as indicated in the Material and Methods section; for comparison, the data for the WT and the N71D mutant were processed in the same way.

### Deamidation of N15, but not of N71, perturbs the stability of HsTIM

**Fig 3:**
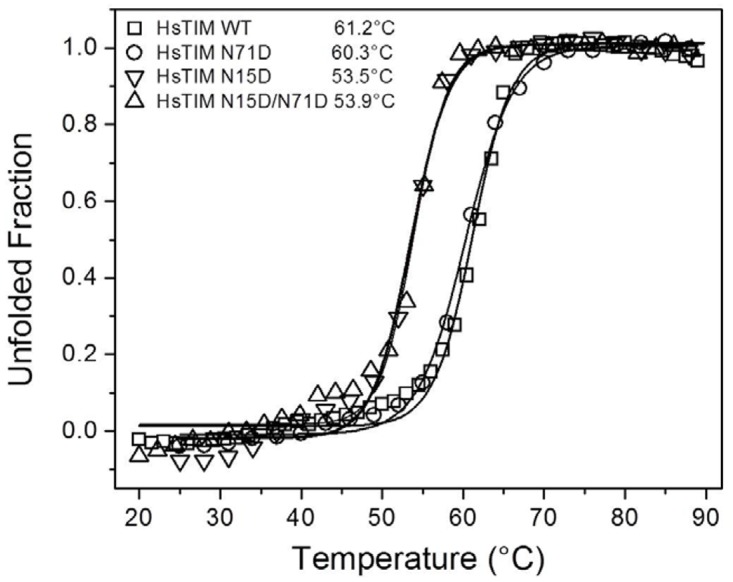
The N15D mutation, but not the N71D mutation, decreases the thermal stability of HsTIM. The thermal unfolding of 0.1 mg/mL of WT and deamidated TIMs was monitored by recording the change in the CD signal at 222 nm in response to heating. The temperature was increased from 20 to 90°C at 1°C/2.5 min. The fraction of unfolded protein and the T_m_ values (inset) were calculated as previously described (24). Experiments were performed in duplicate; standard errors were less than 5%.

The destabilization of HsTIM is a major characteristic of terminal marking by deamidation; therefore, protein stability was thoroughly studied in WT HsTIM and the mutated proteins. We initially performed thermal induced denaturation assays, recording the change in the circular dichroism signal at 222 nm as function of temperature (Fig 3). The results showed that the T_m_ for the N71D mutant closely resembles the T_m_ of the WT enzyme (61.2 *vs* 60.3°C for the WT and the N71D HsTIM, respectively); in contrast, the T_m_ decreased at least 7°C with respect to the WT enzyme for the N15D or the N15D/N71D mutants (53.5 and 53.9°C for the N15D and N15D/N71D mutants, respectively). As can be observed, the single deamidation of N15 is sufficient to disrupt the thermal stability of HsTIM.

**Fig 4:**
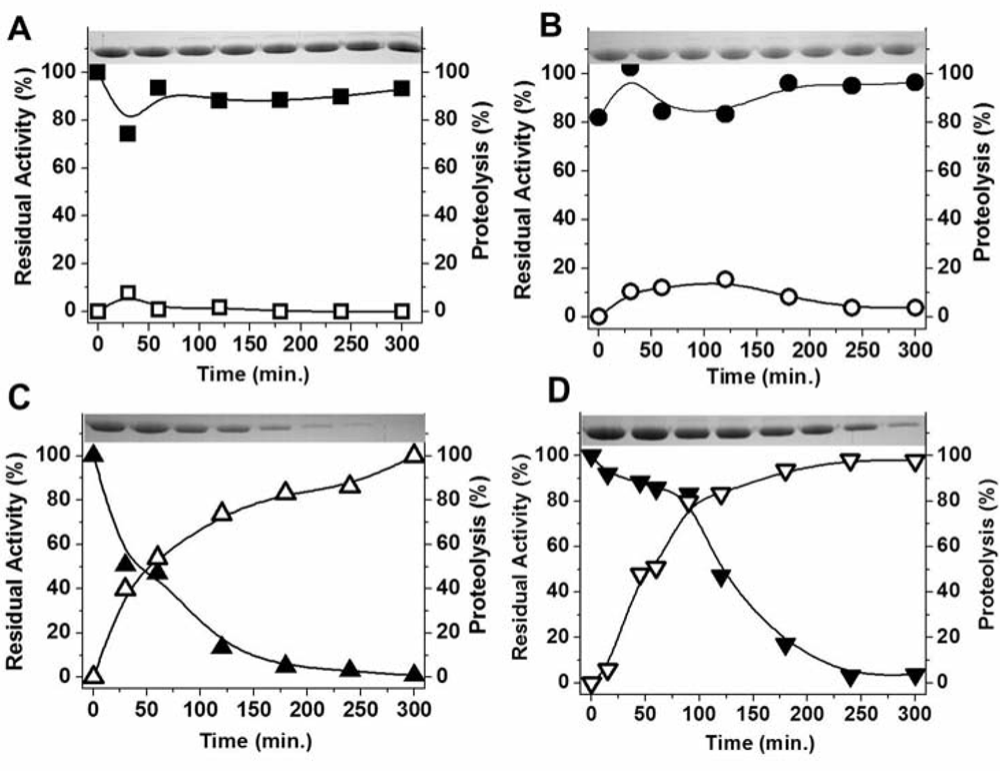
The N15D mutation increases the susceptibility of HsTIM to proteolysis. The proteolysis of WT (A), N71D (B), N15D (C) and N71D/N15D (D) with Proteinase K was followed by SDS-PAGE (upper insets, open symbols) or residual activity (closed symbols). WT or mutant TIMs (1mg/mL) were incubated with Proteinase K at 30°C at a molar ratio of 265:1 (TIM:Proteinase K). At the times indicated in the ordinate axis, the reaction was arrested by the addition of PMSF 5 mM and aliquots withdrawn for analysis. SDS-PAGE was digitalized and analyzed densitometrically; TIM activity was measured under standard conditions. The experiment is representative of a triplicate; standard errors were less than 10%.

Increased susceptibility to proteolysis induced by deamidation was described as a prominent feature of the terminal marking mechanism in HsTIM [22]; we therefore studied the proteolytic digestion with proteinase K of the WT and the deamidated HsTIM mutants (Fig 4). Proteinase K is a broad-spectrum serine protease previously used to characterize stability perturbations of HsTIM [32]. The results showed an increased susceptibility of the N15D and N15D/N71D mutants to proteolysis. Under very soft proteolysis conditions, where the WT and N71D proteins are completely insensitive to proteinase K (Fig 4, panels A-B), the N15D and N15D/N71D mutants were completely hydrolyzed (Fig 4, panels C-D). The results indicate that deamidation of N71 does not contribute to the increased susceptibility of HsTIM to proteolysis.

**Fig 5:**
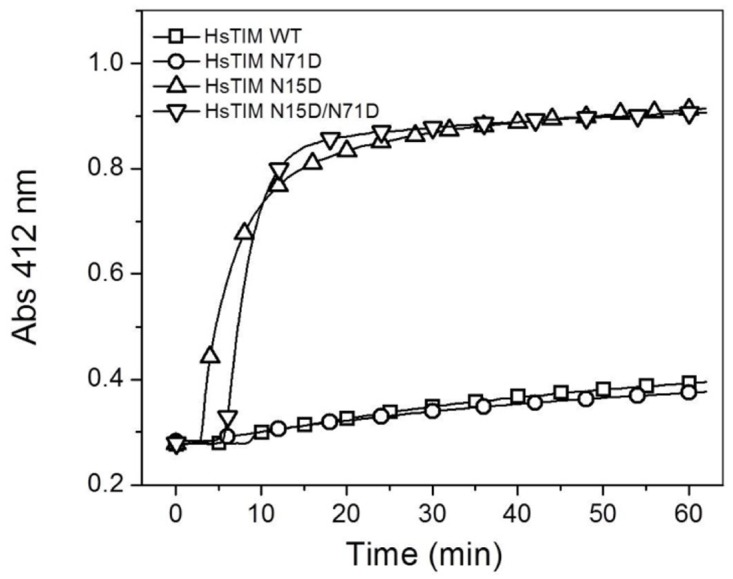
The N15D mutation decreases the structural compactness of HsTIM. The derivatization of cysteine residues in TIM (300 μg/mL) by DTNB (1 mM) was followed spectrophotometrically at 412 nm. The experiment is representative of duplicate experiments qualitatively identical.

The susceptibility of the N15D and N15D/N71D mutants to proteolysis suggests that these proteins have a more relaxed 3D structure [32]; therefore, the compactness of WT HsTIM and the mutants was tested by the accessibility of their cysteine residues to Ellman´s reagent (DTNB) (Fig 5). Each WT HsTIM monomer has five cysteine residues, which are totally buried (less than 1% of accessible surface area for each of the ten cysteines in the dimer of the 2JK2 structure; data not shown), distributed throughout the structure of the protein. Therefore, the velocity of chemical modification (derivatization) of the cysteine residues by DTNB can be considered as an index of the structural compactness in the protein [32]. The results showed that the WT or the N71D HsTIM are derivatized slowly in comparison with the N15D and the N15D/N71D mutants (Fig 5). Under identical experimental conditions, DTNB derivatizes only 0.7 cysteines per monomer in the WT or the N71D enzymes after one hour; in contrast, in the N15D or N15D/N71D mutants 4.6 cysteines per monomer are derivatized in the first 30 minutes of the experiment (Fig 5). The result supports the idea of a loosening of the three-dimensional structure of the N15D and N15D/N71D mutants.

**Fig 6:**
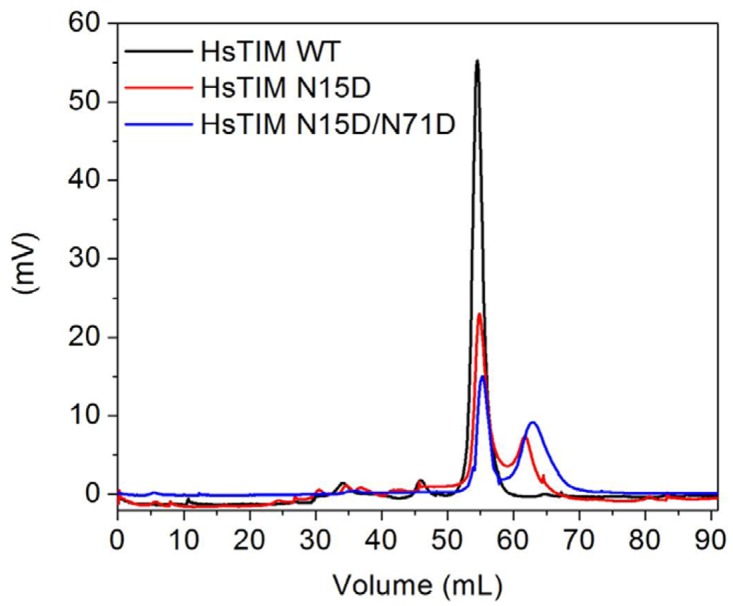
The N15D mutation favors the dissociation of HsTIM. The stability of HsTIM dimers was tested by incubating enzymes (500 μg/mL) at 30°C for 3 hours and then loading them onto a size-exclusion chromatography column (Superdex 75) equilibrated and developed in 50 mM Tris pH 8.0 plus 150 mM NaCl and 5% (v/v) glycerol. For simplicity, only the data for the WT, the N15D and the N15D/N71D proteins are shown. The profile for the N71D mutant overlaps with that of the WT enzyme.

The interfacial charge-repulsion assumption explaining HsTIM destabilization arose from the close position of N15 and N71 in adjacent subunits [20] (Fig 1). To test the contribution of deamidation to dimer stability, we performed size-exclusion chromatography of WT HsTIM and the mutants after incubation at 30°C for 3 hours (Fig 6). Whereas the WT and the N71D mutants elute as single peaks with retention volumes corresponding to native dimers (55 mL, 53 kDa), the N15D and N15D/N71D mutants show two well defined peaks. The first peak corresponds to native dimers, whereas the second closely corresponds to monomers (62 mL and 30 kDa for the N15D mutant and 63 mL and 31 kDa for the double mutant). The results indicate that the N15D and the N15D/N71D mutants are prone to monomerization.

### The deamidation rate of N15 does not depend on deamidation of N71

**Fig 7:**
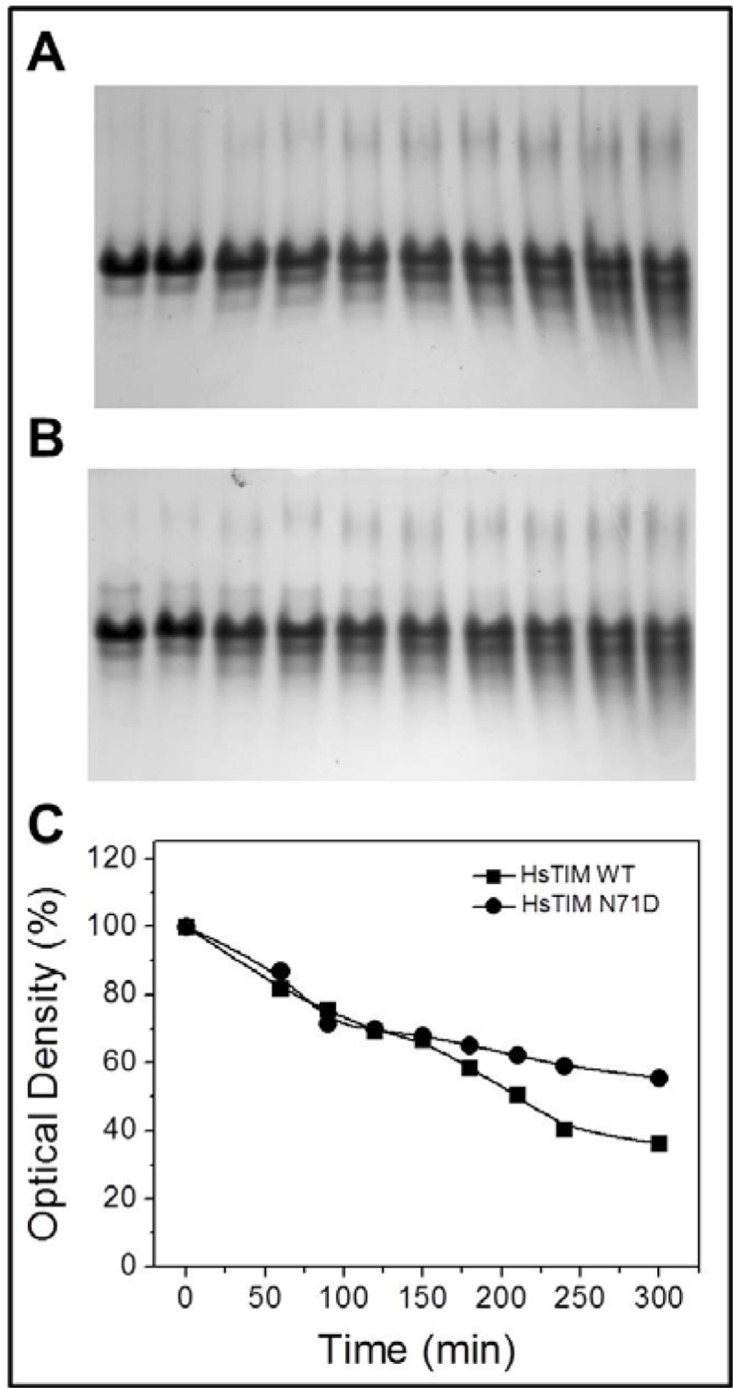
The deamidation of N15 does not depend on deamidation of N71. Native gel electrophoresis of the WT (A) or the N71D mutant (B) after incubation at 1mg/mL in TE buffer at 37°C with 3.5 mM GAP in a temporal course of 5 hours. The appearance of additional bands with higher electrophoretic mobility indicates the emergence of negatively charged additional species. The deamidation rate in the WT and the N71D mutant (C) was determined as the percentage decrease of the upper band (native enzyme). The experiments are representative of independent, duplicate assays with differences of less than 10%.

The prevailing terminal marking mechanism proposed for HsTIM establishes that deamidation of N71 is a prerequisite for the deamidation of N15 [20–22]. In order to test the influence of N71 deamidation on the deamidation of N15, the deamidation rate, as measured by the decrease of native enzyme with the concomitant appearance of negatively charged bands in a temporal course followed by native gel electrophoresis, was tested in the WT and in the N71D mutant (Fig 7). It was supposed that if deamidation of N71 favors the deamidation of N15, the N71D mutant (where deamidation already had occurred), must be deamidated faster than the WT enzyme. As can be observed (Fig 7), this is not the case, since both proteins are deamidated at very similar rates. This result indicates that deamidation of N71 does not influence the deamidation rate of N15.

### The crystal structures of the HsTIM mutants explain the structural and functional effects of deamidation

In order to ascertain how deamidations affect the three-dimensional structure of HsTIM, we solved the crystal structure for the N15D and N71D mutants. Both proteins yielded orthorhombic crystals with space group P212121 displaying one dimer per asymmetric unit. Crystals diffracted to resolutions of 2.0 and 1.5 Å for the N15D and N71D mutants, respectively, and produced good quality data as indicated by the collection and refinement statics (Table 1) and simulated annealed omit electron density maps (S3 Fig).

**Fig 8:**
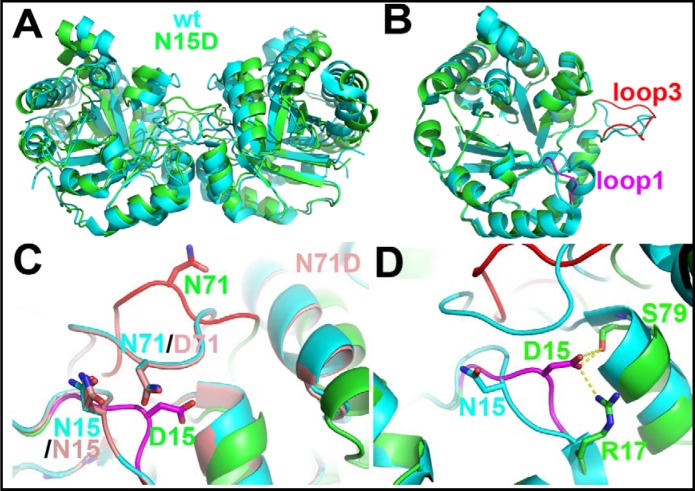
The N15D mutant is solely responsible for the structural changes induced by deamidation in HsTIM. (A) The enzyme dimers of the wild type and the N15D mutant do not superpose. Superposition of the dimers of WT HsTIM (cyan) and the N15D mutant (green). The monomers are closer to each other in wild-type TIM when compared to the N15D mutant enzyme. If this superposition is made using a monomer as a reference, the angle of association is changed by 14°, as defined by the DynDom server (http://fizz.cmp.uea.ac.uk/dyndom/). (B) The monomeric subunits superpose well. If only the protein monomers are superimposed, the overall RMSD value on Cα is 0.6 Å (see S5 Fig). There are two main regions that change their overall conformation, loop 1 (in magenta, RMSD of 3 Å) and loop 3 (in red, RMSD of 4 Å). (C) The N71D mutation has no structural effects on the enzyme. A closer look on the region of loop 1 and loop 3 shows that, whereas the N15D mutation (in magenta and red) undergoes a conformational change in both regions, the N71D mutation (in light red) keeps the structure of the WT HsTIM. (D) Residue 15 on the mutant N15D makes two new interactions. A novel intersubunit interaction is made between the side chains of D15 and S79. Also, an intrasubunit interaction is made with the side chain of R17. In the wild-type enzyme, R17 makes intersubunit interactions with the side chains of T70 and N71 (see Fig 9).

**Fig 9:**
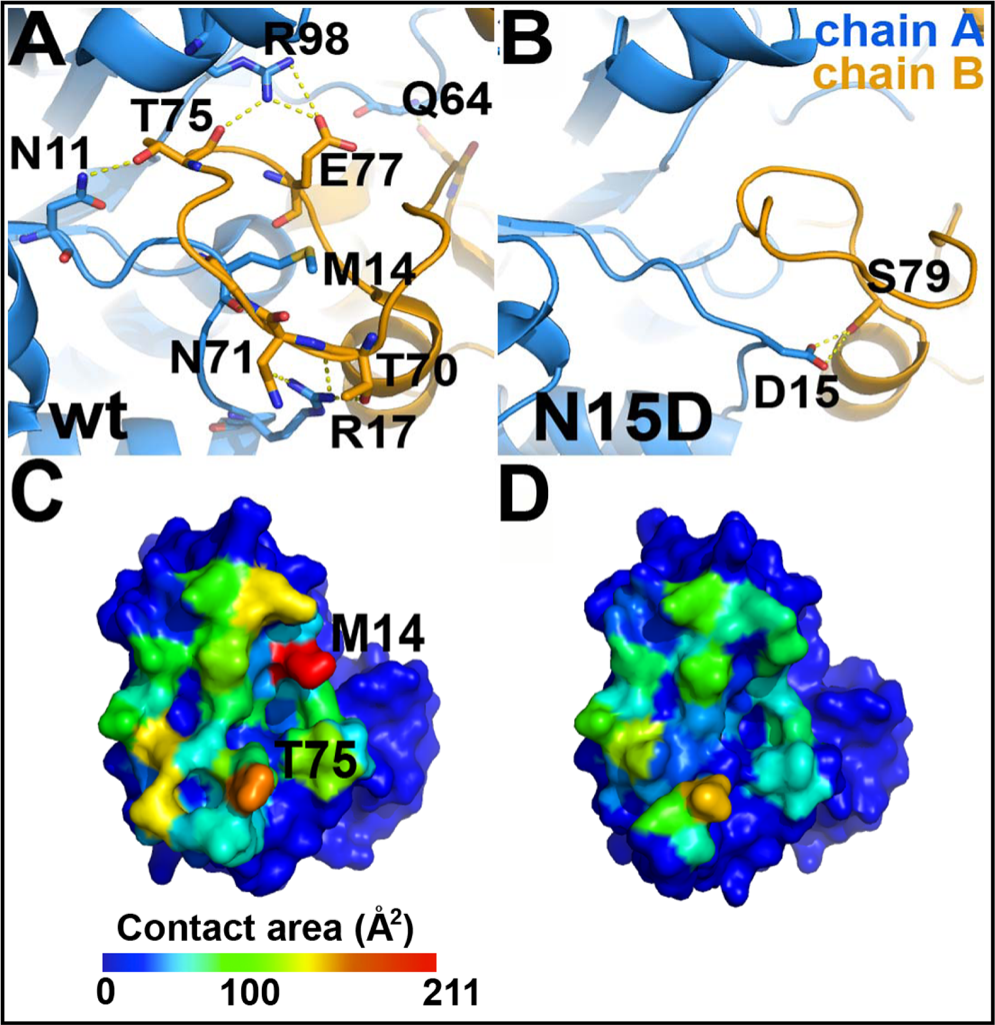
The dimeric interface is highly perturbed in the structure of the N15D mutant. Panels A and C correspond to the WT HsTIM, whereas B and D correspond to the mutant N15D. (A) Hydrogen bonds in the interface of the wild-type enzyme that are lost in the N15D mutant (see S1 Table). The interactions were calculated with the HBplot server (http://dept.phy.bme.hu/virtuadrug/hbplot/bin/infopage.php). (B) There is a new intersubunit hydrogen bond in the N15D mutant that is not present in the wild type enzyme. This interaction is made between the mutated residue 15D and the side chain of S79. (C and D) Surface of a monomer interacting with the neighboring protein subunit. The total contact area per residue decreases from 2486 Å^2^ in the wild type enzyme (C) to 1561 Å^2^ on the N15D mutant (D) (see S2 Table), with the greatest decrease represented by residue M14 (red in C). These panels were drawn according to the results of the Contact Map Analysis server (http://ligin.weizmann.ac.il/cma/), which were plotted on the monomer surface according to the contact area of each residue on the interface on a blue (0 Å^2^) to red (211.8 Å^2^ for M14) scale basis.

The comparison between the crystal structures of the WT HsTIM (PDB code 2JK2) and the N71D mutant shows that both structures are highly similar (S4A Fig, Fig 8C), with a Cα RMSD of 0.26 Å; the lateral chain of D71 is in the same conformation as the parental N71 (S4B Fig, Fig 8C). There are minimal discernible changes in the region of the mutation; in the B subunit, the N15 sidechain is now found in a double conformation (S4C Fig, Fig 8C). One conformation shows the same position as that in the WT structure, whereas in the alternate conformation the sidechain moves slightly towards the mutated D71 residue (S4B Fig, Fig 8C). This local change does not alter the structure in the vicinity of the mutation (S4B Fig, Fig 8C), nor does it affect the geometry of the active site (S4D Fig). The results are consistent with the mild effects of the N71D mutation on the structure and function of HsTIM. In contrast, the crystal structure of the N15D mutant provides a clear atomic description of the conformational changes explaining the detrimental effects of deamidation on HsTIM. The superposition of the WT and N15D structures shows that deamidation of N15 disturbs the assembly of the dimer (Fig 8A); the association between the monomers is changed in such a way that the angle between both subunits in the N15D structure is increased by 14°, with reference to the WT structure (Fig 8A). A close examination of the WT and N15D HsTIM monomers indicates that loop 1 and loop 3 (residues 13 to 16 and 69 to 76, respectively), both critical components of the interface of TIMs, are altered in the mutant protein (Fig 8B and S5 Fig). In the crystal structure of this mutant, the lateral chain of the introduced D15 rotates from its original position in the WT structure (Fig 8, panels C-D) to establish a new salt bridge with the adjacent R17 of its own subunit and S79 of the adjacent monomer (Fig 8D). It is noteworthy that R17 has, in WT HsTIM, cross-subunit interactions with the side chains of T70 and N71 (Fig 9A), which are lost in the N15D mutant (Fig 9B). Overall, just these few new interactions of D15 (R17 and S79, Fig 8D) trigger the conformational change of loop 1 and the concomitant loss of interactions with loop 3, which finally provoke the perturbation of the HsTIM interface.

As a consequence of the change in the interface arrangement, seven inter-subunit hydrogen bonds per chain are lost in the N15D mutant in comparison to the WT structure (Fig 9, panels A-B); the change is accompanied by a reduction in the interface area from 1712 Å^2^ in the WT structure to 1256 Å^2^ in the mutant. This reduction in 456 Å^2^ represents 27% less contact area between monomers in the N15D structure (Fig 9, panels C-D). The diminution in the interface area is reflected in the loss of interfacial contacts (Fig 9, panels C-D, S1 Table). Two residues in particular, M14 from loop 1 and T75 from loop 3, are the landmarks for these weakened interactions (Fig 9, panels C-D). In all known TIM structures, residue 14 (or the equivalent residue in TIMs from other species) is tightly packed inside loop 3 of the adjacent subunit as in the WT structure of HsTIM (S6 Fig, panels A and B). In contrast, in the crystal structure of the N15D mutant, the lateral chain of M14 flips toward and occupies a hydrophobic cavity in its own subunit, leaving the contralateral loop 3 unoccupied and with an altered conformation (S6 Fig, panels C-D). Interestingly, the interactions of M14 with its new environment are even larger than those established originally with loop 3 in the WT structure (S2 Table).

**Fig 10:**
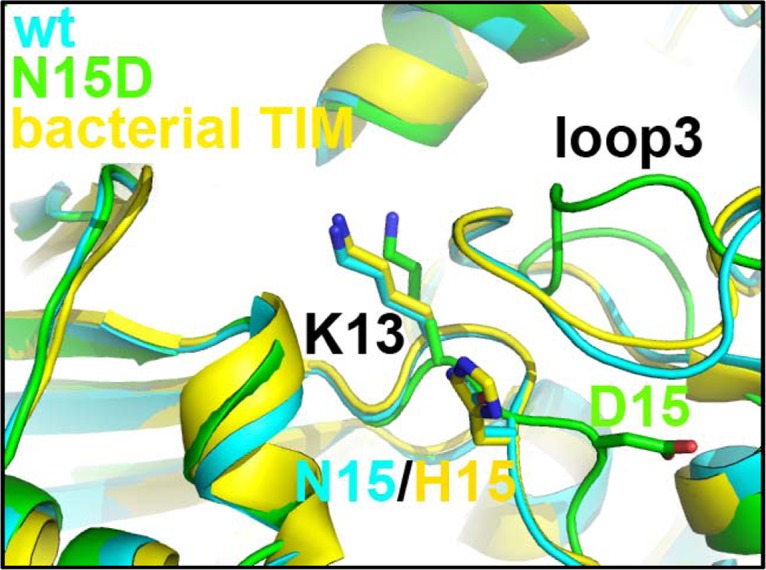
The mutant N15D decrease the activity of HsTIM. The comparison between the wild type and the N15D mutant in the region of the catalytic residue K13 shows a different, inactive conformation. In all known TIMs, the structure of this region is conserved, even when compared to an enzyme from a bacterial source (TIM from Thermus thermophilus, PDB code 1YYA), which has a histidine residue in the equivalent position of HsTIM residue 15.

Most importantly, the interface changes in the N15D mutant can be tracked to the active site of the mutated protein (Fig 10). In the N15D structure, the catalytic residue K13 moves from its canonical position out of the Ramachandran plot as in all TIM enzymes (phi/psi angles 51°/-148°, respectively) to an allowed conformation in the mutant (phi/psi angles -67°/-46°). The change can be clearly observed in the crystal structure as a displacement of 2.6 Å of the epsilon amino group of K13 (Fig 10). The result is consonant with the disrupted catalytic properties of the N15D mutant.

In summary, the crystal structure of the N15D mutant allows to explain how the change of the asparagine amide group to the carboxylate group of aspartic acid disrupts the quaternary structure of HsTIM and the architecture of the active site, rendering an unstable protein with altered catalytic properties. To our knowledge, these dramatic conformational changes had never been reported for any TIM. In addition, for the first time, this crystal structure provides high-resolution molecular data bridging deamidation, conformational changes and physiological effects.

### The amino acids responsible for the detrimental changes linked to deamidation are conserved in mammalian TIMs, but not in other phylogenetic groups

A phylogenetic analysis of TIM sequences was performed in order to explore the evolutionary correlation of amino acids contributing to the terminal marking by deamidation. Two hundred twenty one sequences spanning a wide range of taxon orders (70 belonging to Eukarya, 131 to Bacteria and 20 to Archaea) were retrieved from the curated Refseq database at the NCBI. The multiple sequence alignment generated is provided as Supplementary material (S1 TIM Sequences Alignment). The calculated phylogenetic tree mostly coincides with the general topology of the universal phylogenetic tree inferred from total genome analysis [43, 44], with the three main domains (Bacteria, Archaea, and Eukarya) clearly defined (S7 Fig). Only a small group of 9 sequences from Bacteria were positioned outside of their expected position and found beside the Archaea domain; those from the phylum of proteobacteria (*Francisella*, *Neisseria*, *Wolbachia*, *Orientia*, *Campylobacter* and *Helicobacter*) and from the phylum of firmicutes (*Mycoplasma* and *Ureaplasma*) (S7 Fig). The anomalous position of these sequences outside of their expected positions was not explored any further.

We investigated the distribution of asparagine-glycine pairs (NG) in the 221 selected sequences (S7 Fig). In the Eukarya domain, 128 NG pairs were found in the 70 sequences analyzed (average 1.8 NG pairs per each TIM sequence); in Bacteria, 91 NG pairs were distributed in the 131 sequences studied (0.7 NG/TIM), whereas in Archaea, only 4 pairs were found in the 20 sequences analyzed (0.2 NG/TIM). The differential distribution of NG pairs seems not to result from variations in the total asparagine content. There are 143 asparagine residues in 20 sequences from Archaea (7.2 N/TIM), and 804 asparagine residues in 70 Eukarya sequences (11.5 N/TIM) and in Bacteria, 1279 asparagine residues are present in 131 sequences (9.8 N/TIM). The analysis indicates that, in the case of TIM, NG pairs are not evenly distributed through the domains of life.

In regard to the distribution of NG pairs previously related to the terminal marking of TIM (NG pairs 15–16 and 71–72, using HsTIM numbering), it is relevant to highlight that the 15–16 pair is highly conserved in the Eukarya domain, is not present in the Archaea group and is irregularly found in Bacteria (S8 Fig). In contrast, the 71–72 pair is only consistently conserved in mammals, is not found in Archaea and is scattered throughout the rest of the sequences (S8 Fig). In addition, it is interesting to note that natural substitutions of N15 only include a small number of amino acids (N, H, K, F, Y, and A), whereas in position 71, 19 of the 20 amino acids occurs naturally (excepting Q) (TIM sequences alignment.pdf). These results show a higher conservation of the 15–16 pair over the 71–72 pair, and a smaller variability of position 15 in comparison with position 71.

**Fig 11:**
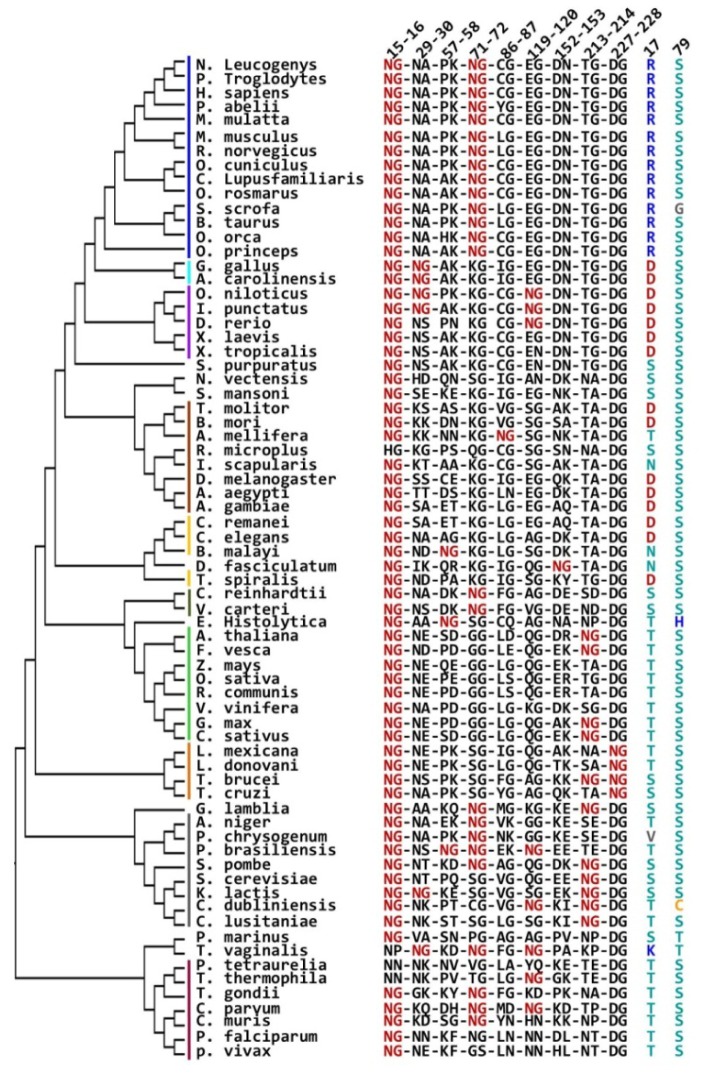
Phylogenetic analysis of TIM; relationship between R17, S79 and NG pairs in Eukarya TIMs. The Eukarya domain was extracted from the complete phylogenetic tree and analyzed. NG pairs present in Eukarya sequences are shown in red; residues occurring at positions 17 and 79 are shown in the right columns. Numbering according to the HsTIM sequence is shown at the top of the figure. Color bars indicate major evolutionary grades with a color code as follows: mammals, blue; sauropsidae (reptiles and birds), cyan; anamniotae (fishes and amphibians), purple; arthropodae, brown; nematodae, yellow; green algae, olive; plants, green; kinetoplastidae, orange; fungi, gray; chromalveolata, red.

Finally, considering that the terminal marking mechanism by deamidation seems to depend on the presence of R17 and S79, and that this mechanism is not general for TIM (recall that deamidation is relevant for the human and rabbit enzymes, but not for the yeast or chicken proteins), it was interesting to ask if a phylogenetic relationship between the deamidating pairs, R17 and S79, exists (Fig 11). The results show that the NG pair 15–16 is highly conserved (in 66 of 70 Eukarya sequences) in comparison with the pair 71–72 (which is only found in 25 of 70 sequences, mainly in mammals); in contrast, the remaining NG pairs are poorly conserved (Fig 11). In addition, it can be observed that S79 is highly conserved (it occurs in 65 of the 70 Eukarya sequences) whereas, in contrast, R17 is only found in mammal sequences and is completely absent in the rest of Eukarya TIMs. In fact, positive amino acids are not found in position 17, excepting *T*. *vaginalis* where lysine is present (Fig 11). The results indicate a close relationship between R17 and the NG pair 15–16 in the mammalian enzymes, but not for other phylogenetic groups. In addition, it is interesting to note that the NG pair 71–72 is also consistently present in mammalian enzymes, but in contrast to R17, it is also found in lower branches as those of apicomplexans, fungi, green algae, *G*. *lamblia*, and *T*. *vaginalis* (Fig 11).

## Discussion

This study describes the effects of asparagine deamidation at positions 15 and 71 in HsTIM. Deamidations were mimicked using site directed mutagenesis; a similar approach has been recently used to model the effects of deamidation on the immunogenicity of the protective antigen of the anthrax toxin [45]; or in superoxide dismutase to mimic deamidations leading the conversion of the protein to a neurotoxic isoform [46]. Thus, it can be safely assumed that mutagenesis can be used to study deamidations at a residue-specific level.

The spectroscopic results show that the asparagine to aspartic acid mutation does not induce gross conformational changes in HsTIM (S2 Fig); however, the kinetic and stability assays (Figs 2, 3, 4, 5 and 6) indicate that this mutation has disparate effects on the catalytic properties and the stability of the enzyme. Whereas the N71D mutation is practically innocuous, the single N15D mutation displays all the changes associated with terminal marking; *i*.*e*. decreased catalysis, diminished thermal stability, increased susceptibility to proteolysis and loosening of the whole three-dimensional structure, including the dimeric interface. From these set of experiments, three points deserve consideration: i) Deamidation of N71 only has effects on the catalytic properties of HsTIM in conjunction with the deamidation of N15. ii) It is notorious that deamidation of N71 does not contribute to the loss of stability of HsTIM; neither alone nor in the presence of deamidated N15. iii) In contrast to the terminal marking mechanism of destabilization by charge repulsion at the TIM interface [20], which requires the mandatory deamidation of both N15 and N71, it was shown here that single deamidation of N15 is sufficient to disrupt the catalytic and structural properties of HsTIM. Therefore, it can be concluded that N15 is the main contributor to the terminal marking by deamidation in HsTIM. Also, the detrimental catalytic, structural and stability effects of deamidation at position 15 can be explained with the crystal structure of the N15D mutant, which shows the disturbance of the dimer interface that extends down to the active site (Figs 8, 9 and 10). The close correlation between the interface and the active site in TIM has been extensively addressed [15–19]. Decreased substrate affinity for the N15D mutant can be probably explained by the 2.6 Å shift in the position of the of the epsilon amino group of K13 (Fig 10). This is supported by the corresponding yeast TIM mutant K12G, which shows little or no approach to saturation by substrate [47].

Altogether, the experimental results allow us to reassess the terminal marking mechanism by deamidation on HsTIM, a model prevailing for more than 30 years [20]. Our data reveal a mechanism that is directed by the deamidation of N15, which in the carboxylic acid form of aspartic acid, interacts with nearby residues R17 and S79. These new interactions induce the conformational displacement of M14 and consequently the perturbation of loop 3, which finally provokes the disturbance of the dimer assembly and disrupts the architecture of the active site. It is impressive to see that a whole set of interactions are disrupted just because D15 looks for its own, “selfish” stability. This probably would not occur if a residue other than glycine were present after the aspartic acid, because the relative flexibility offered by this small amino acid, possibly allows conformational search.

The phylogenetic analysis of TIM sequences allow us to make evolutionary inferences about the occurrence of the terminal marking mechanism by deamidation of N15. The distribution of NG pairs across the phylogenetic tree of TIMs indicates that these pairs are diminished in the archaea domain, although the reduction seems not to be the consequence of a decrease in the asparagine content. The amount of asparagine residues in TIMs from Archaea is slightly lower than in TIMs from Eukarya and Bacteria, which parallels the total asparagine content in these groups as estimated by the average percent amino acid content in complete proteomes [48]. Therefore, it can be hypothesized that NG pairs have been negatively selected in TIMs from the Archaea domain, which could be probably related to the presence of extremophiles in this group.

The original terminal marking mechanism directed by charge repulsion at the TIM interface, requires the deamidation of both NG pairs 15–16 and 71–72 [20]. Phylogenetic analysis of these pairs show that pair 15–16 is more conserved than pair 71–72 (S8 Fig). In addition, N15 is naturally much less variable than N71 (TIM sequences alignment.pdf). These results suggest an evolutionarily conserved role for N15. Since the deamidation of N15 directs the terminal marking mechanism for TIM, the question arises if conserved asparagine residues are selected as “hot spots” to introduce deamidation in order to trigger structural or functional effects on proteins.

Based on the amino acid sequence of chicken and yeast TIMs, which lack NG pair 71–72 and do not deamidate, it was proposed that deamidation of NG pair 71–72 is a prerequisite for deamidation of the NG pair 15–16 [20–22]. The strict conservation of both pairs in mammalian enzymes (Fig 11 and S8 Fig) could support its importance for this group of enzymes. However, as shown here, deamidation of N71 has negligible influence on the catalysis and the stability of HsTIM (Figs 2, 3, 4, 5 and 6), or on the deamidation rate of N15 (Fig 7). In agreement with this last point, computational studies indicate that N71 and N15 deamidate independently [48, 49]. The deamidation coefficients (C_D_) for the asparagine residues in HsTIM indicate that N71 and N15 deamidate at similar rates [49]. The result is consistent with the results from recent molecular dynamics simulations showing that both asparagine residues have the same probabilities to initiate deamidation, and that the deamidation of N15 is not influenced by the deamidation of N71 [50].

In contrast, the dependence of the terminal marking mechanism on R17 is supported by the experimental results and the phylogenetic analysis. The crystal structure of the N15D mutant indicates that R17 is instrumental for the terminal marking mechanism by deamidation (Fig 8). In addition, the phylogenetic analysis of the Eukarya TIM sequences shows that R17 is only present in the mammalian sequences (Fig 11). It is therefore reasonable to propose that the terminal marking mechanism by deamidation of N15 is only possible in mammalian TIMs. This suggestion is supported by the aforementioned studies on the enzymes of chicken or yeast, both of which lack R17, and for which deamidation plays no role in the terminal marking of these enzymes.

In summary, a refined mechanism for the terminal marking process of HsTIM by deamidation has been presented here. The mechanism relies on the single deamidation of N15 and the presence of S79 and R17, and involves the rearrangement of the interface and the active site. This mechanism is only possible in mammalian TIMs, due to the exclusive presence of R17 in this group of organisms. It is plausible that evolution selected conserved (important) asparagine residues as “hot spots” for which deamidation triggers structural or functional effects on proteins.

## Supporting Information

S1 Fig Native gel electrophoresis of WT and deamidated mutants. Native electrophoresis (7%) was carried out in Tris-Glycine pH 8.5 for 3 hours at 7 mA with the cathode placed at the bottom. Ten micrograms of protein were loaded onto each lane; staining was performed with Coomassie brilliant blue.
(JPG)

S2 Fig Spectroscopic characterization of WT HsTIM and the mutants. (A) Far-UV (190–260 nm) circular dichroism spectra were performed at 25°C in mixtures containing 100 μg/mL of protein in 25 mM phosphate buffer, pH 7.4. (B) Intrinsic fluorescence spectra were recorded from 310 to 500 nm after excitation at 280 nm; excitation and emission slits were 10 and 7.5 nm, respectively. Mixtures contained 100 μg/mL of protein in 25 mM phosphate buffer, pH 7.4. For all cases, blanks without protein were subtracted from the experimental ones; each spectrum was the average of three replicated scans.
(JPG)

S3 Fig Electron density maps around residues D15 and D71 in the HsTIM mutants. Simulated annealed omit electron density maps contoured at 1.5σ around D15 in the N15D mutant (A), and D71 in the N71D mutant (B).
(JPG)

S4 Fig Structure of HsTIM N71D in comparison with WT HsTIM. (A) Overall structural superposition of WT HsTIM (green) and the N71D HsTIM mutant (orange). The RMSD for all the atoms in these structures is 0.56 Å. (B) Structural comparison of the N71D mutant and the WT HsTIM structure in the vicinity of N71 or D71 residues; the electronic density map for the N15 residue in the N71D structure, showing the two conformations of the N15 side chain, is shown in (C). (D) Structural comparison of the active site in the WT and in the N71D mutant structures. Residues are shown as stick models.
(JPG)

S5 Fig Superposition of the monomers of WT HsTIM and the mutant N15D. Cα RMS deviations, calculated between the monomers of the wild-type enzyme (PDB code 2JK2) and the mutant N15D (PDB code 4UNK).
(JPG)

S6 Fig Structural modifications of M14 and loop 3 in the crystal structure of HsTIM N15D. Structural comparison of TIM interfaces in the WT enzyme (A-B) and in the N15D mutant (C-D). M14 residues are shown as stick models and loops 3 are highlighted in red color. Panels (A) and (C) shows overall views of TIM dimers, whereas in (B) and (D) close up views of interfacial regions are shown.
(JPG)

S7 Fig Phylogenetic analysis of TIM; distribution of NG pairs. A 221 TIM sequences alignment generated with Clustal_X (39) was used to calculate the phylogenetic tree with Mega 5.21 (41) by the maximum likelihood method. Archaea, Eukarya and Bacteria domains are indicated in the red, blue and black branches, respectively. Green dots indicate the number of NG pairs present in each sequence.
(JPG)

S8 Fig Phylogenetic analysis of TIM; ocurrence of NG pairs 15–16 and 71–72. A 221 TIM sequences alignment generated with Clustal_X (39) was used to calculate the phylogenetic tree with Mega 5.21 (41) by the maximum likelihood method. Archaea, Eukarya and Bacteria domains are indicated in the red, blue and black branches, respectively. Purple dots indicate the presence of a NG pair in position 15–16 (HsTIM numbering), whereas blue dots indicate sequences containing NG pairs at position 71–72.
(JPG)

S1 Table Lost inter-subunit hydrogen bonds in the crystal structures of the WT and the N15D HsTIM. (JPG)

S2 Table Intersubunit contact area per residue in the crystal structures of the WT and the N15D HsTIM. (JPG)

S1 TIM Sequences Alignment Progressive multiple sequence alignment of TIM sequences was performed as indicated in Material and Methods. (PDF)

## References

[1] Robinson NE, Robinson AB. Molecular Clocks: Deamidation of Asparaginyl and Glutaminyl Residues in Peptides and Proteins. Cave Junction, Oregon: Althouse Press; 2004

[2] Robinson NE, Robinson AB. Use of Merrifield solid phase peptide synthesis in investigations of biological deamidation of peptides and proteins. Biopolymers. 2008; 90:297–306. 17896348 10.1002/bip.20852

[3] Robinson AB, McKerrow JH, Cary P. Controlled deamidation of peptides and proteins: an experimental hazard and a possible biological timer. Proc Natl Acad Sci USA. 1970; 66:753–757. 5269237 10.1073/pnas.66.3.753 PMC283114

[4] Robinson AB. Evolution and the distribution of glutaminyl and asparaginyl residues in proteins. Proc Natl Acad Sci USA. 1974; 71:885–888. 4522799 10.1073/pnas.71.3.885 PMC388120

[5] Robinson AB, Rudd CJ. Deamidation of glutaminyl and asparaginyl residues in peptides and proteins. Curr Top Cell Regul. 1974; 8:247–295. 4371091 10.1016/b978-0-12-152808-9.50013-4

[6] Robinson AB. Molecular clocks, molecular profiles, and optimum diets: three approaches to the problem of aging. Mech Ageing Dev. 1979; 9:225–236. 374893 10.1016/0047-6374(79)90101-5

[7] Robinson NE, Robinson AB. Molecular clocks. Proc Natl Acad Sci USA. 2001; 98:944–949. 11158575 10.1073/pnas.98.3.944 PMC14689

[8] Robinson NE. Protein deamidation. Proc Natl Acad Sci USA. 2002; 99:5283–5288. 11959979 10.1073/pnas.082102799 PMC122761

[9] Noguchi S. Structural changes induced by the deamidation and isomerization of asparagine revealed by the crystal structure of *Ustilago sphaerogena* ribonuclease U2B. Biopolymers. 2010;93:1003–1010. 10.1002/bip.21514 20623666

[10] Eschenburg S, Schönbrunn E. Comparative X-ray analysis of the un-liganded fosfomycin-target murA. Proteins. 2000;40:290–298. 10842342 10.1002/(sici)1097-0134(20000801)40:2<290::aid-prot90>3.0.co;2-0

[11] Rester U, Moser M, Huber R, Bode W. L-Isoaspartate 115 of porcine beta-trypsin promotes crystallization of its complex with bdellastasin. Acta Crystallogr D Biol Crystallogr.2000;56:581–588. 10771427 10.1107/s0907444900003048

[12] Esposito L, Vitagliano L, Sica F, Sorrentino G, Zagari A, Mazzarella L. The ultrahigh resolution crystal structure of ribonuclease A containing an isoaspartyl residue: hydration and sterochemical analysis. J Mol Biol. 2000;297:713–732. 10731423 10.1006/jmbi.2000.3597

[13] Noguchi S, Miyawaki K, Satow Y. Succinimide and isoaspartate residues in the crystal structures of hen egg-white lysozyme complexed with tri-N-acetylchitotriose. J Mol Biol. 1998;278:231–238. 9571046 10.1006/jmbi.1998.1674

[14] Aritomi M, Kunishima N, Inohara N, Ishibashi Y, Ohta S, Morikawa K. Crystal structure of rat Bcl-xL. Implications for the function of the Bcl-2 protein family. J Biol Chem. 1997;272:27886–27892. 9346936 10.1074/jbc.272.44.27886

[15] Albery WJ, Knowles JR. Evolution of enzyme function and the development of catalytic efficiency. Biochemistry. 1976;15:5631–5640. 999839 10.1021/bi00670a032

[16] Knowles JR. Enzyme catalysis: not different, just better. Nature. 1991;350:121–124. 2005961 10.1038/350121a0

[17] Zárate-Pérez F, Chánez-Cárdenas ME, Vázquez-Contreras E. The folding pathway of triosephosphate isomerase. Prog Mol Biol Transl Sci. 2008;84:251–267. 10.1016/S0079-6603(08)00407-8 19121704

[18] Wierenga RK, Kapetaniou EG, Venkatesan R. Triosephosphate isomerase: a highly evolved biocatalyst. Cell Mol Life Sci. 2010;67:3961–3982. 10.1007/s00018-010-0473-9 20694739 PMC11115733

[19] Richard JP. A paradigm for enzyme-catalyzed proton transfer at carbon: triosephosphate isomerase. Biochemistry. 2012;51:2652–2661. 10.1021/bi300195b 22409228 PMC3319633

[20] Yuan PM, Talent JM, Gracy RW. Molecular basis for the accumulation of acidic isozymes of triosephosphate isomerase on aging. Mech Ageing Dev. 1981;17:151–162. 7311622 10.1016/0047-6374(81)90081-6

[21] Yüksel KU, Gracy RW. *In vitro* deamidation of human triosephosphate isomerase. Arch Biochem Biophys. 1986;248:452–459. 3740839 10.1016/0003-9861(86)90498-4

[22] Gracy RW, Talent JM, Zvaigzne AI. Molecular wear and tear leads to terminal marking and the unstable isoforms of aging. J Exp Zool. 1998;282:18–27. 9723162

[23] Sun AQ, Yüksel KU, Gracy RW. Terminal marking of triosephosphate isomerase: consequences of deamidation. Arch Biochem Biophys. 1995;322:361–368. 7574709 10.1006/abbi.1995.1476

[24] Schägger H, von Jagow G. Tricine-sodium dodecyl sulfate-polyacrylamide gel electrophoresis for the separation of proteins in the range from 1 to 100 kDa. Anal Biochem. 1987;166:368–379. 2449095 10.1016/0003-2697(87)90587-2

[25] McLellan T. Electrophoresis buffers for polyacrylamide gels at various pH. Anal Biochem. 1982;126:94–99. 7181120 10.1016/0003-2697(82)90113-0

[26] Oesper P, Meyerhof O. The determination of triose phosphate isomerase. Arch Biochem. 1950;27:223–233. 15419792

[27] Rodríguez-Almazán C, Arreola R, Rodríguez-Larrea D, Aguirre-López B, de Gómez-Puyou MT, Pérez-Montfort R, et al Structural basis of human triosephosphate isomerase deficiency: mutation E104D is related to alterations of a conserved water network at the dimer interface. J Biol Chem. 2008;283:23254–23263. 10.1074/jbc.M802145200 18562316

[28] Enríquez-Flores S, Rodríguez-Romero A, Hernández-Alcántara G, Oria-Hernández J, Gutiérrez-Castrellón P, Pérez-Hernández G, et al Determining the molecular mechanism of inactivation by chemical modification of triosephosphate isomerase from the human parasite *Giardia lamblia*: a study for antiparasitic drug design. Proteins. 2011;79:2711–2724. 10.1002/prot.23100 21786322

[29] Aguirre B, Costas M, Cabrera N, Mendoza-Hernández G, Helseth DL Jr, Fernández P, et al A ribosomal misincorporation of Lys for Arg in human triosephosphate isomerase expressed in *Escherichia coli* gives rise to two protein populations. PLoS One. 2011;6:e21035–e21044 10.1371/journal.pone.0021035 21738601 PMC3125179

[30] Enriquez-Flores S, Rodriguez-Romero A, Hernandez-Alcantara G, de la Mora-de la Mora I, Gutierrez-Castrellon P, Carvajal K, et al Species-specific inhibition of *Giardia lamblia* triosephosphate isomerase by localized perturbation of the homodimer. Mol Biochem Parasitol. 2008;157:179–186. 18077010 10.1016/j.molbiopara.2007.10.013

[31] Reyes-Vivas H, Martínez-Martínez E, Mendoza-Hernández G, López-Velázquez G, Pérez-Montfort R, Tuena de Gómez-Puyou M, et al Susceptibility to proteolysis of triosephosphate isomerase from two pathogenic parasites: characterization of an enzyme with an intact and a nicked monomer. Proteins. 2002;48:580–590. 12112681 10.1002/prot.10179

[32] De la Mora-de la Mora I, Torres-Larios A, Mendoza-Hernández G, Enriquez-Flores S, Castillo-Villanueva A, Mendez ST, et al The E104D mutation increases the susceptibility of human triosephosphate isomerase to proteolysis. Asymmetric cleavage of the two monomers of the homodimeric enzyme. Biochim Biophys Acta. 2013;1834:2702–2711. 10.1016/j.bbapap.2013.08.012 24056040

[33] Collaborative Computational Project, Number 4. The CCP4 suite: programs for protein crystallography. Acta Crystallogr D Biol Crystallogr. 1994;50:760–763. 15299374 10.1107/S0907444994003112

[34] Kabsch W. XDS. Acta Crystallogr D Biol Crystallogr. 2010;66:125–132. 10.1107/S0907444909047337 20124692 PMC2815665

[35] McCoy AJ, Grosse-Kunstleve RW, Adams PD, Winn MD, Storoni LC, Read RJ. Phaser crystallographic software. J Appl Crystallogr. 2007;40:658–674. 19461840 10.1107/S0021889807021206 PMC2483472

[36] Kinoshita T, Maruki R, Warizaya M, Nakajima H, Nishimura S. Structure of a high-resolution crystal form of human triosephosphate isomerase: improvement of crystals using the gel-tube method. Acta Crystallogr Sect F Struct Biol Cryst Commun. 2005;61:346–349. 16511037 10.1107/S1744309105008341 PMC1952429

[37] Murshudov GN, Skubák P, Lebedev AA, Pannu NS, Steiner RA, Nicholls RA, et al REFMAC5 for the refinement of macromolecular crystal structures. Acta Crystallogr D Biol Crystallogr. 2011;67:355–367. 10.1107/S0907444911001314 21460454 PMC3069751

[38] Emsley P, Cowtan K. Coot: model-building tools for molecular graphics. Acta Crystallogr D Biol Crystallogr. 2004;60:2126–2132. 15572765 10.1107/S0907444904019158

[39] Pruitt KD, Tatusova T, Brown GR, Maglott DR. NCBI Reference Sequences (RefSeq): current status, new features and genome annotation policy. Nucleic Acids Res. 2012;40:D130–135. 10.1093/nar/gkr1079 22121212 PMC3245008

[40] Thompson JD, Gibson TJ, Plewniak F, Jeanmougin F, Higgins DG. The CLUSTAL_X windows interface: flexible strategies for multiple sequence alignment aided by quality analysis tools. Nucleic Acids Res. 1997;25:4876–4882. 9396791 10.1093/nar/25.24.4876 PMC147148

[41] Benner SA, Cohen MA, Gonnet GH. Amino acid substitution during functionally constrained divergent evolution of protein sequences. Protein Eng. 1994;7:1323–1332. 7700864 10.1093/protein/7.11.1323

[42] Tamura K, Peterson D, Peterson N, Stecher G, Nei M, Kumar S. MEGA5: Molecular Evolutionary Genetics Analysis using Maximum Likelihood, Evolutionary Distance, and Maximum Parsimony Methods. Mol Biol Evol. 2011;28:2731–2739. 10.1093/molbev/msr121 21546353 PMC3203626

[43] Ciccarelli FD, Doerks T, von Mering C, Creevey CJ, Snel B, Bork, P. Toward automatic reconstruction of a highly resolved tree of life. Science. 2006;311:1283–1287. 16513982 10.1126/science.1123061

[44] Letunic I, Bork P. Interactive Tree Of Life (iTOL): an online tool for phylogenetic tree display and annotation. Bioinformatics. 2007;23:127–128. 17050570 10.1093/bioinformatics/btl529

[45] Verma A, McNichol B, Domínguez-Castillo RI, Amador-Molina JC, Arciniega JL, Reiter K, et al Use of site-directed mutagenesis to model the effects of spontaneous deamidation on the immunogenicity of Bacillus anthracis protective antigen. Infect Immun. 2013;81:278–284. 10.1128/IAI.00863-12 23115046 PMC3536148

[46] Shi Y, Rhodes NR, Abdolvahabi A, Kohn T, Cook NP, Marti AA, et al Deamidation of asparagine to aspartate destabilizes Cu, Zn superoxide dismutase, accelerates fibrillization, and mirrors ALS-linked mutations. J Am Chem Soc. 2013;135:15897–15908. 10.1021/ja407801x 24066782

[47] Go MK, Koudelka A, Amyes TL, Richard JP. Role of Lys-12 in catalysis by triosephosphate isomerase: a two-part substrate approach. Biochemistry. 2010;49:5377–5389. 10.1021/bi100538b 20481463 PMC2890037

[48] Michelitsch MD, Weissman JS. A census of glutamine/asparagine-rich regions: implications for their conserved function and the prediction of novel prions. Proc Natl Acad Sci USA. 2000;97:11910–11915. 11050225 10.1073/pnas.97.22.11910 PMC17268

[49] Robinson NE, Robinson AB. Prediction of protein deamidation rates from primary and three-dimensional structure. Proc Natl Acad Sci USA. 2001;98:4367–4372. 11296285 10.1073/pnas.071066498 PMC31841

[50] Ugur I, Aviyente V, Monard G. Initiation of the reaction of deamidation in triosephosphate isomerase: investigations by means of molecular dynamics simulations. J Phys Chem B. 2012;116:6288–6301. 10.1021/jp3013305 22574817

