# Supplementary figures and images for: Structural Effects of Protein Aging: Terminal Marking by Deamidation in Human Triosephosphate Isomerase
Source: PLoS One. 2015 Apr 17;10(4):e0123379. doi: 10.1371/journal.pone.0123379 (PMC4401446; doi:10.1371/journal.pone.0123379)

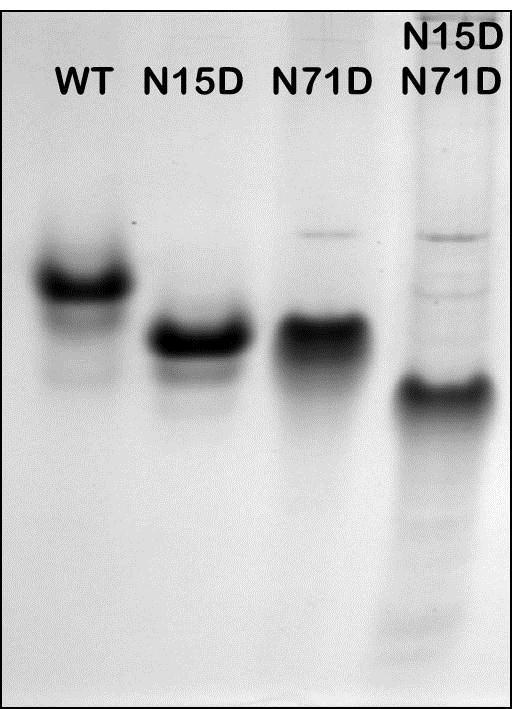

Supplement: S1 Fig — Native electrophoresis (7%) was carried out in Tris-Glycine pH 8.5 for 3 hours at 7 mA with the cathode placed at the bottom. Ten micrograms of protein were loaded onto each lane; staining was performed with Coomassie brilliant blue. (JPG) [file pone.0123379.s001.jpg]

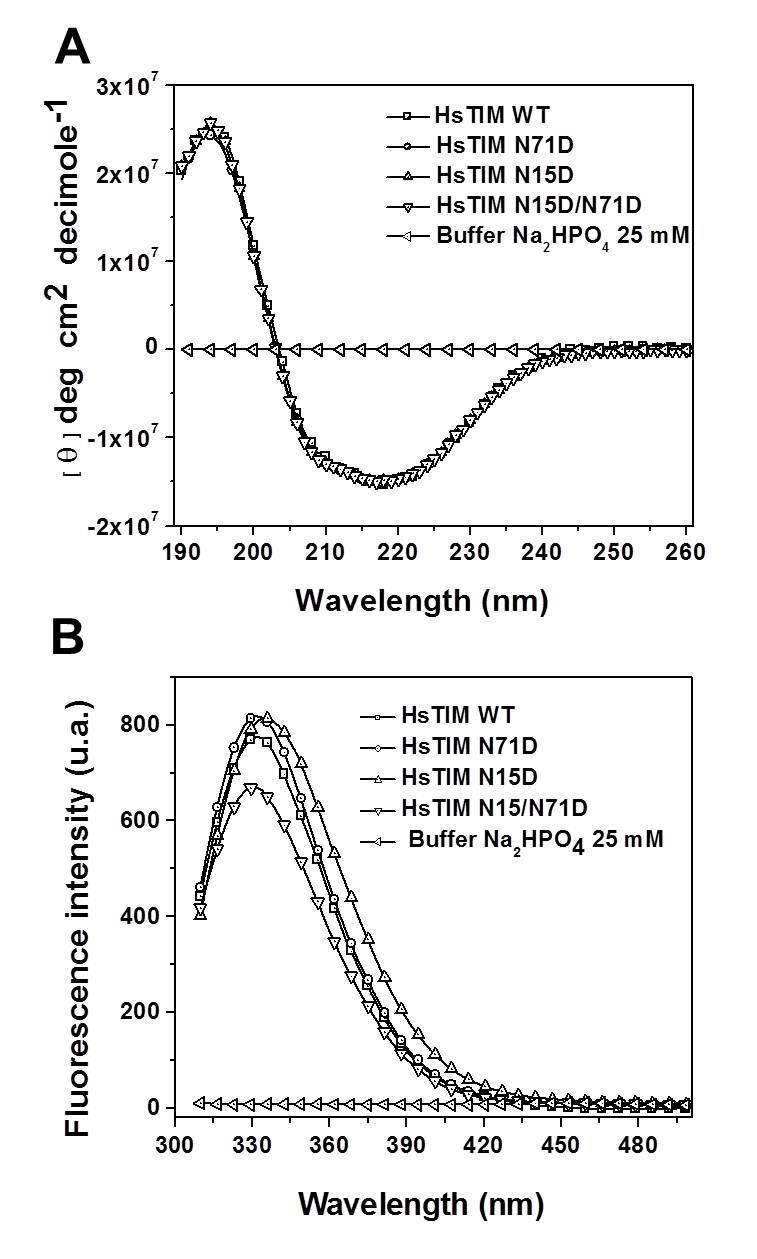

Supplement: S2 Fig — (A) Far-UV (190–260 nm) circular dichroism spectra were performed at 25°C in mixtures containing 100 μg/mL of protein in 25 mM phosphate buffer, pH 7.4. (B) Intrinsic fluorescence spectra were recorded from 310 to 500 nm after excitation at 280 nm; excitation and emission slits were 10 and 7.5 nm, respectively. Mixtures contained 100 μg/mL of protein in 25 mM phosphate buffer, pH 7.4. For all cases, blanks without protein were subtracted from the experimental ones; each spectrum was the average of three replicated scans. (JPG) [file pone.0123379.s002.jpg]

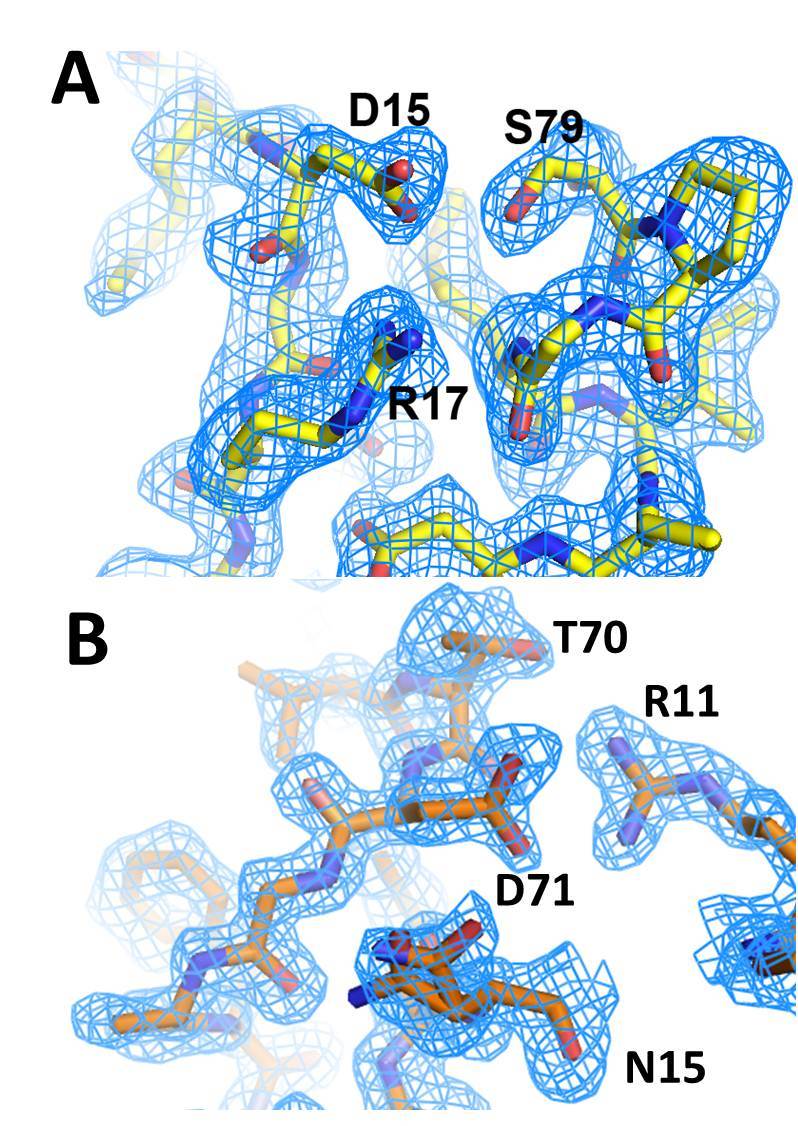

Supplement: S3 Fig — Simulated annealed omit electron density maps contoured at 1.5σ around D15 in the N15D mutant (A), and D71 in the N71D mutant (B). (JPG) [file pone.0123379.s003.jpg]

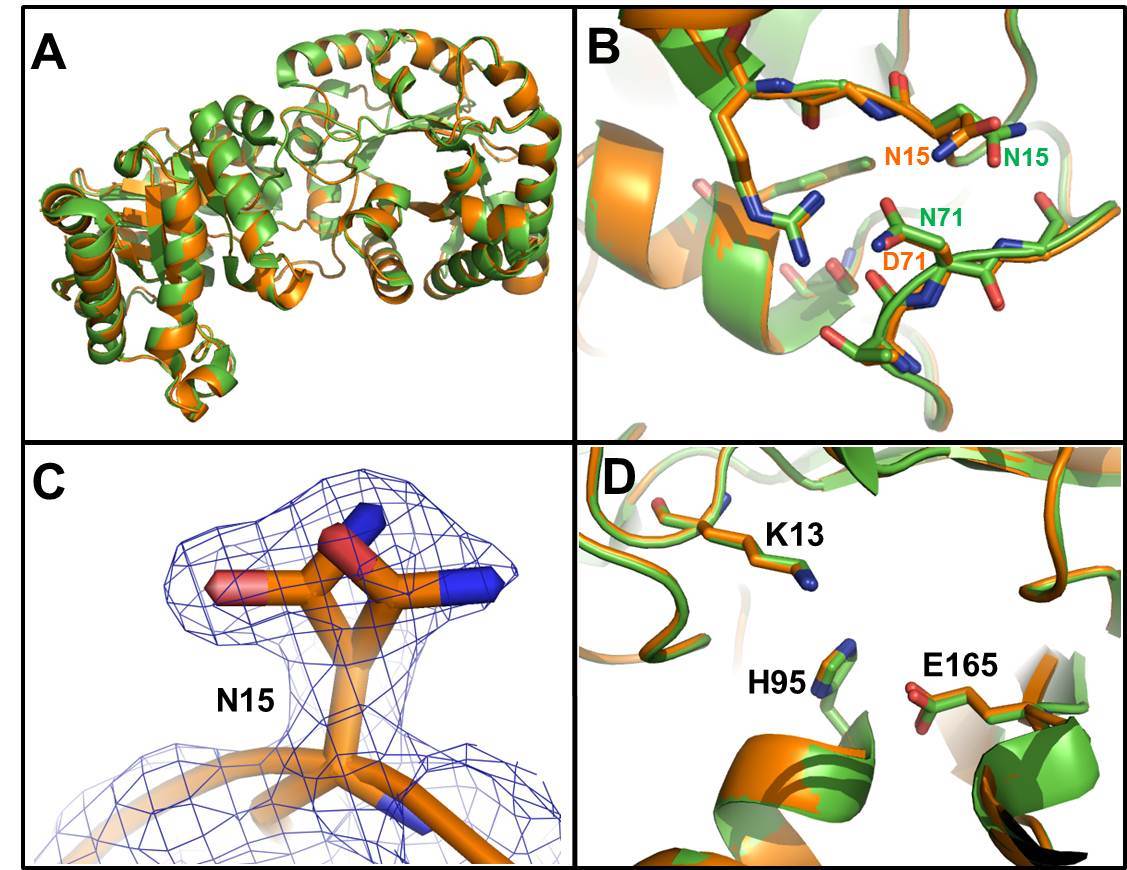

Supplement: S4 Fig — (A) Overall structural superposition of WT HsTIM (green) and the N71D HsTIM mutant (orange). The RMSD for all the atoms in these structures is 0.56 Å. (B) Structural comparison of the N71D mutant and the WT HsTIM structure in the vicinity of N71 or D71 residues; the electronic density map for the N15 residue in the N71D structure, showing the two conformations of the N15 side chain, is shown in (C). (D) Structural comparison of the active site in the WT and in the N71D mutant structures. Residues are shown as stick models. (JPG) [file pone.0123379.s004.jpg]

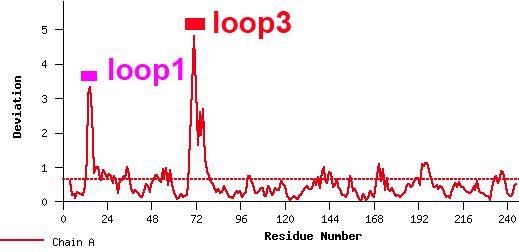

Supplement: S5 Fig — Cα RMS deviations, calculated between the monomers of the wild-type enzyme (PDB code 2JK2) and the mutant N15D (PDB code 4UNK). (JPG) [file pone.0123379.s005.jpg]

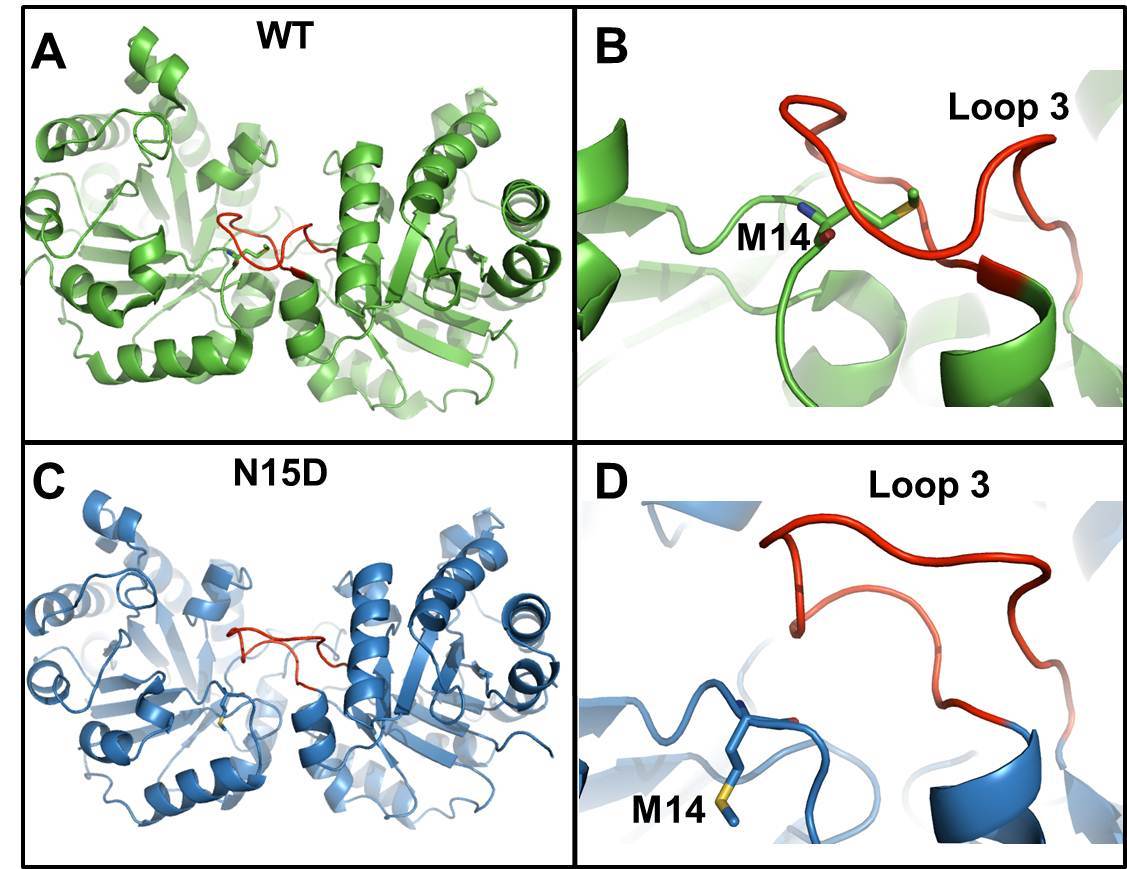

Supplement: S6 Fig — Structural comparison of TIM interfaces in the WT enzyme (A-B) and in the N15D mutant (C-D). M14 residues are shown as stick models and loops 3 are highlighted in red color. Panels (A) and (C) shows overall views of TIM dimers, whereas in (B) and (D) close up views of interfacial regions are shown. (JPG) [file pone.0123379.s006.jpg]

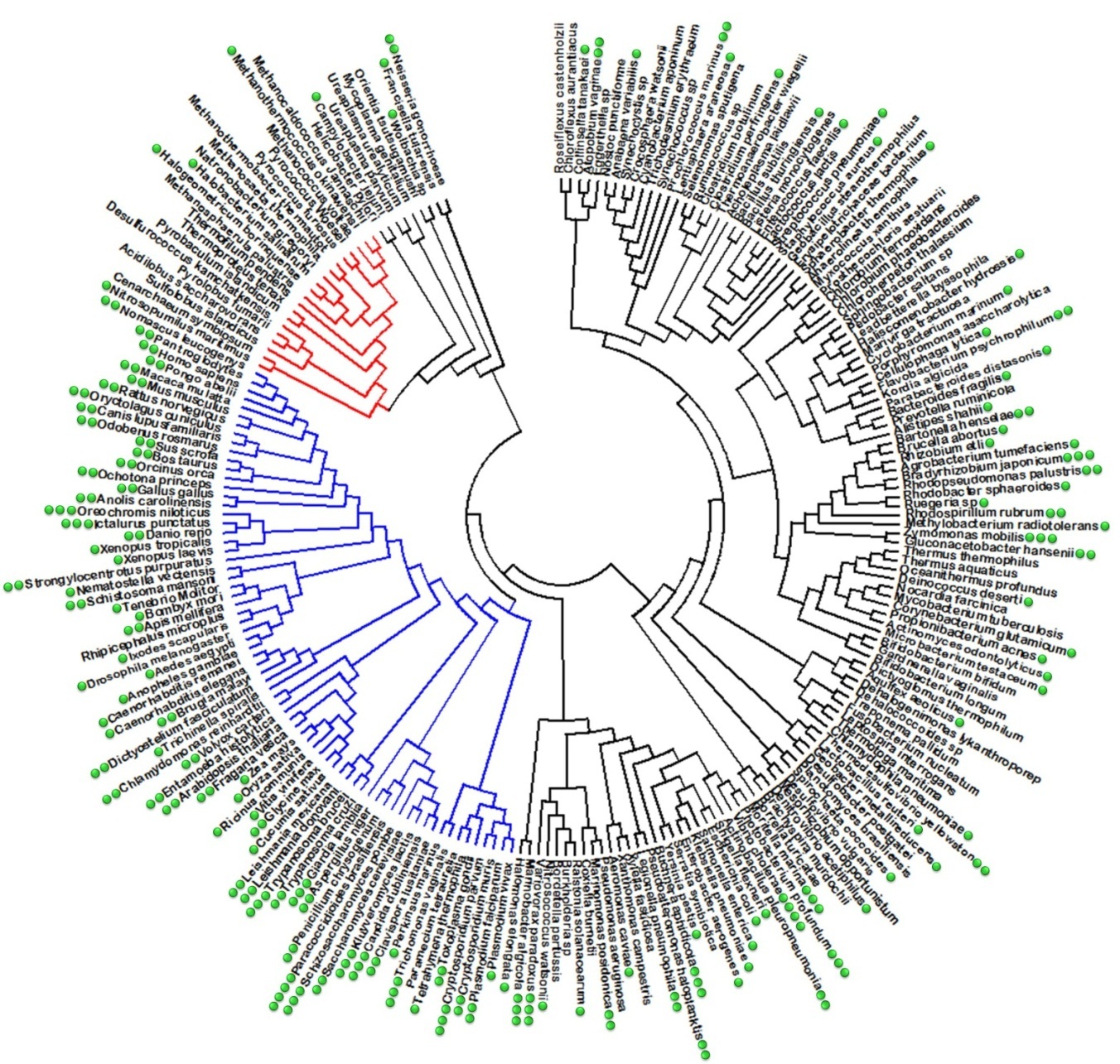

Supplement: S7 Fig — A 221 TIM sequences alignment generated with Clustal_X (39) was used to calculate the phylogenetic tree with Mega 5.21 (41) by the maximum likelihood method. Archaea, Eukarya and Bacteria domains are indicated in the red, blue and black branches, respectively. Green dots indicate the number of NG pairs present in each sequence. (JPG) [file pone.0123379.s007.jpg]

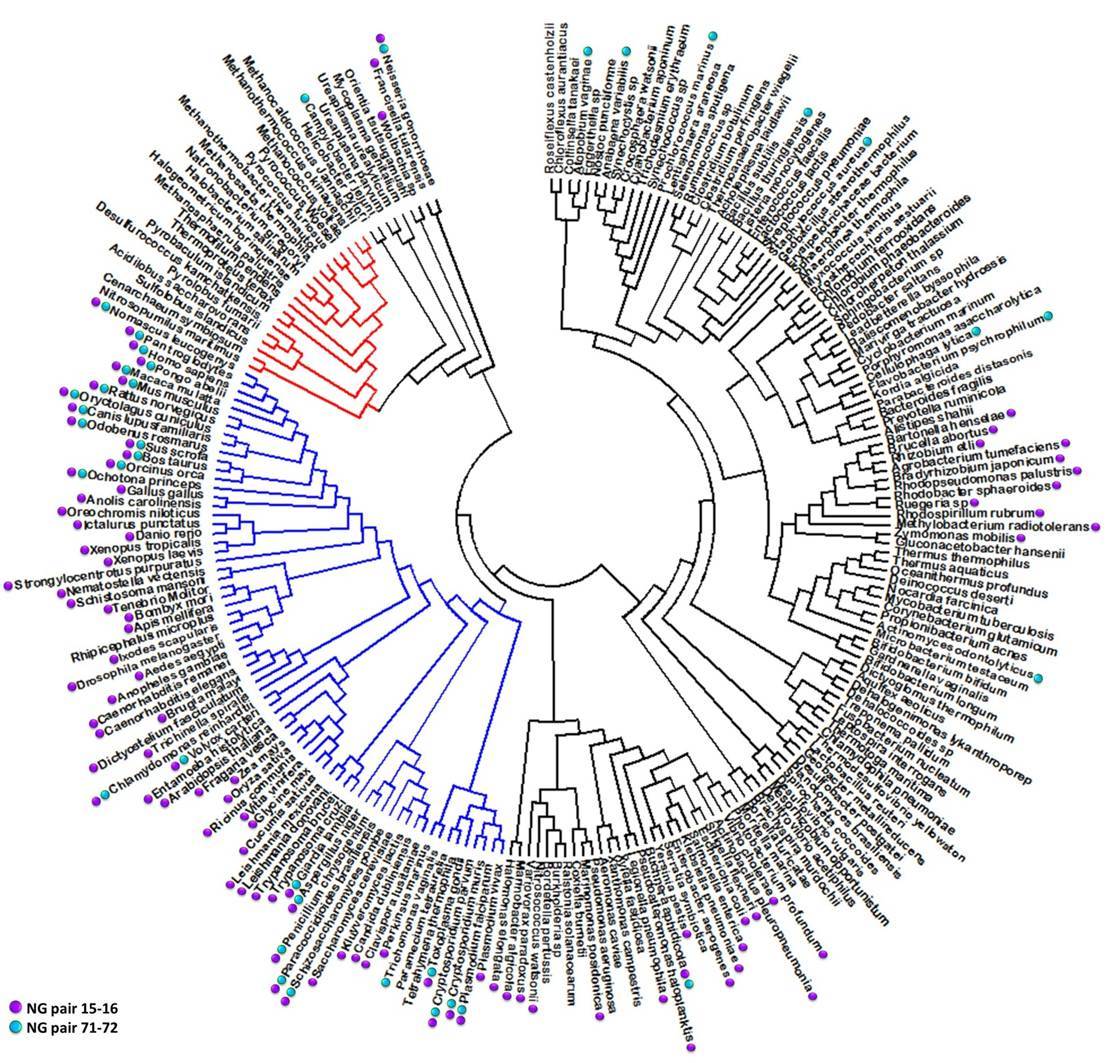

Supplement: S8 Fig — A 221 TIM sequences alignment generated with Clustal_X (39) was used to calculate the phylogenetic tree with Mega 5.21 (41) by the maximum likelihood method. Archaea, Eukarya and Bacteria domains are indicated in the red, blue and black branches, respectively. Purple dots indicate the presence of a NG pair in position 15–16 (HsTIM numbering), whereas blue dots indicate sequences containing NG pairs at position 71–72. (JPG) [file pone.0123379.s008.jpg]

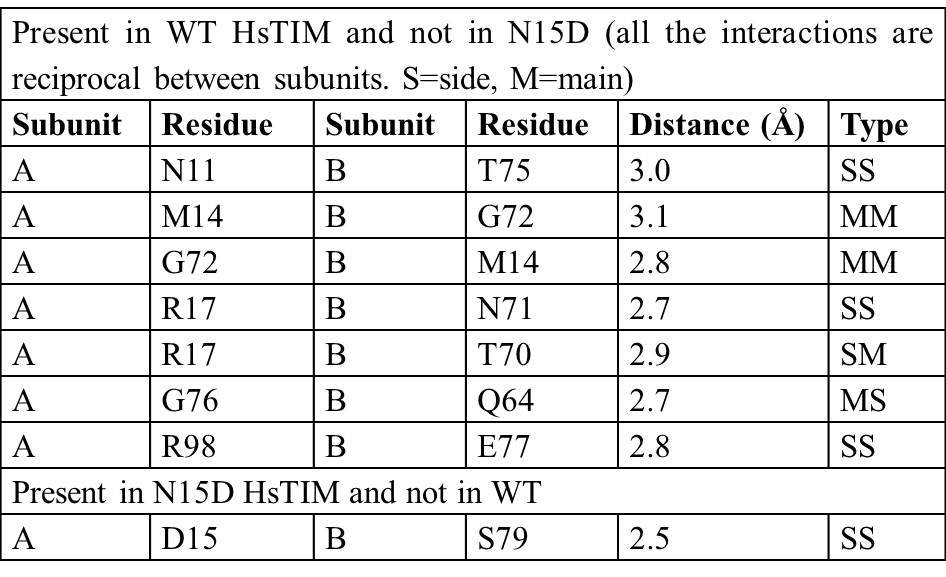

Supplement: S1 Table — (JPG) [file pone.0123379.s009.jpg]

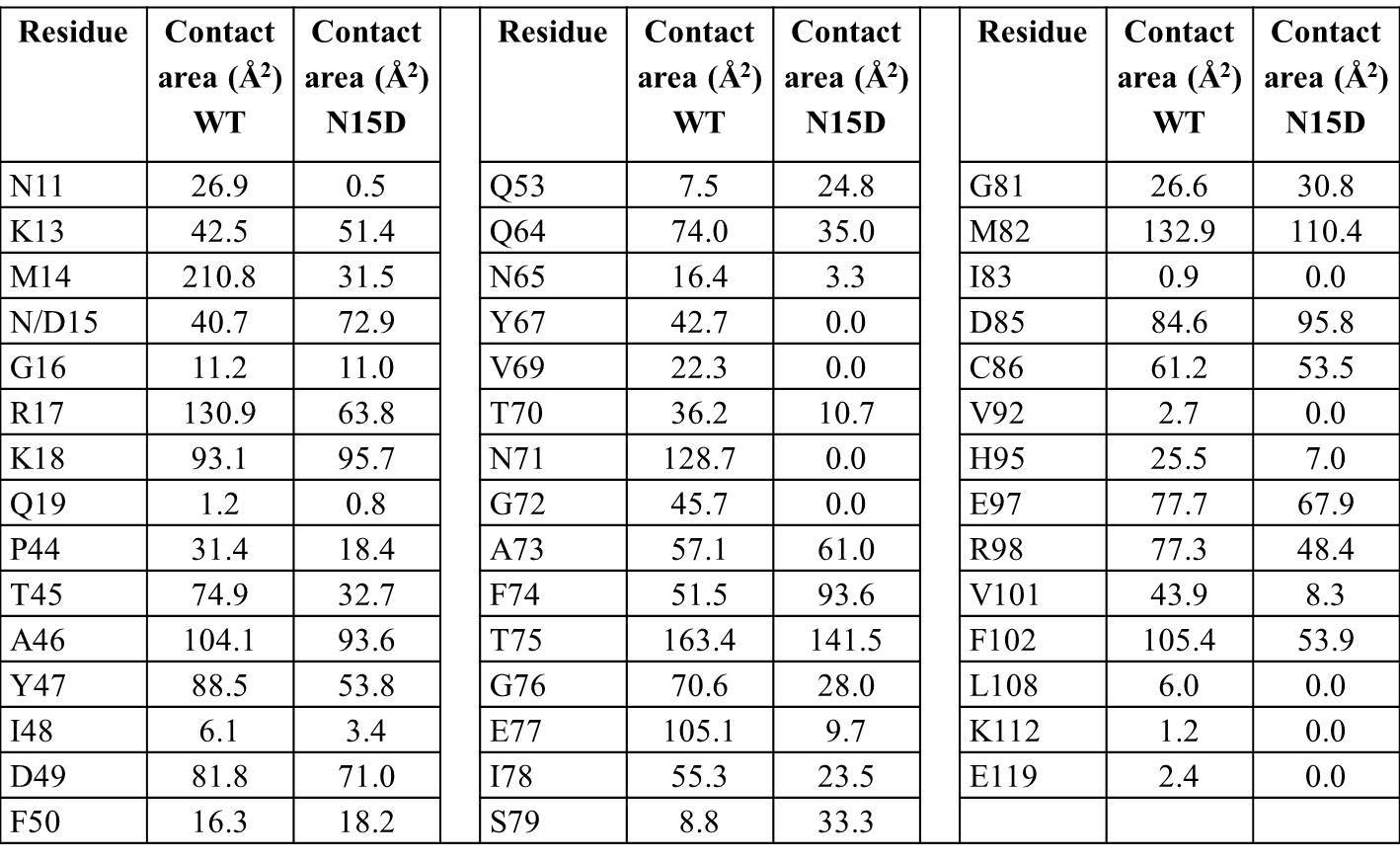

Supplement: S2 Table — (JPG) [file pone.0123379.s010.jpg]
