# Supplementary material for: Structural Effects of Protein Aging: Terminal Marking by Deamidation in Human Triosephosphate Isomerase
Source: PLoS One. 2015 Apr 17;10(4):e0123379. doi: 10.1371/journal.pone.0123379 (PMC4401446; doi:10.1371/journal.pone.0123379)
Supplement: S1 TIM Sequences Alignment — (PDF) [file pone.0123379.s011.pdf]

Progressive multiple sequence alignment of TIM sequences

```
                                         10        20        30        40        50        60        70        80        90       100
                                ....|....|....|....|....|....|....|....|....|....|....|....|....|....|....|....|....|....|....|....|
Roseiflexus_castenholzii        -----------------------------------MRTPLLAGNWKMYKTTGEARELVEGLLHGL-GDVG----D-RKVLVCPPFTALQTVHDLVQG---
Chloroflexus_aurantiacus        -----------------------------------MRIPLIAGNWKMYKTVGEATTLVRDLLAGL-GELS----D-REAIVCPPFTALAAVAALVAD---
Nostoc_punctiforme              -----------------------------------MRKIVIAGNWKMFKTQAETQEFLQGFLPHL-EETP----QGREVILCPPFTDLSVLSKTLHG---
Anabaena_variabilis             -----------------------------------MRKIVIAGNWKMFKTQAESQEFLKEFLPAL-EETP----QEREVLLCVPFTDLAILSQSLHG---
Synechocystis_sp                -----------------------------------MRKIIIAGNWKMHKTQAEAQAFLQGFKPLI-EDAA----ESREVVLCVPFTDLSGMSQQLHG---
Cyanobacterium_aponinum         -----------------------------------MRQIIIAGNWKMHKTQTESLEFLQGFLSHL-EDTP----EERETVLCVPFTCLNFMSKNLHG---
Crocosphaera_watsonii           ---------------------------------MAVGKIIIAGNWKMHKTQAEALEFLTVLKSKV-EETN----ETREIVLCVPFTALSILSKSLHG---
Trichodesmium_erythraeum        -----------------------------------MRKTIIAGNWKMYKTQLEAKEFLEGLMSQL-TKTP----EEREVVLCTPFTALDFMSKILHG---
Synechococcus_sp                -----------------------------------MRKVVIAGNWKMHKTQADASAFVQQLMPQL-ESVP----EEREIVLCAPFTTLGLLVDKLQS---
Prochlorococcus_marinus         -----------------------------------MSKTVIAGNWKMHMTCAQARDFISAYLPLI-KNVP----KGRELVLAPPFTAISTLSEILKG---
Sphaerobacter_thermophilus      -----------------------------------MRKPIVAGNWKMNTSLEEARALAGELRSTL-SAY-----STVERVLIPPFPWIVPLADILDG---
Anaerolinea_thermophila         -----------------------------------MRKKFVAGNWKMNKTAEEARLLLNELIPAL-EPF-----SHVERAVCPPFPYLMMVRHMLEG---
Myxococcus_xanthus              -------------------------------MATARRRKIVAGNWKMNKSVPEALALVRDLRGQV-ASLG----DTVEVVVAPPFVALQPLHVALEG---
Collinsella_tanakaei            -------------------------------MTQNNRTLLIAGNWKMNNDVQAAATLADELVAALDGVDN----G-VEVLVCPPTIDLTTVAAKLDG---
Atopobium_vaginae               -----------------------------------MRNTMIAGNWKMNKTYGEAAELAQGLVRELPGSTG----N-VDVVVCPPTIDLKAVAATLEQSD-
Eggerthella_sp                  -----------------------------------MRKPMMAGNWKMNNTVAEAVVLTQELSNRYTDAWA----DRADVVICPPFVDLKPAKTVLEFDK-
Dictyoglomus_thermophilum       -----------------------------------MRRKIIAANWKMYKTCAETESFIKEFIELSKGY------EEKEIVICPPFTSLYVASKLLKDTA-
Aquifex_aeolicus                -----------------------------------MRR-LIAANWKMNKTVKETEEYINTFLKFVEHP------ESREILICPPFTSLYVAGKMLQGTG-
Thermodesulfovibrio_yellowston  -----------------------------------MPKKFIVANWKMHKTVREALAFLDEFIPITKGL------NGREIGIAPTFICIESVGKVLINTS-
Dehalogenimonas_lykanthroporep  ---------------------------------MSERPTVIAGNWKMHTTVDEAVELVGEMSSALAEF------ENVEVIICPPFIALCKVKELLGQANR
Dehalococcoides_sp              -----------------------------------MRQIIIAGNWKMNTTLSEACTLVQSMKCELERI------EGIEKIICPPFISLYPIKTILENSS-
Geobacter_metallireducens       -----------------------------------MRTPIIAGNWKLFKKSSEAQDFVAELIPLVQKT------TDVEIVIAPVFTVLSAVKRAIADCN-
Desulfobacter_postgatei         ----------------------------------MTRIPLIAGNWKMYKTGTQAVAAAKQLAHLSKGV------DGVDIMIAPTTLSLPLVAAALGKSSP
Planctomyces_brasiliensis       -----------------------------------MRRYLIAGNWKMNTSLETGTALASGLADHVRGRD-----LPVDVLVCPPFPYLAAVKATAGEA--
Spirochaeta_coccoides           -----------------------------------MRRYYIAGNWKMNLTPSEGALYAAELAKAVQKGK-----PDAKVMIAPAFVAIPAVLEAVKGS--
Denitrovibrio_acetiphilus       -----------------------------------MRKPYIYGNWKMNLDMHGSSDLISSILS-VKIDY-----TKVDVGVAPDFSSLYKVSQTIKQMG-
Prosthecochloris_aestuarii      -----------------------------------MRKKIVVGNWKMNKTIAEAVELSSAIIDQLGEVS-----ASCEVGIAPAFPALSGVHGVISGT--
Chlorobium_phaeobacteroides     -----------------------------------MRKKIVVGNWKMNKNVAEGVALASSILENLDGGK-----MACEVGIAPAYPVLSEVGRVIEGS--
Chlorobium_ferrooxidans         -----------------------------------MRQKIVVGNWKMNNTIAESSALASELVAALGKGF-----SGCEVGIAPTYVALQEAGKIIAGS--
Chloroherpeton_thalassium       -----------------------------------MRKKFVAGNWKMNKDLLGAVSLATEILQLLGDEA-----PTCEVAIAPTFLCQQAVFQVIDES--
Treponema_pallidum              -----------------------------------MRGYFIAGNWKMHKTCAEAVALAQELVRELRGG-------PHTYMIAPSFTALDAVGKVLRGS--
Leptospira_interrogans          -----------------------------------MRKTIIAGNWKMNLSLKEAVFLAHSIREKIPSIS-----KDKVSMVFPSTLHLENVSKILEGS--
Borrelia_turicatae              -----------------------------------MRKIFLAGNWKMHYTSMEAAGVAKQIVDGVKNIK-----DDVVVMITPPFTSLCKVCEVTRGS--
Thermotoga_maritima             ----------------------------------ITRKLILAGNWKMHKTISEAKKFVSLLVNELHDV------KEFEIVVCPPFTALSEVGEILSGR--
Fusobacterium_nucleatum         -----------------------------------MRRLVIAGNWKMYKNNKEAVETLTQLKDLTRDV------KNVDIVIGAPFTCLSDAVKIVEGS--
Selenomonas_sputigena           ----------------------------------MARRPIIAGNWKMNNTIAEGVALVKELMPLVKDA------KDVDVVACPTATALAAVAEAVKGS--
Ruminococcus_sp                 ----------------------------------MARKKIIAGNWKMNMTPSEAVKLVETLKPLVVN-------DEVDVVFCVPAIDIIPVVEATKGT--
Clostridium_botulinum           -----------------------------------MRTAIIAGNWKMNKTVKEAVELVKELKPLVKD-------AKCDVVVCPTYVCLPAVLEEVKGS--
Clostridium_perfringens         -----------------------------------MRTPIIAGNWKMHYTIDEAVKLVEELKPLVKD-------AKCEVVVCPTFVCLDAVKKAVEGT--
Thermoanaerobacter_wiegelii     ---------------------------MISKGVFVLRRPIIAGNWKMHMTPSEAVKLVEELIPQVKD-------AKAEVVVIPPFVDLTEVNKVIQGT--
```

```
Brachyspira_murdochii             ----------------------------------MARRKLIAGNWKMNNSNKEALELVNQLKNLVKDV------KDRDIMIAPTFTCLSDVHNAVKGS--
Acholeplasma_laidlawii            ---------------------------------MAKRIPVIAANWKMFKTKDEALEFIFAVNAAVPSRD------EVESIICAPAILLNLLVKREGE---
Lactobacillus_reuteri             -----------------------------------MRKPFIAANWKMHKNVQESVEFVDAIKGKLPDPQ------EVEVGIAAQAFALPSMVQAADDS--
Bacillus_subtilis                 -----------------------------------MRKPIIAGNWKMNKTLGEAVSFVEEVKSSIPAAD------KAEAVVCAPALFLEKLASAVKG---
Bacillus_thuringiensis            -----------------------------------MRKPIIAGNWKMNKTLSEAVSFVEEVKGQIPAAS------AVDAVVCSPALFLERLVAATEG---
Listeria_monocytogenes            -----------------------------------MRKPIIAGNWKMNKTAAKAGQFAEDVKNNVPSSD------AVESVVAAPALFLQELVRLTEG---
Geobacillus_stearothermophilus    -----------------------------------MRKPIIAGNWKMHKTLAEAVQFVEDVKGHVPPAD------EVISVVCAPFLFLDRLVQAADG---
Staphylococcus_aureus             -----------------------------------MRTPIIAGNWKMNKTVQEAKDFVNALP-TLPDSK------EVESVICAPAIQLDALTTAVKEGKA
Enterococcus_faecalis             -----------------------------------MRKPIIAGNWKMNKTLSEAQSFAEAVKNAVPSND------VVDAVIGSPALFLAPLAWNLKD---
Lactococcus_lactis                ----------------------------------MSRKPIIAGNWKMNKTLSEAQAFVEAVKNNLPSSD------NVESVIGAPALFLAPMAYLRQG---
Streptococcus_pneumoniae          ----------------------------------MSRKPFIAGNWKMNKNPEEAKAFVEAVVSKLPSSD------LVEAGIAAPALDLTTVLAVAKG---
Erysipelotrichaceae_bacterium     -----------------------------------MRKPIIVGNWKMNKTMKETKEFMEAVDAAAASEN------AVFG-IGAPYTALSAAVAGAKN---
Lentisphaera_araneosa             ----------------------------------MKRRLFIAGNWKLNKTAAETTETVADLKAKLADFNG-----SLDVALFPPFLSIAAGQAAAAG---
Chlamydophila_pneumoniae          ----------------------------------MTRQSYVLGNWKMHKTIQEAKEYVQTLASLLQG-EP----LSCTIGIASPFTSLRAIHEMINTTG-
Desulfovibrio_vulgaris            ------------------------------------MKKLIAANWKMYKTIDEARATGRELVSAVAGSLP----ADREVLVCPPFTALHALHDTFKDVE-
Mesorhizobium_opportunistum       ------------------------------------MVYWVGTSWKMNKTLAEAMDFAAILAGFVPG-FD----DRIQPFVIPPFTAVRQVKQALSSTR-
Bartonella_henselae               --------------------------------MSPNIRPFIAGNWKMNGTGES-LGELRAIAAGISSDLGRL----FEALICVPATLLSRAFDILGGEN-
Brucella_abortus                  --------------------------------MTPGIRPLVAGNWKMNGKGES-LTELRAIAAGLSSDLGRK----LDAVICVPATLLSRAAETLEGET-
Rhizobium_etli                    --------------------------------MTPDVRPLVAGNWKMNGTRAS-LDQIKAIAEGVRPPLADK----VEALICPPATLLYVATALCTDSP-
Agrobacterium_tumefaciens         --------------------------------MTPNVRPLVAGNWKMNGTRAS-LDQIKAMAEGVKGALSEK----VDALICPPSTLLYVATALCDDSP-
Rhodobacter_sphaeroides           ------------------------------------MRKLAAGNWKMNGTEAD-LGEIDALLA---AHPAPR----CEMLICPPATLVARMAGR-AAQG-
Ruegeria_sp                       -----------------------------------MARKLAAGNWKMNGTGEH-LAELENLAQ---GDLPEG----VDVLICPPATLISRAADR-AGD--
Bradyrhizobium_japonicum          --------------------------------MTDAIRPLIAGNWKMNGLKAS-AAEFDAMLNGA-ADVATK----ADLLVCPPATLIAAFADKARGKK-
Rhodopseudomonas_palustris        -------------------------------MPVPAVRSLIAGNWKMNGLKAA-SSEFDAMLEGA-TELSSG----IDLLVCPPATLIAAFADRLAANEK
Methylobacterium_radiotolerans    -------------------------------MTQSGRRPLVAGNWKMNGTRSS-IQVVEAIRDGLSPDLASR----IDVLICPPATLIGSCVAAAAGSP-
Rhodospirillum_rubrum             ---------------------------------MTVRRPLIAGNWKMNGLKAEGQGLARDLAQRLNKAGGVD----FDLLLCPPFPLISTVVGAVAGSG-
Zymomonas_mobilis                 ----------------------------------MTIRKLIAGNWKMNG-SLGMLHELDGIAKAT-FNHPE-----LDVVICPSFTLIAPA--IVRQPD-
Gluconacetobacter_hansenii        ------------------------------------MKQIIVGNWKMHGMTADAAKAADDIGAAMGADVSG-----AQVVICPPFTQLALLGPKLREHG-
Thermus_thermophilus              -----------------------------------MRRVLVAGNWKMHKTPSEARVWFAELKRLLPPLQS-------EAAVLPAFPILPVAKEVLAE---
Thermus_aquaticus                 -----------------------------------MRRVLVAGNWKMHKVPSEARVWLAELKRLLPPLDS-------EAAVLPAFPMLPVAKEVLSG---
Oceanithermus_profundus           -----------------------------------MRTPLLAGNWKMHKTPSEARVWFAEFLEANPTLQSGG-QAAVEVALLPSFPLVPVAAEQLAG---
Deinococcus_deserti               -----------------------------------MPNNLLALNWKMNKTPSEAQAWAAELSSALSPGEA-------ELAVMAPAIMLPALAAGLP----
Bifidobacterium_bifidum           --------------------------------MAS-RIPLLAGNWKMNLDHLEATYFVQKLVWLLRDARFDY--SRCEIALMPSFTSLRSVQVLVEADK-
Bifidobacterium_longum            --------------------------------MASKRIPLVAGNWKMNFDHLEATYFVQKLVWLLRDAHFDF--KRCEVALFPSFTSLRSVQVLVEADK-
Gardnerella_vaginalis             --------------------------------MVVNRKPLVAGNWKMNFNHLEATHFIQKFWWRLCDSHFDC--KKCDIALMPSFTSLRSVQVLVESDN-
Nocardia_farcinica                ----------------------------------MARKPLIAGNWKMNLNHLEAIALVQKIAFALPEKYFE----KVDVAVIPPFVDIRSVQTLVEGDK-
Mycobacterium_tuberculosis        ----------------------------------MSRKPLIAGNWKMNLNHYEAIALVQKIAFSLPDKYYD----RVDVAVIPPFTDLRSVQTLVDGDK-
Corynebacterium_glutamicum        ----------------------------------MARKPLIAGNWKMNLDHQQAIGTVQKLAFALPKEYFE----KVDVAVTVPFTDIRSVQTLVEGDK-
Propionibacterium_acnes           ----------------------------------MSRTPIMAGNWKMNLDHVAATSLVQDLGEALKAAGYDS--SKSEAVVIPPFTDIRPVAIIIDGDK-
Actinomyces_odontolyticus         ----------------------------------------MAGNWKMNLDHLEANHLVQGLAMALSDAGHDY--SKCEVLVIPPFTDIRTVQTIVDADE-
Microbacterium_testaceum          --------------------------------MGVTRTPLIAGNWKMNLDHLQAVAFVQKLHWTLKEAKHEN--GSVEVAVFPPFTDLRTVQTLLDADK-
Moritella_marina                  -----------------------------------MRHPVVMGNWKLNGSKEM----VVDLLNGLNAELEG--VTGVDVAVAPPALFVDLAERTLTEA--
Photobacterium_profundum          -----------------------------------MRHPVVMGNWKLNGSKEM----VVNLLNGLNAELEG--VTGVDVAVAPPALFIDLAEHTLTEA--
Vibrio_cholerae                   ---------------------------MIRYGDIPMRRPVVMGNWKLNGSKAM----VTDLLNGLNAELEG--VEGVDVVVAPPAMYLDLAERLIKEG--
Shigella_flexneri                 -----------------------------------MRHPLVMGNWKLNGSRHM----VHELVSNLRKELAG--VAGCAVAIAPPEMYIDMAKREAEG---
Escherichia_coli                  -----------------------------------MRHPLVMGNWKLNGSRHM----VHELVSNLRKELAG--VAGCAVAIAPPEMYIDMAKREAEG---
```

```
Salmonella_enterica               -----------------------------------MRHPLVMGNWKLNGSRHM----VNELVANLRKELTG--VAGCDVAIAPPEMYIDLAKRAAAG---
Klebsiella_pneumoniae             -----------------------------------MRHPLVMGNWKLNGSRHM----VNELVANLRTELAG--VSGCAVAIAPPEMYLDLAKRAAEG---
Enterobacter_aerogenes            -----------------------------------MRHPLVMGNWKLNGSRHM----VNELVANLRTELAG--VSGCAVAIAPPEMYLDLAKRAAEG---
Serratia_symbiotica               -----------------------------------MRHPLVMGNWKLKGSTHM----VSELIAGLRNALSR--VDGCGVAIAPPVMYLDQARHALAG---
Yersinia_pestis                   -----------------------------------MRHPLVMGNWKLNGSTHM----VNELIAGLRKELST--VDGCGVAIAPPAIYLNQAKHELAG---
Actinobacillus_pleuropneumonia    ----------------------------------MARRPLVMGNWKLNGSKAF----TKELIAGLKAELAD--VKGCDVAIAPPVMYLAEAEAALAGQ--
Pseudomonas_aeruginosa            -----------------------------------MRRPLVAGNWKMHGTHSS----VAELIKGLR-QLAL--PSGVDVAVMPPCLFISQVIQGLAG---
Marinomonas_posidonica            ----------------------------------MIRQKIVAGNWKMNGSKAS----ISSLVNGLLEMSE----SSAEVVVTPPFPYLSQVADVIAQQ--
Xylella_fastidiosa                -----------------------------------MRPKIVAGNWKLHGSHAF----AQALVAQVAAGLP---LLGVSVIILPPLLYLSDLAQRFKG---
Xanthomonas_campestris            -----------------------------------MRRKIVAGNWKLHGTRAF----ATELVAQVAAHMP---LAGVDVVILPPLPYLGDLIEDFEA---
Marinobacter_algicola             -----------------------------------MRRKIVAGNWKMNGSKGL----VSNLVGQVRSELASF-DNGVEAVIIPPALFVDDVVEQA-G---
Aeromonas_caviae                  ---------------------------------MGHRKPFVAANWKLHGSRSQ----LEQFTSQCAERI----DEDVDVVLCPSHVHLGFTAELLGEQG-
Pseudoalteromonas_haloplanktis    ---------------------------------MAARKAMVAGNWKMNGSLEL----VKQMSDAINNVK----SNEIDIVLFP---PFPLVSAMIASG--
Legionella_pneumophila            -----------------------------------MRQKIVAGNWKMNGQIQQ----VTELVSQIEELIGF--DCAAQVAVMPPSIYIPKVRDCLRTG--
Ralstonia_solanacearum            -------------------------------MS--ARPKLVVGNWKLHGSLNG----NAELLEKIKAAG----QARAALAVCAPFPYLAQCQSLLAGS--
Burkholderia_sp                   -------------------------------MSNQQRAKLVVGNWKMHGRLAA----NAALLQAVVQGANEL-PADVRVGVCVPSLYLAQTQSLLEGT--
Bordetella_pertussis              ------------------------------MTTAENRARLVLGNWKMHGNLAE----NAALLAELRAAD--A-AAHCEMGVCVPFPYLAQTAAALQGS--
Nitrosococcus_watsonii            -----------------------------------MRTSLVVGNWKMNGSRAA----NRALLESMRKEM-EA-GVVAEVAVCPPFVYLADMESLLQGS--
Coxiella_burnetii                 ----------------------------------MQRRPLVAGNWKMHGSRES----VGQLLRALKHGC-ER-LETAELAVFPPFVFLQQCEEALMRT--
Variovorax_paradoxus              -------------------------MVTTTNNAMTTKKKLIAGNWKMNGGLAV----NEALVKALQQGLAAS-PAACDVALCAPAPYFAQLQSLLAGTP-
Halomonas_elongata                -----------------------------------MPTPLIAGNWKMNGSLDL----IESFGRALADAE--L-PSRLEVALMVPFPYLEAASRALTGS--
Tenebrio_Molitor                  ----------------------------------MARKFVVGGNWKMNGDKKQINEIIG-FLKSGPLNQD------TEVVVGVPAIYLELVRTCVP----
Bombyx_mori                       ----------------------------------MGRKFVVGGNWKMNGDKNQINEIVN-NLKKGPLDPN------VEVIVGVPAIYLSYVKTIIP----
Apis_mellifera                    ----------------------------------MGRKFFVGGNWKMNGTKSEINDIVG-FLKKGPLDSN------VEVVVGVPSIYLTYAKNILP----
Aedes_aegypti                     ----------------------------------MGRKFCVGGNWKMNGDKASITDLCK-TLTTGPLSAD------TEVIVGCPAPYLTLARSQLP----
Anopheles_gambiae                 ----------------------------------MGRKFCVGGNWKMNGDKASITELCK-TLSAGPLDPN------TEVVVGCPAPYLSLARSLLP----
Drosophila_melanogaster           ----------------------------------MSRKFCVGGNWKMNGDQKSIAEIAK-TLSSAALDPN------TEVVIGCPAIYLMYARNLLP----
Rhipicephalus_microplus           ---------------------------------MAARRFCVGGNWKMHGSKNSIRDICN-TLKGASLDPN------VEVIVACPAPYLDYCRSLLP----
Ixodes_scapularis                 ---------------------------------MSGRKFCVGGNWKMNGNKSSIKEICD-MLKTAKLDPN------TEVVLGCPAPYLDYVRRILP----
Anolis_carolinensis               --------------------------------MAP-RKFFVGGNWKMNGDKKSLGELIQ-TLNGAKVSGD------VEVVCGAPTIYLDFARQKLD----
Gallus_gallus                     --------------------------------MAP-RKFFVGGNWKMNGDKKSLGELIH-TLNGAKLSAD------TEVVCGAPSIYLDFARQKLD----
Orcinus_orca                      --------------------------------MAPSRKFFVGGNWKMNGRKNNLGELIN-TLNAAKVPAD------TEVVCAPPTAYIDFARQKLD----
Bos_taurus                        --------------------------------MAPSRKFFVGGNWKMNGRKNNLGELIN-TLNAAKVPAD------TEVVCAPPTAYIDFARQKLD----
Canis_lupusfamiliaris             --------------------------------MAPSRKFFVGGNWKMNGRKKNLGELIT-TLNAAKVPAD------TEVVCAPPTAYIDFARQKLD----
Odobenus_rosmarus                 --------------------------------MAPSRKFFVGGNWKMNGRKKNLGELIT-TLNAAKVPAD------TEVVCAPPTAYIDFARQKLD----
Oryctolagus_cuniculus             ---------------------------------APSRKFFVGGNWKMNGRKKNLGELIT-TLNAAKVPAD------TEVVCAPPTAYIDFARQKLD----
Nomascus_leucogenys               --------------------------------MAPSRKFFVGGNWKMNGRKQSLGELIG-TLNAAKVPAD------TEVVCAPPTAYIDFARQKLD----
Pan_troglodytes                   --------------------------------MAPSRKFFVGGNWKMNGRKQSLGELIG-TLNAAKVPAD------TEVVCAPPTAYIDFARQKLD----
Homo_sapiens                      --------------------------------MAPSRKFFVGGNWKMNGRKQSLGELIG-TLNAAKVPAD------TEVVCAPPTAYIDFARQKLD----
Pongo_abelii                      --------------------------------MAPSRKFFVGGNWKMNGRKQSLGELIG-TLNAAKVPAD------TEVVCAPPTAYIDFARQKLD----
Macaca_mulatta                    --------------------------------MAPSRKFFVGGNWKMNGRKQNLGELIG-TLNAAKVPAD------TEVVCAPPTAYIDFARQKLD----
Ochotona_princeps                 ---------------------------------APSRKFFVGGNWKMNGRKKNLGELIT-TLNAAKVPAD------TEVVCAAPTAYIDFARQKLD----
Sus_scrofa                        --------------------------------MAPARKFFVGGNWKMNGRKNNLGELIN-TLNAAKLPAD------TEVVCAPPTAYIDFARQKLD----
Mus_musculus                      --------------------------------MAPTRKFFVGGNWKMNGRKKCLGELIC-TLNAANVPAG------TEVVCAPPTAYIDFARQKLD----
Rattus_norvegicus                 --------------------------------MAPSRKFFVGGNWKMNGRKKCLGELIC-TLNAAKLPAD------TEVVCAPPTAYIDFARQKLD----
Xenopus_laevis                    ---------------------------------MSPRKFFVGGNWKMNGDKKSLGELIN-TLNSGKMNAD------TEVVCGAPAIYLDFARQKLD----
```

```
Xenopus_tropicalis                 ---------------------------------MSSRKFFVGGNWKMNGDKKSLTELIN-TLNSGKISAD------TEVVCGAPTIYLDFARQKLD----
Danio_rerio                        ---------------------------------MSGRKFFVGGNWKMNGDKKSIEELAN-TLNSAKLNPD------TEVVCGAPTIYLDYARSKLN----
Ictalurus_punctatus                ---------------------------------MTARKFFVGGNWKMNGDKKSLGELIN-TLNGAKLNAD------TDVVCGAPSIYLDFARSKLD----
Oreochromis_niloticus              ---------------------------------MT-RKFFVGGNWKMNGDKKSLGELIQ-TMNGAKVDPN------VEVVCGAPSIYLDFVRSKLD----
Nematostella_vectensis             --------------------------------MG--RKFFVGGNWKMNGSLVQIDGILK-NLHDSSLSDD------TEIVVSPPALYLSYVRKAAK----
Schistosoma_mansoni                --------------------------------MSGSRKFFVGGNWKMNGSRDDNDKLLK-LLSEAHFDDN------TEVLIAPPSVFLHEIRKSLK----
Strongylocentrotus_purpuratus      --------------------------------MASGRRFWVGGNYKMNGSKASIDGLLK-MLEESQIPGN------TDVVVAPPTVYLDYMNSKIP----
Caenorhabditis_elegans             ----------------------------------MTRKFFVGGNWKMNGDYASVDGIVT-FLNASADNSS------VDVVVAPPAPYLAYAKSKLK----
Caenorhabditis_remanei             ----------------------------------MTRKFFVGGNWKMNGDFASVDGIVT-FLNQSADNAS------VDVVVAPPAPYLAYAKSHLK----
Brugia_malayi                      ----------------------------------MSRKFLVGGNWKMNGNKASVDNIIK-FLNDGAVVPN------VDVVVAPPAPYLSYVKEKVK----
Trichinella_spiralis               ---------------------------------MSSRKFFVGGNWKMNGDSKSIEEYIK-LLNDNTLPGN------VEIVIAPPFVYLERIRKQLK----
Ricinus_communis                   ----------------------------------MARKFFVGGNWKCNGTSEEVKKIVS-TLNEGHVPSSD----VVEVVISPPFVFLPLVKDSLK----
Vitis_vinifera                     ----------------------------------MGRKFFVGGNWKCNGTGEEVKKIVS-TLNAGEVPSGD----VVEVVVSPPFVFLPLVKSTLR----
Arabidopsis_thaliana               ----------------------------------MARKFFVGGNWKCNGTAEEVKKIVN-TLNEAQVPSQD----VVEVVVSPPYVFLPLVKSTLR----
Zea_mays                           ----------------------------------MGRKFFVGGNWKCNGTTDQVEKIVK-TLNEGQVPPSD----VVEVVVSPPYVFLPVVKSQLR----
Oryza_sativa                       ----------------------------------MGRKFFVGGNWKCNGTTDQVDKIVK-ILNEGQIASTD----VVEVVVSPPYVFLPVVKSQLR----
Fragaria_vesca                     ----------------------------------MGRKFFVGGNWKCNGTIDEVKKIVN-LLNDGKVPSPD----CVEVVVSPPYVFLPLVKSTLR----
Glycine_max                        ----------------------------------MGRKFFVGGNWKCNGTTEEVKKIVT-TLNEAKVPGED----VVEVVVSPPFVFLPFVKSLLR----
Cucumis_sativus                    ----------------------------------MARKFFVGGNWKCNGTNEEVKKIVS-TLNEAEIPSED----AVEVVVSPPFVFLSLVKSLLR----
Perkinsus_marinus                  ----------------------------------MSPKPFVGGNWKSNGSVESIKKLAP-ALVAGEATYNKD---AVDVVIAPTAIHLPLAMEQFKG---
Aspergillus_niger                  ----------------------------------MPRQFFVGGNFKMNGTADSITSIIK-NLNAAKLDE------SAEVVVSPPTLYLLPARQAAG----
Penicillium_chrysogenum            ----------------------------------MPRQFFVGGNFKMNGVAQSITDIVN-NLNAAKLDD------TTEVVISPPAIYLALTRELAN----
Paracoccidioides_brasiliensis      ----------------------------------MPRKFFVGGNFKMNGTAKSITHIIT-NLNSAKLDP------STEIVIAPPAIYLVLARQLAN----
Schizosaccharomyces_pombe          ----------------------------------MARKFFVGGNFKMNGSLESMKTIIE-GLNTTKLNVG-----DVETVIFPQNMYLITTRQQVK----
Saccharomyces_cerevisiae           ----------------------------------MARTFFVGGNFKLNGSKQSIKEIVE-RLNTASIPE------NVEVVICPPATYLDYSVSLVKK---
Kluyveromyces_lactis               ----------------------------------MARTFFIGGNFKMNGSKASIKEIVD-RLNGASIPS------NVEVVIAPPAIYLDHAVALNKR---
Candida_dubliniensis               ----------------------------------MARQFFVGGNFKANGTKQQITSIID-NLNKADLPK------DVEVVICPPALYVGLAVEQNKQ---
Clavispora_lusitaniae              ----------------------------------MARQFFVGGNFKMNGTKESITKIID-NLNKADLPK------DVQVVIAPPAPYLSLAVAENKQ---
Chlamydomonas_reinhardtii          MQLCKVQRASAARSSRASRS------QRVEVVCASSAKFFVGGNWKCNGSVANVAKLVD-ELNAGTIPRG------VDVVVAPPFIYIDYVMQHLDR---
Volvox_carteri                     MLATRSLRASGCRSPAVTQPRLALGRGRLEIVCASNAKFFVGGNWKCNGSVASVRKLVD-ELNSGSVPRG------VDIVCAPPFIYIDYVMQHLDK---
Dictyostelium_fasciculatum         --------------------------------MTGSRKFFVGGNFKCNGNKSSLQTLIE-SIIKSTLPASE----IVDIVVSPPSIYLEYVNQLVG----
Entamoeba_histolytica              ---------------------------------MSARKFVVGGNWKCNGTLASIETLTK-GVAASVDAELAK---KVEVIVGVPFIYIPKVQQILAGEAN
Cryptosporidium_parvum             ----------------------------------MSRKYFVGGNFKCNGTKESLKTLID-SFKQVE--SSN-----SEVYVFPTSLHISLVKEFFGNDHP
Cryptosporidium_muris              ----------------------------------MARQPFVGGNFKCNGTKDSLRLLLK-SFKDSQI-TTN-----TQVYVFPTFIHIPLAYEILEDSG-
Plasmodium_falciparum              ----------------------------------MARKYFVAANWKCNGTLESIKSLTN-SFNNLDFDPSK-----LDVVVFPVSVHYDHTRKLLQSKF-
Plasmodium_vivax                   ----------------------------------MTRKYFVSANWKCNGTQESVKALAA-SFNELDFDPAK-----LDVVVFPVSVHYELAKSLLKPKF-
Paramecium_tetraurelia             -----------------------------------MRRYFVGGNWKCNNTIAQTQSLINTVINKLVFDVNK-----VEVAVAPIFLHVPWVQANIQKNV-
Tetrahymena_thermophila            ---------------------------------MAIRRLFVGGNWKCNNTLAQTKDLVNNVLNKLHFDPAK-----VDVVVAPVFLHVPWVAEHIQKPV-
Toxoplasma_gondii                  ----------------------------------MVRTPWVGGNWKCNGTVGSITDLCG-EFGKTEFDPKT-----IDVVIFPPALHAPLTREKLPKKY-
Trypanosoma_brucei                 ---------------------------------MSKPQPIAAANWKCNGSQQSLSELID-LFNSTSI-NHD-----VQCVVASTFVHLAMTKERLSHPK-
Trypanosoma_cruzi                  --------------------------------MASKPQPIAAANWKCNGSESLLVPLIE-TLNAATF-DHD-----VQCVVAPTFLHIPMTKARLTNPK-
Leishmania_mexicana                --------------------------------MSAKPQPIAAANWKCNGTTASIEKLVQ-VFNEHTI-SHD-----VQCVVAPTFVHIPLVQAKLRNPK-
Leishmania_donovani                --------------------------------MSSKPQPIAAANWKCNGTTASIEKLVQ-VLNEHNI-SHD-----VQCVVAPTFVHIPLVQAKLRNPK-
Giardia_lamblia                    --------------------------------MPAR-RPFIGGNFKCNGSLDFIKSHVA-AIAAHKI-PDS-----VDVVIAPSAVHLSTAIAANTSKQ-
Trichomonas_vaginalis              -----------------------------------MRTFFVGGNWKANPKTVEEAEKLIEMLNGAKVEGN------VEVVVAAPFIFLPTLQQKLR----
Sphingobacterium_sp                -----------------------------------MRKKIVAGNWKMNMDYQEGISLFSEVANMVKDEVIG----NQQIIVCSPFIHLHSIASLAKESE-
```

```
Pedobacter_saltans               -----------------------------------MRKKIVAGNWKMNKDYSEGLSLFSEVLNMVKDEVRG----EQQVVVCAPYIHLHSLSALAKGTN-
Marivirga_tractuosa              -----------------------------------MRQKIVAGNWKMNKTLVEAQSLTSEIANMVEDEAPS----DVKVVIAPPAPFIYAVENLIPEMG-
Cyclobacterium_marinum           -----------------------------------MRKKIVAGNWKMNCLLEEGQKLTSEIVNMIKDEPIK----DVKVILNPPFVHLHGVKKLIAGVD-
Leadbetterella_byssophila        -----------------------------------MRKKYAAGNWKMNKTFEEGQILLSEVVNMVKDELTHP---NVQVVLGVPFPYLSTFSKLVDTP--
Haliscomenobacter_hydrossis      ---------------------------------MKTRQQIVAGNWKMNKNYGEGRELAMEIVERLK---PS----NTQVVLCAPYIHLQLVKNIIKDVA-
Parabacteroides_distasonis       -----------------------------------MRKNIVAGNWKMNTTLAEGLALAKGLDEALKGKTPN-----CDVIIGTPFTHLASVAAAIDTN--
Bacteroides_fragilis             -----------------------------------MRKNIVAGNWKMNKTLQEGIALAKELNEALANEKPN-----CDVIICTPFIHLASVTPLVDAA--
Prevotella_ruminicola            -----------------------------------MRKKIVAGNWKMNMNLQDGIALAKELNETLKADKPN-----CGVVICTPFIHLASIAQFLDQD--
Alistipes_shahii                 -----------------------------------MRKKIVAGNWKMNTLPAEGVELAKGVVAGRGEVCSC-----VNFIVCPPFTHLAMVAEALKGS--
Porphyromonas_asaccharolytica    ---------------------------------MDKRTLIVAGNWKMNMTLDAGLRLITDIKEQVAKLSNP-----CKVILAPPYIHLGAMKGLLAGS--
Cellulophaga_lytica              -----------------------------------MRAKIVAGNWKMNKTLTETNALLAELSGKLP----DT---DAEVMVAPTYVNLAAAVEDVKSS--
Kordia_algicida                  -----------------------------------MRRKIVAGNWKMNNDLSETEALLNDLIEQVP----NT---DASVMVAPAFVNLFQAFNVLKTF--
Flavobacterium_psychrophilum     -----------------------------------MRAKIVAGNWKMNKNSEETEDLINELIDKLPT---NS---NAKIIIAPTFINLASAVDHTEFT--
Neisseria_gonorrhoeae            ----------------------------MYRQIGMWDQKWVIGNWKMNGRLQNNNALMHRF-RILPTAER------VLIGLAAPTVYLLQLHNAMQIVLN
Francisella_tularensis           ------------------------------------MQKLIMGNWKMNGNSTSIKELCSGISQVQYDTSR------VAIAVFPSSVYVKEVISQLPEKVG
Mycoplasma_genitalium            -----------------------------------MRTRYLIGNWKTNKNLKDAVSFVEQFQQNKLNYN-------AKIGIAPVYVHLTEIKKIISDS--
Ureaplasma_urealyticum           ----------------------------------MVKMKYIIANFKMNATEELINHFLNNLISFDE---------QKLTIGLAPGDLYLKTFVDLSQTK-
Ureaplasma_parvum                -------------------------------------MKYIIANFKMNATQELINHFLNNLTLFDE---------QKIIIGLAPGDLYLKTFVNLAEIK-
Buchnera_aphidicola              -----------------------------------MKKFFITANWKLNGNIKMISSFFKYLKLYSSSYL------EKNTVIIAPPTIYLERVCKNISNM-
Campylobacter_jejuni             --------------------------------------MIFAANLKCNHTRASFKIYAEILNKTIG-VK------CDDIIVFPP-----SVAFLEKEN--
Helicobacter_pylori              ------------------------------------MTKIAMANFKSAMPIFKSHAYLKELEKTLKPQH------FDMVFVFPD-----FLGLLPNSFL-
Orientia_tsutsugamushi           ------------------------------------MESIVVSNWKMHFSFSEACNYLNLITSLNSNLN------LAKMIFAVPNLYLSGLKLKFNDTY-
Wolbachia_sp                     ------------------------------------MSFLIVANWKMNGMRSSFVDFIGKLNNKSNEIT------SKLVICPPFT----SFPSSIELNN-
Methanocaldococcus_Jannaschii    --------------------------------------MVIVINYKTY-NESIGNRGLEIAKIAEKVSEESG----ITIGVAPQFVDLRMIVENVNIP--
Methanococcus_voltae             -----------------------------------MIKPVVIINYKTY-MESISDKGLNIAKCAEKVSEESG----IEISVAPQFTDLKTIVEQTNVK--
Methanothermococcus_okinawensi   -----------------------------------MIKPIIIINYKTY-AESVGEKGLKIAKAAEKVSEESG----ISIGVCPQFLDLRMISENVNIP--
Pyrococcus_Woesei                --------------------------------MAKLKEPIIAINFKTY-IEATGKRALEIAKAAEKVYKETG----VTIVVAPQLVDLRMIAESVEIP--
Pyrococcus_furiosus              --------------------------------MAKLKEPIIAINFKTY-IEATGKRALEIAKAAEKVYKETG----VTIVVAPQLVDLRMIAESVEIP--
Thermofilum_pendens              ---------------------------------MKIGYPLILINFKAY-SEASGKRGLQLAKVAEKVSKETG----ITIAVAPQLTDLAFIASQVEIP--
Methanothermobacter_thermautot   ----------------------------MLEDLELKDTPIVILNFKTY-LESTGERALELASICGDVADETG----VNMAVAPQHMDLHRVSDAVEIP--
Methanosaeta_thermophila         --------------------------MRWSQSLGGMALTLIVLNFKTY-REATGDSAVALSKICESIASEYG----VDIAVAPQAADIRAVASAVSIP--
Natronobacterium_gregoryi        --------------------------------------MFVLVNLKTY-PCDP----LEIATAVRDVDETTD----ARLAVAPQAIHIERVAET-GVE--
Halobacterium_salinarum          --------------------------------------MFVLVNLKAY-PCDP----VAIAEAAADVAETTP----ATIAVAPQPADIGRVADT-GAT--
Halogeometricum_borinquense      --------------------------------------MFILVNLKAY-PCDP----LEVATAAHEVAEESG----VRIAVAPQAADVRRVADT-GVE--
Methanosphaerula_palustris       -----------------------------------MSSQFILVNLKAY-KEGMGPRAHMIARAAEEVSEESG----IQIGVAPSFFDLHPLSKHFEIP--
Thermoproteus_tenax              -----------------------------------MRLPILIINFKAY-GEAAGKRAVELAKAAERAARELG----VNIVVAPNHLELGLVSQSVDIP--
Pyrobaculum_islandicum           -----------------------------------MKFPILIINLKAY-GEAAGKKALEIAKAAEKVAKELG----VNIAVAPNHLELALVAQSVEIP--
Cenarchaeum_symbiosum            ---------------------------------------MLIINCKNY-KEAAGGRIDSLAAAAAGAAAKYG----VRIALAPPQHLLGAVKGEDLT---
Nitrosopumilus_maritimus         ---------------------------------------MFVINCKNY-EEISGEKITKFVKTAEKVSKKFK----VKIAICPPQHLIGVVANSSIP---
Desulfurococcus_kamchatkensis    ----------------------------------MVKP-VLAVNFKAYYPHSFGENAYRLAKDAVRVWRETG----VEVILAPPFTELKSVIEAVKDTG-
Pyrolobus_fumarii                ----------------------------------MRDVYILAVNFKVY-PSAFGRRALEVVKAAERVAREFEG--TVSVIIAPPHTEIARISSSVER---
Acidilobus_saccharovorans        ----------------------------------MPRL-VFAVNFKAY-ETAFNEKSLEIAREASKASSRYG---NVRVILIVPAAIASKVLQIYDD---
Sulfolobus_islandicus            ----------------------------------MKPP-IIVINFKAY-ENSFGNKAIELGKKIEKISKEYS----VEIILSVPATMIYRMVQEVDLP--
Clustal Consensus                                                           . *
```

```
                                         110       120       130       140       150       160       170       180       190       200
                                    ....|....|....|....|....|....|....|....|....|....|....|....|....|....|....|....|....|....|....|....|
Roseiflexus_castenholzii            --TPIALGAQDVYIE-PQGAFTGAISPVMLRDLGCAYVIVGHSERRAIFGEGDELIGKKVRAAL-AHDLT-PILCVGETKPQRDAGHAETVVVAQVRAAL
Chloroflexus_aurantiacus            --SPLGLGAQNLYPE-AQGAFTGEVSPPMLVDIGCRYVIIGHSERRQYFGESDAFVNRKLRAAL-AHGLR-PIVCVGESKPQRDAGQAEPIVTAQVRAAL
Nostoc_punctiforme                  --SLIQLGAQNIHWE-EFGAYTGEISGPMLTESGVRFVIVGHSERRQYFGETDATVNLRLRTAQ-RFGLT-PILCVGETKQQRDAGETESLIALQLDKGL
Anabaena_variabilis                 --SLVQLGAQNVHWA-ENGAYTGEISGPMLTEIGVRYVIVGHSERRQFFGETDETVNLRLQAAQ-KYGLT-PILCVGETKQQRDSGETESLIVSQLDKDL
Synechocystis_sp                    --GRVRLGAQNVHWE-ASGAYTGEISAAMLTEIGIHYVVIGHSERRQYFGETDETANLRVLAAQ-KAGLI-PILCVGESKAQRDAGETEQVIVDQVKKGL
Cyanobacterium_aponinum             --SRVKLGAQNIHWA-DQGAFTGEISGEMLKEFGINYVIVGHSERRQYFGETDETVNARLLAAQ-KHGLT-PILCVGESKAQRDAGETEAVISAQIEKDL
Crocosphaera_watsonii               --GKVRLGAQNVHWE-DKGAYTGEISGPMLTELSVDYVVIGHSERRQYFSETDETANLRVITAQ-RHGLT-PILCVGESKQQRDAGEAESVIINQLQKGL
Trichodesmium_erythraeum            --SLIRLGAQNVHWE-DEGAYTGEISGLMLKDVGVSYVIVGHSERRQYFGETDETVNMRLKAAQ-KHGLT-PILCVGETKQQRDSGETESHIFSQLANDL
Synechococcus_sp                    --SRVQVGAQNVHWE-REGAYTGEVSGVMLKELGVRYVVVGHSERRQYFGETDETVNARLKAAQ-LHGLT-PILCVGETKQQRDADQTESVITGQIKKAL
Prochlorococcus_marinus             --SNVSLSSQNVHWE-DNGAFTAEISPKMLLEHSVSYAIVGHSEPRKYFSESDKQINLRAKSAQ-ANGLI-PIVCVGETIEQRERGEAERVIRRQVEQGL
Sphaerobacter_thermophilus          --SDIVLGAQDCAVE-DQGAFTGEVSARMLAPL-CRYIIVGHSERRHIIGETDEVVAAKLRAVL-RNGAR-PILCVGELLEERERGEARAVVERQLQSAF
Anaerolinea_thermophila             --TNIGMGAQNMHWE-ASGAFTGEVSPKMVAEF-CQYVILGHSERRTYFGETDETVNRKVKSAL-STGLT-PIVCVGETLAENEAGKTAEVVWRQITEGL
Myxococcus_xanthus                  --APLALAAQNCHWE-SSGAFTGEISAPMLAELGCAYVIVGHSERRQLFGDTDEQVNKRAKAVR-AAGMT-PIICVGETLAEREANQTLAVVERQVRGAL
Collinsella_tanakaei                --SAIKLGAQNVYWE-AAGAYTGESSCDMLKSAGCTYCIIGHSERRDYFHETDEDQNKKAKALV-AAGLV-PVFCCGEPLEVREAGTYVEHVVAQVKAGL
Atopobium_vaginae                   --SSIALGAQNVYWE-ENGAFTGETAPNMLVSLGATYCIIGHSERRGYFGETDEDINKKAKALL-AHNIV-PISCCGESLEVREAGKHVEFVVEQIKADT
Eggerthella_sp                      --TRIAIGAQDVYWE-PKGAFTGEISVPMIKEIGCTYCIVGHSERRELFGETNEDVNRKAKALI-AGGLA-PIVCVGESLAVRDEGTTNEYVCAQVRAAF
Dictyoglomus_thermophilum           ----IKLGAQNMFWE-KEGAYTGEISPIMLKDLNCTYVIIGHSERRNYFSETNEMINKKIKSAF-NYGLI-PIFCVGEKWEERERGKTEEVITKQVREGL
Aquifex_aeolicus                    ----VKLGAQNCHYE-KRGAFTGEISIPMLQEVGCEYVIVGHSERRHIFGESDELIHKKIVACL-EMGIR-PILCVGEKKEEREAGMTFKVIETQIKLAL
Thermodesulfovibrio_yellowston      ----IKLCAQNAFYE-NKGAYTGEVSPAMLKDCGVEYVIIGHSERRKYFYENDDIINKKIHACI-KEGLK-VIFCIGETFEDRQNNKTMEILKTQIRNGL
Dehalogenimonas_lykanthroporep      ----IQLGAQNMYQE-AKGAFTGEISPVMLQDF-CKYVIIGHSERREIFQETDDMIKLKVRAAL-KFGLK-PILCVGENLAQREGGKVQEVIEGQITKAL
Dehalococcoides_sp                  ----IKLGAQNLFYQ-EKGAYTGEISPLMLKEL-CQYVIIGHSERRAYFGETGQVVNQKIKAAL-QAGLL-PIVCVGEKPEENENGQTRQVLETQLKEAL
Geobacter_metallireducens           ----IMLSAQDCFWE-EEGAFTGEISPGMLVDAGCSHVIIGHSERRQYFGETDETVNRKIKAAI-TAGLT-VLFCIGETLAEREADKTFEVLRTQIENGL
Desulfobacter_postgatei             ----IRLGAQNIYPG-TEGAFTGEVSGPMIKDAGADYVIIGHSERRQYFGETDESVRLKIRSAL-DAGLI-PVMCIGETESQRDADETFFILDKQITDGL
Planctomyces_brasiliensis           ---GISVGAQNCYFE-ASGAFTGEVSVDMLKDIGCDSVILGHSERRHVIKECDDMINKKTKAAI-EGGLQ-VVLCVGELLEEREADKTEAVLDEQMAGGL
Spirochaeta_coccoides               ---PIIVAAQNAADH-LSGAYTGEVSLSMLKDLGVGTVILGHSERRAIYGESDALINAKTKLAL-SEGFE-VILCVGETLEEREAGKLEDVLSRQVKIGL
Denitrovibrio_acetiphilus           --YPISVAAQNISAE-EKGAFTGDVSAAMVFAAGADSTILGHSERRMIFAEGDELINQKVKTAL-RNNLD-VILCVGETLDERESGVAADRVVYQVGMGL
Prosthecochloris_aestuarii          ---GIHLAAQNCHYE-DDGAFTGEVSVRMLDEAGCSYVIVGHSERRQYFGDTNPVVNLKVKKAL-SAGLN-VILCVGETLDEREKGITTEVVTCQVKEGL
Chlorobium_phaeobacteroides         ---DICLVAQNCHYE-DEGAYTGEVSVRMLDSLGCSYIIVGHSERRQYFGETNRTVNLRLKKAL-EGGMR-VILCIGETLEEREAGVTDAIVTAQVREGL
Chlorobium_ferrooxidans             ---AVQLVAQNCHYE-NDGAFTGEISTGMLKAVGCSYVIIGHSERRQFFGETNATVNLRIKKAL-AEGLK-VILCVGETLAERESEVTSKVVTSQVTEGL
Chloroherpeton_thalassium           ---AIKLAAQNCFYE-DQGAYTGEISAAMLRNSGCEYVILGHSERRQYFNETDEIVNKKVKNAL-SVELD-VIMCVGETLEQRESGVTKSVVETQVRGGL
Treponema_pallidum                  ---NVLLGAQDVSSE-EWGAHTGEVSVLQLEDLGVQVVIVGHSERRHGRGENDKLINQKVRRVL-ESGLR-VILCVGERLQEYEAGCTNEVVGTQVRAGM
Leptospira_interrogans              ---SVIVGAQNCYHS-GLAAFTGETSPDQLKEIGVKVVMVGHSERRQFLGESNFFCNDKIRFLL-KNEFT-VLYCVGETLSERESGKTLEVLSSQIREGL
Borrelia_turicatae                  ---NVLLGAQNMSYE-DSGARTSEISPSMLLEFGVDYVILGHSECRTYLGDTDEVVNKKVLAGL-KHPFKYLILCIGETLEERENNKTLDVVLNQIRKGL
Thermotoga_maritima                 ---NIKLGAQNVFYE-DQGAFTGEISPLMLQEIGVEYVIVGHSERRRIFKEDDEFINRKVKAVL-EKGMT-PILCVGETLEEREKGLTFCVVEKQVREGF
Fusobacterium_nucleatum             ---NVKIAAENVYPK-IEGAYTGEVSPKMLKDIGVTYVILGHSERREYFKESDEFINQKVKAVL-EIGMK-PILCIGEKLEDREGGKTLEVLAKQIKEGL
Selenomonas_sputigena               ---NIHIGAQNVHWE-KSGAYTGEISTDMLKEIGVDYCVLGHSERREYFGETDEGVNKRAKAAF-AAGIT-PIICCGEPLEIREANTYIDFVTKQIKAAL
Ruminococcus_sp                     ---NIQVGAENMYFE-EKGAYTGEISPAMLVDAGVKYVVLGHSERREYFGETNEDVNKKVLKAF-EHGIT-PIMCCGETLTQREQGVTMDFIRQQVKVGF
Clostridium_botulinum               ---NIKVGAQNMHFE-ESGAYTGEIAPKMLEELGVHYVIIGHSERRQYFNETDETVNKKVKKAF-EHNLI-PIVCCGESLEEREGNITEKVLEGQIKVGL
Clostridium_perfringens             ---NIKVGAQNMHFE-EKGAFTGEIAPRMLEAMNIDYVIIGHSERREYFNETDETCNKKVKAAF-AHNLT-PILCCGETLEQRENGTTNDVIKAQITADL
Thermoanaerobacter_wiegelii         ---NILLGAQDMFWE-EKGAYTGEISPLMLKEIGVKYVVIGHSERRQYFGETDEMVNKKVLSAL-SHGLS-PIVCVGESLSQREEGKTFEVVLNQTKEAL
Brachyspira_murdochii               ---NIKLGAQNLYFE-EKGAFTGQISADMLLAVGCEYVIVGHSECRDIFGEKDELINKKVKRAL-DKNLI-PVLCVGEHLEEREKGITSDIVSSQTKEAF
Acholeplasma_laidlawii              ---HIRIGAQNMHYA-DEGAFTGENSPAQVKTAGAEYILIGHSERRSLFNETDKDVNLKMHAAF-KYDLA-PILCIGEQLDTREANKTKEVLDVQLDKAF
Lactobacillus_reuteri               ---GLKIIAQNAAAE-YSGAFTGEISLRGLADAGVSYVMLGHIERRHLFHEDNELVNRKVLAAL-QMGVT-PIICTDETMVQKEVNGEIHYVFQQLMSVL
Bacillus_subtilis                   --TDLKVGAQNMHFE-ESGAFTGEISPVALKDLGVDYCVIGHSERREMFAETDETVNKKAHAAF-KHGIV-PIICVGETLEEREAGKTNDLVADQVKKGL
```

```
Bacillus_thuringiensis               --TDLQVGAQNMHFE-KNGAFTGEISPVALSDLKVGYVVLGHSERREMFAETDESVNKKTIAAF-EHGLT-PIVCCGETLEERESGKTFDLVAGQVTKAL
Listeria_monocytogenes               --TNLRVSAQNCYFE-DEGAFTGEISPFALADLGVSYVIIGHSERREYFHETDEDINKKAHAIF-KHGMT-PIICCGETLDQREAGQTDTWVRGQIRAAL
Geobacillus_stearothermophilus       --TDLKIGAQTMHFA-DQGAYTGEVSPVMLKDLGVTYVILGHSERRQMFAETDETVNKKVLAAF-TRGLI-PIICCGESLEEREAGQTNAVVASQVEKAL
Staphylococcus_aureus                --QGLEIGAQNTYFE-DNGAFTGETSPVALADLGVKYVVIGHSERRELFHETDEEINKKAHAIF-KHGMT-PIICVGETDEERESGKANDVVGEQVKKAV
Enterococcus_faecalis                --SEVKLAAQNCYWE-NAGAFTGENSPAAIADLGVDYVIIGHSERREYFHETDEDINKKAKAIF-ANGMT-PIFCCGETLETYEAGKTAEWIEGQITKGL
Lactococcus_lactis                   --SELKLAAENSYFE-NAGAFTGENSPAAIVDLGIEYIIIGHSERREYFHETDEDINKKAKAIF-AAGAT-PILCCGETLETFEAGKTAEWVSGQIEAGL
Streptococcus_pneumoniae             --SNLKVAAQNCYFE-NAGAFTGETSPQVLKEIGTDYVVIGHSERRDYFHETDEDINKKAKAIF-ANGML-PIICCGESLETYEAGKAAEFVGAQVSAAL
Erysipelotrichaceae_bacterium        ----LVIAAENCHWE-DSGAFTGEISVPMLQEVGVTHCIIGHSERREMFNDTDETVNKKAKRLI-DAGIT-PILCIGETEAQYDAGDSEKVIRDQLTGSL
Lentisphaera_araneosa                --SPIGIGAQNVHFA-ESGAYTGELSAAMLKEAGIELVIIGHSERRQYFGETNETVNLRTKAAL-AAGIK-PFVCIGETLEEREGGNMESVLKAQIVEGF
Chlamydophila_pneumoniae             --AFLWLGAQNVHPE-LSGAFTGEISLPMLKEVGVEFVLVGHSERRHIFGESDAFIASKVKSVA-QAGLV-PVLCVGESLEVREEGKAHQVIKKQLLLGL
Desulfovibrio_vulgaris               --GFA-IGGQDVYPA-TEGAYTGEIAPGMLLDAGCGWVLTGHSERRHILGEDDETVARKTAFSL-KAGLR-VVLCIGEKLDEREAGRLEDVLAHQLQVGL
Mesorhizobium_opportunistum          --VKV--GAQNMHWA-DAGAWTGEISPVMLTDCGLDLVELGHSERREHFGETDRTVGLKTAAAV-KHGLI-PLICVGETLAERESGEADAVLAKQVEGAL
Bartonella_henselae                  ----ILLGGQNCHFD-DYGPYTGDISAFMLKEAGASHVIIGHSERRTVYQESDAIVRAKVQAAW-RAGLV-ALICVGETLEERKSNKVLDVLTRQLEGSL
Brucella_abortus                     ----VGLGGQDAHFK-TSGAHTGDISPEMLKEAGATHVILGHSERRTDHHESNKLICAKTEAAW-AAGLV-AIVCVGETASERKAERALDVIGDQLSGSL
Rhizobium_etli                       ----LSIGAQDCHQK-ASGAHTGDISAEMIADSFGTYVIVGHSERRTDHAETDHLVRAKAEAAF-AAGLT-AIICIGETADERRAGQALDIIKRQLSASV
Agrobacterium_tumefaciens            ----LLIGAQDCHQN-DTGAHTGDISAEMIADCFGTHVIVGHSERRTDHAETDHLVRAKASAAH-QVDLI-AIVCIGETADERKAGQTLDILKRQLAGSL
Rhodobacter_sphaeroides              ----LMVGGQDCHPK-TSGAHTGDVSAAMLADAGASHVILGHSERRADHGETDSLVRLKAEAAW-GAGLV-AIVCVGETEAQRDAGQTLDVIGAQLAGSV
Ruegeria_sp                          ----IAIGGQDCHAK-TSGAHTGDLSADMLRDAGATYVIIGHSERRADHGEGDADVRAKTEAAQ-AAGLV-AVVCIGETLEEREAGTTLEVVGAQLAGSL
Bradyrhizobium_japonicum             ----VAVGAQDCHPK-ASGAHTGDIAAEMLANAGATAIIVGHSERRADHGEGDALVRQKAEAAW-RAGVT-AIVCIGETQAQRDAGQTLDILRGQLDGSL
Rhodopseudomonas_palustris           GALAVAIGAQDCHAN-ATGAHTGDISAEMLADAGARAIIVGHSERRADHGETDAIVRTKAQAAW-RAGLV-AIVCVGETQGERNAGHTLDVVAGQLAGSL
Methylobacterium_radiotolerans       ----IAIGGQNLHAR-PSGAFTGSISAEMLADLGAKYVIVGHSERRAYHHETDDGVHAKALGAR-RAGLC-GIICVGETIEEREQGRALDIVRAQLAIGL
Rhodospirillum_rubrum                ----IAVGGQDCHAK-AKGAHTGDVSAPMLADLGCQFVIVGHSERRADHGETDALVKAKAEAAL-AAGLI-AVICVGETEAQRDAGETLAVVSGQLAGSL
Zymomonas_mobilis                    ----MTFGAQDCHYD-ESGSHTGCVSAPMLREVGASYVIVGHSERRIEQNETNEEIRAKTTTAI-ANGLV-TIVCIGESRAAHASGQALPIIGAQLDGCI
Gluconacetobacter_hansenii           ----IALGAQDCHQD-PCGAHTGDIAASMLRDLGVQYVILGHSERRRDHHELDETVREKAVAAA-AAGLI-PIICVGESADQRSNGDSQETVGWQIQGSL
Thermus_thermophilus                 --TQVGYGAQDVSAH-KEGAYTGEVSARMLSDLGCRYAIVGHSERRRYHGETDALVAEKAKRLL-EEGIT-PILCVGEPLEVREKGEAVPYTLRQLRGSL
Thermus_aquaticus                    --TPIAYGAQDVSPH-REGAYTGEVSARMLADLGCRYAIVGHSERRRYHGESDALVAEKAKRLL-EEGIT-PILCVGEPLEVRERGEAVPYTLAQLLGSL
Oceanithermus_profundus              --SGVAWGAQDVSAH-TFGAYTGEVAAEQLADLGCRYVVVGHSERRAYWNESDALVAAKARRAM-EAGLV-PILCVGERLEEREAGRAVSFTLEQLAGSL
Deinococcus_deserti                  --AGVAYGGQDVSQH-ESGAYTGEISAAMLRDVGAKYAIVGHSERREYHGETDAVVAAKARQAQ-AHGLT-PIVCVGEGLEVRERGEHVQYTLAQLRGSL
Bifidobacterium_bifidum              --LKIRYGAQAVSVT-TQGAFTGDVSADMIAHLGCSYVIVGHSERRKYHPEDDVNIVDQVRAVL-AAGMQ-PILCVGESFEERRQGIELDFAVGQVHDVT
Bifidobacterium_longum               --LHVAYGAQSVSVT-TQGAFTGDVSADMIAHLGCSYVIVGHSERRKYHPEDDANIVDQVRAVL-AAGMQ-PILCVGESFEERRQGIELDFAVGQVRDVT
Gardnerella_vaginalis                --LPILYGAQAVSVT-AQGAFTGDVSADMLASLGCSMVIVGHSERRKYHPEDDANIVDQVRAVL-AAGMQ-PILCVGESFEERKQGIELDFAVGQVHDVT
Nocardia_farcinica                   --LLLTYGAQDVSVH-ESGAYTGEISASMLAKLGCTFVVVGHSERRQYHHEDDATVLGKAKKAL-EHGLT-PIVCIGEGLNVREAGTHVEYNLEQLRGSL
Mycobacterium_tuberculosis           --LRLTYGAQDLSPH-DSGAYTGDVSGAFLAKLGCSYVVVGHSERRTYHNEDDALVAAKAATAL-KHGLT-PIVCIGEHLDVREAGNHVAHNIEQLRGSL
Corynebacterium_glutamicum           --LEVTFGAQDVSQH-ESGAYTGEVSASMLAKLNCSWVVVGHSERREYHNESDELVAAKAKAAL-SNGIS-PIVCVGEPLEIREAGTHVEYVVEQTRKSL
Propionibacterium_acnes              --LPIAYGAQDISAH-DDGAYTGEVSGAMLSKLGCRYVVVGHSERREYHNESDELVNAKAKKVI-ENGMT-PIICCGEALEVRKAGKHVEHTVGQIKADL
Actinomyces_odontolyticus            --LGIKYGAQDVSIH-DNGAYTGEISTDMLTKLGVSYVVMGHSERREYHGESDELVGAKARKVF-DAGMT-PILCCGEALEVRKAGTYVEFVLGQIRAAL
Microbacterium_testaceum             --IEFALGGQDLSAH-DSGAYTGEISGQFLAKLNARYVIIGHSERRQFHNETDEVVAAKTQAAL-RHGLV-PVICVGETSEDLEKFGASAVPVGQLEVAL
Moritella_marina                     -GSAIILGAQNTDLN-NSGAFTGDMSPAMLKEFGATHIIIGHSERREYHAESDEFVAKKFAFLK-ENGLT-PVLCIGESDAQNEAGETMAVCARQLDAVI
Photobacterium_profundum             -GSAIILGAQNSDLN-NSGAFTGDMSPAMLKEFGASHIIIGHSERREYHNESDEFVAKKFAFLK-ENGLT-PVLCIGESDAQNEAGETMAVCARQLDAVI
Vibrio_cholerae                      -GNKLILGAQNTDTH-NSGAYTGDMSPAMLKDFGASHIIIGHSERRDYHKESDEFVAKKFAFLK-ENGLT-PVFCIGETEAQNEAGETEAVCARQINAVI
Shigella_flexneri                    --SHIMLGAQNVDLN-LSGAFTGETSAAMLKDIGAQYIIIGHSERRTYHKESDELIAKKFAVLK-EQGLT-PVLCIGETEAENEAGKTEEVCARQIDAVL
Escherichia_coli                     --SHIMLGAQNVDLN-LSGAFTGETSAAMLKDIGAQYIIIGHSERRTYHKESDELIAKKFAVLK-EQGLT-PVLCIGETEAENEAGKTEEVCARQIDAVL
Salmonella_enterica                  --SHIMLGAQNVDLN-LSGAFTGETSAEMLKDIGAQYIIIGHSERRTYHKESDELIAKKFAVLK-EQGLT-PVLCIGETEAENEAGKTEEVCARQIDTVL
Klebsiella_pneumoniae                --SHIHLGAQNVDVN-LSGAFTGETSAEMLKDIGAQYIIIGHSERRTYHKESDELIAKKFAVLK-EQGLT-PVLCIGETEAENEAGKTEEVCARQIDAVL
Enterobacter_aerogenes               --SHIHLGAQNVDVN-LSGAFTGETSAEMLKDIGAQYIIIGHSERRTYHKESDELIAKKFAVLK-EQGLT-PVLCIGETEAENEAGKTEEVCARQIDAVL
Serratia_symbiotica                  --SRIALGAQNVDIN-LSGAFTGEISANMLKDVGAQYIIIGHSERRTYHKETDGAISEKFAVLK-EAGLI-PVLCIGETEAENEAGKTEEVCARQIDAVL
```

```
Yersinia_pestis                    --SRIALGAQNVDVN-LSGAFTGETSAEMLKDIGAQYIIIGHSERRTYHQESDELIAKKFGVLK-EIGLI-PVLCIGESEAENEAGQTEAVCAKQLDAVL
Actinobacillus_pleuropneumonia     --SVIALGAQNVDVN-VQGAFTGDISTEMLKDFGAKYIIIGHSERRTYHKECDTFIAKKFAALK-AAGLV-PVLCIGETEAENETGQTEAVCAKQIDAVI
Pseudomonas_aeruginosa             --KAIDVGAQNSAVEPMQGALTGETAPSQLADVGCSMVLVGHSERRLILGESDEVVSRKFAAAQ-SCGLV-PVLCVGETRAEREAGKTLEVVARQLGSVI
Marinomonas_posidonica             --SGLVLAAQNLSQY-EKGAYTGEVSTAMLTDFKVTYVLVGHSERRSLYGETDEVVAEKVKMAL-SAGLK-PILCIGETLEEREAEQTLLVCERQVAAVI
Xylella_fastidiosa                 --EGLAFGAQNVSHH-DKGAYTGEVSAAMVADVGAHYTLVGHSERREYHHEDSELVARKFAAAL-SAGLR-PILCVGESLPQREAGQAEVAIAMQLAPVL
Xanthomonas_campestris             --HHLAFGAQDVSSN-EKGAYTGEVSASMLVDVGAEYGLVGHSERRQYHQESSELVARKFAAAM-HAGLI-PVLCVGESLEQREAGQTEAILRAQLEPVL
Marinobacter_algicola              --KKLAVGVQNVACW-SSGAYTGEISAAMAKDCGCGFTLVGHSERRQLFGETDEQVATKVEQAL-ASGLV-AVICVGETLEERDAGKAEDVVSRQVRKAV
Aeromonas_caviae                   ---RVKLGAQNVSQH-RQGAYTGEISCEMLVEAGCRYVLVGHSERRRLFGETSFIVAEKFAAVR-AAGLI-PILCLGESGAARQARRTFEVIAEELDIVI
Pseudoalteromonas_haloplanktis     ----VSTGTQTVSEN-TPGAFTGEVDAQLIKELGAQYVLVGHSERRSIYKESNDVVAAKFARAQ-QVGLT-PILCVGESESEQENGKTEQIVAAQIDAVI
Legionella_pneumophila             ---RIVVGAQNVYPK-DYGAYTGELSAPMLKDFDCRYVLVGHSERRQFFHEDENFVAQKFHHVK-DHGMI-PILCVGETLSERENGKTEQIIAQQVLAVS
Ralstonia_solanacearum             ---AVAWGAQDVSAE-TRGAFTGEVAASMLSEFGCGYVIVGHSERRTYHAETDAQVATKALRAL-EHGIT-PIVCVGETLVQREAGETEQVVARQLDAVL
Burkholderia_sp                    ---SVVWGVQDVSAF-THGAYTGEVAAPMVTDFGATFAIVGHSERRAYHRESAELVAVKAQRVL-DAGLT-PIVCVGETLEEREAGSTEQVVGGQLDEVL
Bordetella_pertussis               ---AIGWGAQDVSAH-AKGAYTGEVAAPMLAEFGCRWVLVGHSERRTLHAESDQLVADKARAAL-EAGLT-PVVCVGESLQEREGGNTLGVIERQLEPVL
Nitrosococcus_watsonii             ---VINWGAQNLSHH-EMGAYTGEIAPSMLADLGCRFVIIGHSERRTLYGETDSLVAEKAIAAQ-KVDII-PIICVGETLQEREQNITEQVVERQLNAVL
Coxiella_burnetii                  ---QISWGAQDVSEF-ERGAYTGEVSAAMLRDFHCRYVIVGHSERRQRHGETNEQVAAKVRAAL-RCGIR-PIICVGETEKQRNANQTLSVIKEQLAVVL
Variovorax_paradoxus               ---SLALGAQDVSAH-PQGAFTGEQSAAMLKDFGVRYAIVGHSERRQYHGETDETVAAKAAAAL-ANGIT-PIVCVGETLAEREAGQTEEVVKRQLAAVI
Halomonas_elongata                 ---RAALGAQTLSDQ-PSGAFTGEVSGEMLREVGTSLVLVGHSERRTQFGEDDSAVLARVRRAL-ECELT-PVLCLGETLEERDADRGEAVVIGQLEAVL
Tenebrio_Molitor                   --ASIGVAAQNCYKV-PKGAFTGEISPAMIKDVGADWVILGHSERRQIFGESDELIAEKVCHAL-ESGLK-VIACIGETLEEREAGKTEEVVFRQTKAIA
Bombyx_mori                        --DNVEVAAQNCWKS-PKGAFTGEISPAMIKDVGVNWVILGHSERRTIFGEKDELVAEKVAHAL-ESGLK-VIACIGETLEERESGKTEEVVFRQLKALV
Apis_mellifera                     --NNISIAGQNTYKV-AKGAFTGEISPAMLLDNGIPWVILGHSERRNIFGENDELIAEKVAHAL-ESGLK-VIACIGEKLEEREAGKTDEVVFRQTKAIA
Aedes_aegypti                      --DSVGVAAQNCYKV-AKGAFTGEISPAMLKDLNIGWVILGHSERRAIFGESDELVADKVAHAL-AEGLK-VIACIGETLQEREAGQTEAVCFRQTKAIA
Anopheles_gambiae                  --ETIGVAAQNCYKV-AKGAFTGEISPAMLKDLGLGWVILGHSERRAIFGETDELIAEKVAHAL-AEGLK-VIACIGETLQEREAGQTEAVCFRQTKAIA
Drosophila_melanogaster            --CELGLAGQNAYKV-AKGAFTGEISPAMLKDIGADWVILGHSERRAIFGESDALIAEKAEHAL-AEGLK-VIACIGETLEEREAGKTNEVVARQMCAYA
Rhipicephalus_microplus            --PSVALAAQNCYKV-EQGAFTGEISPGMIKDCGGQWVILGHSERRHVFKEDDVLIGEKIKHAL-ESGLN-VIACIGELLEDREAGRTEEVCFRQIKHIA
Ixodes_scapularis                  --AAIAVSAQNCYKV-EKGAFTGEISPAMIKDCGATWVILGHSERRNVFKESDELIGDKVHHAL-ESGLN-VIACIGELLEEREAGKTEEVVYRQTAAIA
Anolis_carolinensis                --AKFGVAAQNCYKV-AKGAFTGEISPAMIKDIGATWVILGHSERRHIFGESDELIGQKVAHAL-AEGLG-VIACIGEKLDEREAGITEKVVFEQTKAIA
Gallus_gallus                      --AKIGVAAQNCYKV-PKGAFTGEISPAMIKDIGAAWVILGHSERRHVFGESDELIGQKVAHAL-AEGLG-VIACIGEKLDEREAGITEKVVFEQTKAIA
Orcinus_orca                       --HKIAVAAQNCYKV-ANGAFTGEISPGMIKDCGATWVVLGHSERRHVFGESDELIGQKVAHAL-AEGLG-VIACIGEKLDEREAGITEKVVFEQTKVIA
Bos_taurus                         --PKIAVAAQNCYKV-ANGAFTGEISPGMIKDLGATWVVLGHSERRHVFGESDELIGQKVAHAL-AEGLG-VIACIGEKLDEREAGITEKVVFEQTKVIA
Canis_lupusfamiliaris              --AKIAVAAQNCYKV-TNGAFTGEISPGMIKDCGATWVVLGHSERRHVFGESDELIGQKVAHAL-AEGLG-VIACIGEKLDEREAGITEKVVFEQTKVIA
Odobenus_rosmarus                  --AKIAVAAQNCYKV-TNGAFTGEISPGMIKDCGATWVVLGHSERRHVFGESDELIGQKVAHAL-AEGLG-VIACIGEKLDEREAGITEKVVFEQTKVIA
Oryctolagus_cuniculus              --PKIAVAAQNCYKV-TNGAFTGEISPGMIKDCGATWVVLGHSERRHVFGESDELIGQKVAHAL-SEGLG-VIACIGEKLDEREAGITEKVVFEQTKVIA
Nomascus_leucogenys                --PKIAVAAQNCYKV-TNGAFTGEISPGMIKDCGATWVVLGHSERRHVFGESDELIGQKVAHAL-AEGLG-VIACIGEKLDEREAGITEKVVFEQTKVIA
Pan_troglodytes                    --PKIAVAAQNCYKV-TNGAFTGEISPGMIKDCGATWVVLGHSERRHVFGESDELIGQKVAHAL-AEGLG-VIACIGEKLDEREAGITEKVVFEQTKVIA
Homo_sapiens                       --PKIAVAAQNCYKV-TNGAFTGEISPGMIKDCGATWVVLGHSERRHVFGESDELIGQKVAHAL-AEGLG-VIACIGEKLDEREAGITEKVVFEQTKVIA
Pongo_abelii                       --PKIAVAAQNCYKV-TNGAFTGEISPGMIKDYGATWVVLGHSERRHVFGESDELIGQKVAHAL-AEGLG-VIACIGEKLDEREAGITEKVVFEQTKVIA
Macaca_mulatta                     --PKIAVAAQNCYKV-TNGAFTGEISPGMIKDCGATWVVLGHSERRHVFGESDELIGQKVAHAL-AEGLG-VIACIGEKLDEREAGITEKVVFEQTKVIA
Ochotona_princeps                  --AKIAVAAQNCYKV-ANGAFTGEISPGMIKDCGATWVILGHSERRHVFGESDELIGQKVAHAL-AEGLG-VIACIGEKLDEREAGITEKVVFEQTKVIA
Sus_scrofa                         --PKIAVAAQNCYKV-ANGAFTGEIGPGMIKDLGATWVVLGHSERRHVFGESDELIGQKVAHAL-AEGLG-VIACIGEKLDEREAGITEKVVFEQTKVIA
Mus_musculus                       --PKIAVAAQNCYKV-TNGPFTGEISPGMIKDLGATWVVLGHSERRHVFGESDELIGQKVSHAL-AEGLG-VIACIGEKLDEREAGITEKVVFEQTKVIA
Rattus_norvegicus                  --PKIAVAAQNCYKV-TNGAFTGEISPGMIKDLGATWVVLGHSERRHIFGESDELIGQKVNHAL-SEGLG-VIACIGEKLDEREAGITEKVVFEQTKAIA
Xenopus_laevis                     --AKIALSAQNCYKV-AKGAFTGEISPAMIKDCGATWVILGHSERRHVFGECDELIGQKVAHAL-SEGIG-VIACIGEKLDQREAGITEKVVFEQTKAIA
Xenopus_tropicalis                 --AKFAVSAQNCYKV-AKGAFTGEISPAMIKDCGATWVILGHSERRHVFGESDELIGQKVAHAL-SENVG-VIGCIGEKLDQREAGITEKVVFEQTKAIA
Danio_rerio                        --PNIDVAAQNCYKV-AKGAFTGEISPAMIKDCGVKWVILGHSERRHVFGESDELIGQKVAHAL-ENGLG-VIACIGEKLDEREAGITEKVVFAQTKFIA
Ictalurus_punctatus                --AKIGVAAQNCYKV-AKGAFTGEISPAMIKDCGVEWVILGHSERRHVFGESDELIGQKVAHAL-ENGLG-VIACIGEKLDEREAGITEKVVFAQTKAIA
Oreochromis_niloticus              --PKFGVAAQNCYKV-PKGAFTGEISPAMIKDCGVNWVILGHSERRHVFGESDELIGQKTAHAL-ENGLG-VIACIGEKLDEREGGITEKVVFAQTKVIA
```

```
Nematostella_vectensis            --QNIGVAAQNCYKV-ASGAFTGEISPAMIKDIGCEWVIIGHSERRNIFGEKDELIGEKVAHAL-SANVK-VIACIGELLSEREAGKTQEVVFRQIKAIA
Schistosoma_mansoni               --KEIHVAAQNCYKV-SKGAFTGEISPAMIKDIGCDWVILGHSERRNIFGESDELIAEKVQHAL-AEGLS-VIACIGETLSERESNKTEEVCVRQLKAIA
Strongylocentrotus_purpuratus     --AGVFVAAQNCYKV-PKGAFTAETSPAMLQDLGVGWVILGHSERRHVFGESDELIAQKVAHAL-SCKLK-VVACIGEKLDEREKGQTNEVVYRQMRALA
Caenorhabditis_elegans            --AGVLVAAQNCYKV-PKGAFTGEISPAMIKDLGLEWVILGHSERRHVFGESDALIAEKTVHAL-EAGIK-VVFCIGEKLEEREAGHTKDVNFRQLQAIV
Caenorhabditis_remanei            --AGVLVAAQNCYKV-PKGAFTGEISPAMIKDLGLEWVILGHSERRHVFGETDALIAEKTVHAL-ESGIK-VIFCIGEKLEEREAGQTKDVNFRQLQAIV
Brugia_malayi                     --NGIEVSAQNCYKV-EKGAFTGEISPAMIKDLGLHWVILGHSERRHIFGESDELIAEKVLHAV-DSGLQ-TIFCCGEKLDEREAGKTKAVNFRQLQAVI
Trichinella_spiralis              --PAVGLSAQNCYKV-EKGAFTGEVSASMLRDIGCDWVILGHSERRHILHESDELVAEKVSMAL-NSGLG-VIFCVGEKLDEREGGKTKDVCFRQLASLL
Ricinus_communis                  --PDFHVAAQNCWVK-KGGAFTGEVSAEMLVNLSIPWVILGHSERRLLLSESNEFVGDKVAYAL-SQGLK-VIACVGETLEQREAGSTMDVVAAQTKAIA
Vitis_vinifera                    --PDFHVAAQNCWVK-KGGAFTGEISAEMLVNLGIPWVIIGHSERRLLLNESNEFVGEKVAYAL-SKGLK-VIACVGETLEQRESGSTMEVVAAQTKAIA
Arabidopsis_thaliana              --SDFFVAAQNCWVK-KGGAFTGEVSAEMLVNLDIPWVILGHSERRAILNESSEFVGDKVAYAL-AQGLK-VIACVGETLEEREAGSTMDVVAAQTKAIA
Zea_mays                          --QEFHVAAQNCWVK-KGGAFTGEVSAEMLVNLGVPWVILGHSERRALLGESNEFVGDKVAYAL-SQGLK-VIACVGETLEQREAGSTMDVVAAQTKAIA
Oryza_sativa                      --PEIQVAAQNCWVK-KGGAFTGEVSAEMLVNLSIPWVILGHSERRSLLGESNEFVGDKVAYAL-SQGLK-VIACVGETLEQRESGSTMDVVAAQTKAIS
Fragaria_vesca                    --PDFQVAAQNCWVK-KGGAFTGEVSAEMLKNLEVPWVILGHSERRALLGETNEFVGDKVAYAL-SQGLK-VIACVGETLEQREAGSTVEVVAAQTKAIA
Glycine_max                       --PDFHVSAQNCWVR-KGGAYTGEVSAEMLVNLGIPWVIIGHSERRQLLNELNEFVGDKVAYAL-QQGLK-VIACIGETLEQREAGTTTAVVAEQTKAIA
Cucumis_sativus                   --SDIQVAAQNCWVR-KGGAFTGEVSAEMLVNLGIPWVILGHSERRLILKESNEFVGDKVAYAL-SQGIK-VIACVGETLEQRESGSTIEVVAAQTKAIA
Perkinsus_marinus                 --SNIQISAQNCSKT-GPGAYTGEITAEMIKDAGLNWTILGHSERRHLFNETNEDLAAKVAKAE-AAGLK-IIFCIGELLEEREAGKTEEVCATQMDAIV
Aspergillus_niger                 --EKIGVAAQNVFDK-PNGAFTGEISVEQLRDVKIDWTIIGHSERRVILKESDEFIARKVKAAI-DGGLS-VIFCIGETLEEREANKTIDVVTKQLNAVA
Penicillium_chrysogenum           --PKIGVAAQNVYDK-PNGAFTGEISVEQLRDNKINWAVIGHSERRVILKESDEFIARKTKAAI-EGGVS-VIFCIGETLEEREANKTIEVVTRQLNAVA
Paracoccidioides_brasiliensis     --GQVAVSAQNVFDK-PNGAFTGELSVEQLRDEKITWTLAGHSERRVLLREDDEFVARKTKAAI-NGGLN-VILCIGESLEEREAGKTIDVVTRQLDAVA
Schizosaccharomyces_pombe         --KDIGVGAQNVFDK-KNGAYTGENSAQSLIDAGITYTLTGHSERRTIFKESDEFVADKTKFAL-EQGLT-VVACIGETLAEREANETINVVVRQLNAIA
Saccharomyces_cerevisiae          --PQVTVGAQNAYLK-ASGAFTGENSVDQIKDVGAKWVILGHSERRSYFHEDDKFIADKTKFAL-GQGVG-VILCIGETLEEKKAGKTLDVVERQLNAVL
Kluyveromyces_lactis              --KEVKISAQNAYSK-ASGAYTGENSVEQIKDVGAEWVILGHSERRTYFNETDEIVAEKTKFAL-DSGVK-VILCIGETLEEKQQNITLQVVQRQLQAVL
Candida_dubliniensis              --PTVAVGAQNVFDK-ACGAFTGETCASQILDVGATWTLTGHSERRTIIKESDEFIAEKTKFAL-DNGVK-VILCIGETLEERKGGITLDVCARQLDAVS
Clavispora_lusitaniae             --STVAVAAQNVFNK-ASGAYTGEISPEQLKDLGATWTLTGHSERRTIIKESDEFIAEKTKFAL-DSGVS-VILCIGETLEERKAGITIDVCARQLDAVS
Chlamydomonas_reinhardtii         --DKYQLSAQNAWIG-GNGAFTGEVSAEQLTDFGVPWVILGHSERRSLFGESNEVVAKKTSHAL-AAGLG-VIACIGETLEQRNSGSVFKVLDAQMDALV
Volvox_carteri                    --DKYQLAAQNCWIG-GNGAYTGEVSAEQLQDFGLPWVILGHSERRSLCGETNQVVAKKTAHAL-DVGLG-VIACVGETLDQRNSGAMFQVLDGQMQALV
Dictyostelium_fasciculatum        --QRVQVAAQNCYSE-TKGAYTGEISAEMIVDIGLKWVILGHSERRNLFKESNQLIAKKTQHAL-SQGLS-VIFCIGELLEQRETNKTEQVLDEQLGFLL
Entamoeba_histolytica             -GANILVSAENAWTK-S-GAYTGEVHVGMLVDCQVPYVILGHSERRQIFHESNEQVAEKVKVAI-DAGLK-VIACIGETEAQRIANQTEEVVAAQLKAIN
Cryptosporidium_parvum            --GVFKIGSQNISCT-GNGAFTGEVSCEMLKDMDVDCSLVGHSERRQYYSETDQIVNNKVKKGL-ENGLK-IVLCIGESLSERETGKTNDVIQKQLTEAL
Cryptosporidium_muris             ----INIGSQDISST-GNGAYTGEVSCDILKDYNIHCTLIGHSERRQYYSETDSVVNAKVLHAL-KHNFT-VVLCIGESLQERESGKTNDVIKHQLEFAL
Plasmodium_falciparum             -----STGIQNVSKF-GNGSYTGEVSAEIAKDLNIEYVIIGHFERRKYFHETDEDVREKLQASL-KNNLK-AVVCFGESLEQREQNKTIEVITKQVKAFV
Plasmodium_vivax                  -----HTGIQNVSKF-GSGSYTGEISAEIAKDLNIEYVIIGHFERRKYFNETDEDVKDKLQQCL-KNNLK-VVVCFGESLEQREKNQTIDVITKQVNSFV
Paramecium_tetraurelia            -----QVALQNSSLT-KVGAYTGEISVEQVKDLAIPWVILGHSERRQYYGETNEIVGKKTRIAL-DYQLS-VMACVGEKLAEREAGQTTQVIQAQLDAIK
Tetrahymena_thermophila           -----QVSVQNTSLT-GTGAYTGEISVAHVKDLGLNWAILGHSERRQYYGETNEVVANKVKYAV-ENGLD-VIACVGEHLADREANQTNQVVEGQLEAIR
Toxoplasma_gondii                 -----HVGLQNCSKT-HNGAFTGEISVEMIKDFGLRWILAGHSERRQYYGESDEVVAEKVNIILQEKDLN-VVLCVGEQLKDREANKTNDVVDAQLAACL
Trypanosoma_brucei                ----FVIAAQNAIAK-S-GAFTGEVSLPILKDFGVNWIVLGHSERRAYYGETNEIVADKVAAAV-AAGFM-VIACIGETLQERESGRTAVVVLTQIAAIA
Trypanosoma_cruzi                 ----FQIAAQNAITR-S-GAFTGEVSLQILKDYGIKWVVLGHSERRLYYGETNEIVAEKVAQAC-AAGFH-VIVCVGETNEEREAGRTAAVVLTQLAAVA
Leishmania_mexicana               ----YVISAENAIAK-S-GAFTGEVSMPILKDIGVHWVILGHSERRTYYGETDEIVAQKVSEAC-KQGFM-VIACIGETLQQREANQTAKVVLSQTSAIA
Leishmania_donovani               ----YAVSAENAIAK-S-GAFTGEVSMPILKDLGINWVILGHSERRTYYGETDEIVAQKVADAC-KQGFM-VIACIGETLQQREANQTAKVVLSQTSAIA
Giardia_lamblia                   ----LRIAAQNVYLE-GNGAWTGETSVEMLQDMGLKHVIVGHSERRRIMGETDEQSAKKAKRAL-EKGMT-VIFCVGETLDERKANRTMEVNIAQLEALG
Trichomonas_vaginalis             --KDWKVSAENVFTK-PNGAFTGEVTVPMIKSFGIEWTILGHSERRDILKEDDEFLAAKAKFAL-ENGMK-IIYCCGEHLSEREAGKASEFVSAQIEKMI
Sphingobacterium_sp               ---NVSVGAQNIHQE-DAGAYTGEISAKQVRSTGAEYVILGHSERRAYFGESDELLAQKVNIAL-THGLI-PIFCIGETREERESGDFFDVIKTQLEKGV
Pedobacter_saltans                ---NVFVGAQNAHQE-ASGAYTGEISTGMLKSVGVEYVILGHSERRQYFGENNELLAKKTNTAL-ANDLK-PIFCIGETLAERESNQHFDVIKTQLAEAT
Marivirga_tractuosa               ---QIVLAGQNCHQE-DAGAFTGEVSAPMLKSLGAQYVILGHSERREHFSETDEELAKKTDNAL-KNDLK-VIFCCGEPLEIREADTQNEYVTKQLTNSL
Cyclobacterium_marinum            ---NIALGAQNCSDK-EAGAYTGETSAAMLASFGAEYVIIGHSERRSMFNESNELLTEKTKQAL-SNGLT-PIFCCGEPLEIREADTHEDYVKAQLTESL
Leadbetterella_byssophila         ---KVALAAQNCYPK-ASGAYTGEVSVPMLKSVGVKYVIIGHSERREYFAESDAFIAEKVDAIL-AEGLT-PIFCCGETLSQREAGIHFDFVKGQLTNSL
```

```
Haliscomenobacter_hydrossis        ---SLYLGAQNCHQE-DKGAYTGEISVDMLKSVGVSYVILGHSERREYFGESDELLAKKTDKVL-AAGLL-PIFCCGESLDIRDAGTHVAHVQAQIKAGL
Parabacteroides_distasonis         ---KIGVAAENCADK-EKGAYTGEVSAAMVASTGAKYVILGHSERRAYYHETPEILKTKVELAL-ANGLT-PIFCIGEVLEEREAGKHFEVVDAQIAGSL
Bacteroides_fragilis               ---KIGVGAENCADK-ESGAYTGEVSAAMVASTGAKYVILGHSERRAYYGETVEILKDKVKLAL-ANGLT-PIFCIGEVLEEREANKQNEVVAAQLA-SV
Prevotella_ruminicola              ---IIGLGAENCADK-EKGAFTGEVSAEMVKSTGAQYVILGHSERREYYKETPEILKEKVLLAQ-KNDLK-VIFCIGESLEEREAGKQNEVVKAELEGSV
Alistipes_shahii                   ---DVALGAQDCATE-AKGAYTGEIAASMIAALGCKYVILGHSERRQYYGETSATLNKKMAQAY-ANGLI-PIYCVGENLDEREAGKHFDVVKAQIEEVV
Porphyromonas_asaccharolytica      ---DIEYAAQDCSAH-DAGAYTGEVSAEMIRSCGCTYVIIGHSERRAYHKESAELLAQKIDQAL-AHGLK-VIFCVGEQQAEREGNRYFEVVDEQLRGSL
Cellulophaga_lytica                ---TIQVIAQNMHFA-DNGAYTGEISADMLLDIGINTVIIGHSERRAYFGETDEILAKKVIKAL-EKNLR-VVFCFGEELEDRKSDNHFNIVASQLKNAL
Kordia_algicida                    ---NVEVIAQNMHQE-AKGAYTGEVSARMLKSVGIKTVILGHSERRAYFGETDELLAKKVDAAL-ANELE-IIFCFGEELEDRKAGNHFNIVENQLKNAL
Flavobacterium_psychrophilum       ---NIRVAAQNMHQA-ENGAYTGEISADMLKSIGVNTVILGHSERRAIFHETDAIISFKVDTAL-RHEMT-VIFCFGEELKDRQNKQHFNIVENQLRDGL
Neisseria_gonorrhoeae              N--RILTCAQDVSRFPDNGAYTGEVSAEMLADTGTDIVLIGHSERSLYFGEKNEIQRRKMENVL-NVGLI-PLLCVGESLEERETGKEHEVIAHQLS-IL
Francisella_tularensis             ------VGLQNIT-FYDDGAYTGEISARMLEDIGCDYLLIGHSERRSLFAESDEDVFKKLNKII-DTTIT-PVVCIGESLDDRQSGKLKQVLATQLSLIL
Mycoplasma_genitalium              ----LLLFAQDANFI-ESGSYTGTVSFTQLQDIGVNNSIIGHSERRKYYNETSAVINQKLFACL-KASMQ-VVLCIGEAL-----GQEISFLKTDLTNCL
Ureaplasma_urealyticum             ---KVKLYAQNPSAY-SKGPYTGQISCLQLLDSNIKNTLVGHSEIRIDCSQS--IIDQKTKICM--DLLDQVIICIGEPLDVYEQK--KSLSFVLSQLAN
Ureaplasma_parvum                  ---KVNLYAQNPSLH-NKGPYTGQISCLQLLDINIKNALVGHSEIRIDFSQS--IIDQKIKISM--DLLEQVIICVGETFDAYKQN--KSLNFVLNQLAN
Buchnera_aphidicola                ---NIFLGSQNVDIN-LNGAFTGETSILMLRDIGVKYVIIGHSERRFLHHETDDIIAKKFHLIK--KSNLTPILCVGETEIEKKHN--QTEQVIQRQLNL
Campylobacter_jejuni               ---NFIQGAQNFYPC-VNGAFTGELGKEHLDEFGIKCVLIGHSERRALGDEE--FIKAKFDFAK--EHGYKIVFCIGENLDTKNSG--KTLEFLKKQLEI
Helicobacter_pylori                ---HFTLGVQNAYPR-DCGAFTGEITSKHLEELKIHTLLIGHSERRTLLKESPSFLKEKFDFFK--DKNFKIVYCIGEELTTREKGFKAVKEFLSEQLEN
Orientia_tsutsugamushi             ---HFSAQDVSMITE-SSGPYTGEISASMLKNLNVNYAIVGHSERRLLFYEDANTIALKVRNCI--NNAIVPIVCIGEPIEARKNK--TYLQYLAQQLSS
Wolbachia_sp                       ---NINIGAQNCHHK-KFGSYTGEISAEMLKELGCTYVILGHSER---ANEKDSEIKLKSEIAI--ESGLHPIICVGENSEDYKNE--KTKEVIEYQCKN
Methanocaldococcus_Jannaschii      ------VYAQHIDNI-NPGSHTGHILAEAIKDCGCKGTLINHSEKRMLLADIEAVINKCKNLGL------ETIVCTNNINTSKAVAALSPDC--------
Methanococcus_voltae               ------VYSQHMDAI-KPGSNTGKILPEAVKSTGAFGTLINHSEKRLLLSDIEEIINKSKELKL------ESVVCTNNIGVSKAVSALSPDY--------
Methanothermococcus_okinawensi     ------VYAQHFDAV-SPGSHTGHVLAETLKDCGLNGSLLNHSEKRMILADLEKSIEIAKNYNL------ETIVCTNNIGVSKAVAALNPNM--------
Pyrococcus_Woesei                  ------VFAQHIDPI-KPGSHTGHVLPEAVKEAGAVGTLLNHSENRMILADLEAAIRRAEEVGL------MTMVCSNNPAVSAAVAALNPDY--------
Pyrococcus_furiosus                ------VFAQHIDPI-KPGSHTGHVLPEAVKEAGAVGTLLNHSENRMILADLEAAIRRAEEVGL------MTMVCSNNPAVSAAVAALNPDY--------
Thermofilum_pendens                ------VFSQHVDDV-PPGSYTGHVTLEAVKDAGAVGTMVNHSERRVRADQVDVIVKRARSLGL------TTVVCTNTPEVTAAMAALGPDM--------
Methanothermobacter_thermautot     ------VLAQHIDAV-DAGGHTGSILAECARDAGAAGTLINHSEKRMQLADIEWVISRMKELEM------MSVVCTNNVMTTAAAAALGPDF--------
Methanosaeta_thermophila           ------VYAQHVDGV-GFGSFTGHITAASVKAAGASGSLINHSERRLRLADIEASLRACRSEGL------TSIICTNNVATTRAAAALRPDY--------
Natronobacterium_gregoryi          ------TWAQHVDPI-EHGSNTGHTLAESVADAGAVGTLVNHSERRLKLADIDGAVRAAERADL------ETVVCANNPEQIGAGAALGPDA--------
Halobacterium_salinarum            ------TYAQHVSPT-EHGSHTGSVLAESVADNGAVGTLLNHSEHRRRLADIDGSVAAAERAGL------DTVVCANNPAQVAAAAALGPDA--------
Halogeometricum_borinquense        ------TWAQHVDPN-GYGSHTGSTLAEAVAEAGADGTLINHSEKRLKLADIDGSVQAAARAGL------ETCVCANNPAQIGAVAALGPDS--------
Methanosphaerula_palustris         ------VFAQHLDGV-VPGAFTGHVTAAAVRQTGAVGTLINHSERRLTLAEIEASVRAATEAGL------VTVICSNNDMTSAAAAALSPTY--------
Thermoproteus_tenax                ------VYAQGADVE-AGGAHTAHVSLENIKEAGGSGVILNHSEAPLKLNDLARLVAKAKSLGL------DVVVCAPDPRTSLAAAALGPHA--------
Pyrobaculum_islandicum             ------VYAQGADVE-TPGAYTAHIAVDNIKAVGASGLILNHSEAPLALNQLSKLAARAKSIGL------DVVICAPDPVTSLAAAALGPHA--------
Cenarchaeum_symbiosum              ------VLAQHIDDK-GVGSTTGYVVPELLGESGVSGALINHSEHRVSADQVASLVPRLRGLDM------ISVVCVKDSAEAANLSRHRPDY--------
Nitrosopumilus_maritimus           ------IFAQHIDDS-KVGSTTGFVIPELLKKSQVNGSLINHSEHRISSKEITKLVSKLKELKM------TSIVCVKDVAEARKYAKLNPNY--------
Desulfurococcus_kamchatkensis      ----VKVFAQHADPV-EPGAITGFIPLEGLKNLGAHGVILNHSEHRLKISDINYLVKKARRLGI------ETLVCADTPETGAAVAVLGPDM--------
Pyrolobus_fumarii                  ----VIVYAQHVDPV-EPGAHTGSVPVEAVKEAGARGSLVNHSERKLRLDEIERVVERLKVVGL------EQLVCADTPRAAAAIAVFEPTY--------
Acidilobus_saccharovorans          ------VYIEHADPV-DYGAYTGYLPAKAARLLGVRGILVNHSEHKMIYRDVAKVVETASKDGI------ETMACADTPGEAAGLAYLRPTY--------
Sulfolobus_islandicus              ------IYAEHVDPV-PLGAYTGAVLPEMIKDADAKGTLINHSERRLRADEIDDVLKRTRKLGL------SSILCVDRYELVYPFSLLRPDA--------
Clustal Consensus                                     .  *.                .* *      :                       *
```

```
                                      210       220       230       240       250       260       270       280       290       300
                                  ....|....|....|....|....|....|....|....|....|....|....|....|....|....|....|....|....|....|....|....|
Roseiflexus_castenholzii          TGMTPEQ--IGRIVIAYEPVWAIGTGDTATPADAQAMHE-TIRRILGDMAG--SDTAATINILYGGSVKPDNIDDLMAQPDIDGALVGGASLKADSFLR-
Chloroflexus_aurantiacus          LEVPPDQ--MANVVIAYEPIWAIGTGDTATPADAQAMHA-AIRATLAELYG--SEIAATVRIQYGGSVKPDNIDELMAQPDIDGALVGGASLQAASFLR-
Nostoc_punctiforme                VDIDQN-----NLVIAYEPIWAIGTGETCEAVEANRIIG-LIRSQLSNPN---------VSIQYGGSVKPNNIDEIMAQPEIDGVLVGGASLEPESFAR-
Anabaena_variabilis               INVDQT-----NLVIAYEPIWAIGTGDTCETTEANRVIG-LIRSQLKNPD---------VPIQYGGSVKPNNIDEIMAQPEIDGVLVGGASLEAASFAR-
Synechocystis_sp                  VNVDQS-----NLVIAYEPIWAIGTGDTCAATEANRVIG-LIREQLTNSQ---------VTIQYGGSVNANNVDEIMAQPEIDGALVGGASLEPQSFAR-
Cyanobacterium_aponinum           VNVDQN-----NLVIAYEPIWAIGTGDTCEAAEANRVIG-LIRSQLTNKN---------VTIQYGGSVNPKNVDEIMAQPEIDGALVGGASLDPESFAR-
Crocosphaera_watsonii             VDVDQS-----NLVIAYEPIWAIGTGDTCESTEANRIIR-VIRDQLSNKN---------VTIQYGGSVKPNNIDEIMAQSDIDGALVGGASLDPVNFAR-
Trichodesmium_erythraeum          VDVDQE-----NLVIAYEPIWAIGTGDTCEAKEANRVIG-LIRGKLNNSN---------VTIQYGGSVKPNNVDDIMAQEEIDGALVGGASLNPESFSR-
Synechococcus_sp                  VDINQF-----NLVIAYEPIWAIGTGDTCAADEADAVIG-KIRALLDNKE---------VSIQYGGSVKPENVDEIMAQSEIDGALVGGASLTPDSFAR-
Prochlorococcus_marinus           EETDKK-----KLIVAYEPIWAIGTGKTCEANEANRICG-LIRQWANCPE---------ILIQYGGSVKPGNIDEIMAMSDIDGVLVGGASLDPESFAR-
Sphaerobacter_thermophilus        ADVEAAD--VLRTVIAYEPVWAIGTGRAATPEDAR-EMANWIREVLARHVG--AEAANEIRVQYGGSVNADNAAGFLSLPEIDGALVGGASLKADQFTQ-
Anaerolinea_thermophila           KDLLPEE--GLKLVIAYEPVWAIGTGRAATAEVAQSVHANVVRPALAHLFG--QEVAGQIRIQYGGSVTAKNAPELFAMPDIDGALVGGASLKAGEFAA-
Myxococcus_xanthus                EGFGAKE--VAGFVLAYEPVWAIGTGRNATAAQAQEVHA-AIRGLVERLYD--GETAGRVRIQYGGSVKPDNAAELLGQPDVDGALVGGASLKAGDFAA-
Collinsella_tanakaei              EGLEIAD--PAQLVVAYEPIWAIGTGMTATADDAQEVCA-AVREALADLFG--AELASGIRVLYGGSAKPGNIAELTAKPDVDGALVGGASLKAADFSQ-
Atopobium_vaginae                 KGLTLTS--PSQYVVAYEPIWAIGTGKTATAEDAQEVCS-AIRATLRDIFG--AELANGIRVLYGGSAKPENIAGFLAEEDVDGALVGGASLKADSFAA-
Eggerthella_sp                    AGIDATD--AKVCVVAYEPIWAIGTGRTATPEQAEAVCA-AIRATLAELYG--ADVSEAMRVLYGGSMNPGNVEGLMAQPNIDGGLVGGASLDATSFAE-
Dictyoglomus_thermophilum         EGLEKEN--VEKIVIAYEPVWAIGTGHSAKGEDANEVAG-LIRKIISEMYD--TEVSQKIRIQYGGSVNPQNITEFLSQNEIDGALVGGASLKPQSFWN-
Aquifex_aeolicus                  TGVE-EH--TDKIDIAYEPVWAIGTGTPATPEDAVEVHT-FIRNLINQLNP--KNE-GKTRILYGGSVNPQNAKEFMKHEEINGLLVGTASLDPESFAK-
Thermodesulfovibrio_yellowston    LEIN--S--PEALTIAYEPVWAIGTGVVATEEQIKQSHL-FIRNQLKEIYG--ERA-NEVRILYGGSVTPENIKSIMAIDNVEGVLVGGASLDPLKFAK-
Dehalogenimonas_lykanthroporep    DGVN-----SPDIYVAYEPVWAIGTGRAATGDIANETAM-LIRNIVGKLYG--NAVSQALPVLYGGSVNPENISDFISRSDIDGALVGGASIKAGQFVE-
Dehalococcoides_sp                DGLN-----LSCIIIAYEPIWAIGTGKAATASEANSAIG-YIRRVLGDTLG--NAAAQTSPILYGGSVNEKNITEILSQTNIDGALVGGASLKAESFVS-
Geobacter_metallireducens         AGLARGD--LAKIVIAYEPVWAIGTGKTATDEQAQIAHA-FIRKVVGELFT--VQIAETIRILYGGSVKPENVRGLMNQPDIDGALVGGASLKADSFGA-
Desulfobacter_postgatei           KGFDLGD--LDTLILAYEPVWAIGTGKTAGPEQVKEVHG-FLRHLLKEKYA--EGLAATIRILYGGSVKPGNIKDLMQLEDVDGALVGGASLNPEDFNE-
Planctomyces_brasiliensis         KDISAEQ--MTNVVIAYEPVWAIGTGKTASPEQAEQAHA-HLRKWLADRYT--SEVAEQTRILYGGSVKPANAKELLGQQNVDGALVGGASLTVDNFGP-
Spirochaeta_coccoides             GGVSSAD--MSRVVIAYEPVWAIGTGKTATPDDADNAHA-YIRGLINGLYG--KDVAEQLIIQYGGSVKADNVKELMAKPNVDGALVGGASLSIDKFLP-
Denitrovibrio_acetiphilus         KDIGMDR--INRVTLAYEPVWAIGTGKTATPADAEDIHG-KIRTQLSKMYG--PVVAEKIRILYGGSVKPDNVSALMMRENIDGALVGGASLDATSFAK-
Prosthecochloris_aestuarii        EGV--AD--IGDIVIAYEPVWAIGTGKTASSLQAQEVHA-SIRATVAGLYS--EPAAAGVRIQYGGSVKPSNAEELFAMPDIDGGLIGGASLNAEDFVA-
Chlorobium_phaeobacteroides       ADI--ED--LGNVVLAYEPVWAIGTGKTATPEQADAVHA-SIRATISDMYG--EADAEDIRIQYGGSVKPSNAVELFGMPNIDGGLIGGASLKADDFVA-
Chlorobium_ferrooxidans           AGI--SD--ISTVVIAYEPVWAIGTGKTASSAQAEEVHL-LIRNTITKLYG--DAAAGKLRIQYGGSVKPSNAAELFAMPNIDGGLIGGASLNAADFAA-
Chloroherpeton_thalassium         KDLTAED--MKSVVIAYEPVWAIGTGKTATPEQAQEVHA-FIRGIVKDMFG--EEVANELRIQYGGSVKTSNAKELFGMPDIDGGLIGGASLNAEDFVE-
Treponema_pallidum                ADVCGSL--MHNVTVAYEPVWAIGTGKTATPAQANAVHA-HIRSVVREMYG--AAIAEALCIQYGGSMKAENARALLAEEHIDGGLIGGASLEAASFVP-
Leptospira_interrogans            KGIDSVF--FSNLILAYEPVWAIGTGKVATPSQAQEVHS-FIRKEISGLFVGASSISESISILYGGSVKPDNIQDLLKEKDIDGGLVGGASQKISSFAE-
Borrelia_turicatae                ISVSESD--LKRIILAYEPVWAIGTGKTATKEEAQEVHR-AIRHEVELLYS--VSAANNIIIQYGGSVNIDNVKGLMGESDIDGALIGGASLKADSFLN-
Thermotoga_maritima               YGLDKEE--AKRVVIAYEPVWAIGTGRVATPQQAQEVHA-FIRKLLSEMYD--EETAGSIRILYGGSIKPDNFLGLIVQKDIDGGLVGGASLK-ESFIE-
Fusobacterium_nucleatum           VDLSKED--AEKTIVAYEPVWAIGTGKTATPEMAQETHK-EIRNVLAEMFG--KDVADKMIIQYGGSMKPENAKDLLSQEDIDGGLVGGASLKADSFFE-
Selenomonas_sputigena             DGFSADE--AAQLVIAYEPIWAIGTGKTATFEQAEEVCA-AIRATVAEVFN--KDAAEGIRIQYGGSVKPATIAGLMEKPNVDGALVGGASLKAKDFSE-
Ruminococcus_sp                   QGVTADQ--AKTAVIAYEPIWAIGTGKTATTEQAQEVCA-GIRACIAEIYD--EATAEAIRIQYGGSVNPATAPDLFVQNDIDGGLVGGASLKA-DFGK-
Clostridium_botulinum             KELSKEQ--VEKLVIAYEPIWAIGTGKTATDEQANETIG-YIRTVVKAMYG--ESVADKIRIQYGGSVKPGTIKAQMAKEEIDGALVGGASLKAEDFAA-
Clostridium_perfringens           EGLTKEQ--AEKVVIAYEPIWAIGTGKTATSDQANETIA-AIRAMVAEMFG--QEVADKVRIQYGGSVKPNTIAEQMAKSDIDGALVGGASLVAADFAQ-
Thermoanaerobacter_wiegelii       KGVSHDD--IVNVVIAYEPIWAIGTGKTATAKDANEVIK-AIRNTIASLYG--KGKASLVRIQYGGSVKPENISELMAESDIDGALVGGASLVASDFAK-
Brachyspira_murdochii             KGLSEAE--AKKVVIAYEPVWAIGTGKTATPQDADEVHK-TIRETLKSLYN--ESVAEGMIILYGGSVNEKNADDLLNMPNIDGALVGGASLVADKFAR-
Acholeplasma_laidlawii            EGVSKEQ--ALKTIVAYEPVWAIGTGKTATPQMANDTIK-DVRAKLSDLYG--AEVAAQVRILYGGSVKPDNIESLLQESDIDGALIGGAALDHKNFLT-
Lactobacillus_reuteri             RGVSLDQ--IKNVVVSYEPSWAVGYGQHANPVLAEEGCR-QIRRTIADNYT--YEIADKIRILYGGSVNPDNIGMIMNKPDVDGVLIGRASLDVDNFLR-
Bacillus_subtilis                 AGLSEEQ--VAASVIAYEPIWAIGTGKSSTAKDANDVCA-HIRKTVAESFS--QEAADKLRIQYGGSVKPANIKEYMAESDIDGALVGGASLEPQSFVQ-
```

```
Bacillus_thuringiensis             AGLTEEQ--VKATVIAYEPIWAIGTGKSSSSADANEVCA-HIRKVVAEAVS--PEAAEAVRIQYGGSVKPENIKEYMAQSDIDGALVGGASLEPASFLG-
Listeria_monocytogenes             AGLTEEQ--VIKSVIAYEPIWAIGTGKSSTSADANETCA-VIRAEVADAVS--QKAADAVRIQYGGSVKPENIADYLAESDIDGALVGGASLEPASFLA-
Geobacillus_stearothermophilus     AGLTPEQ--VKQAVIAYEPIWAIGTGKSSTPEDANSVCG-HIRSVVSRLFG--PEAAEAIRIQYGGSVKPDNIRDFLAQQQIDGPLVGGASLEPASFLQ-
Staphylococcus_aureus              AGLSEDQ--LKSVVIAYEPIWAIGTGKSSTSEDANEMCA-FVRQTIADLSS--KEVSEATRIQYGGSVKPNNIKEYMAQTDIDGALVGGASLKVEDFVQ-
Enterococcus_faecalis              VGLSNEQ--VASMVIAYEPIWAIGTGKSADANIADEICG-VVRSTVEKLYG--KEVSEAVRIQYGGSVKPENIAEYMAKENVDGALVGGASLEADSFLA-
Lactococcus_lactis                 AGLTAEQ--VSNLVIAYEPIWAIGTGKTATNEIADETCG-VVRSTVEKLYG--KEVSEAVRIQYGGSVKPETIEGLMAKENIDGALVGGASLEADSFLA-
Streptococcus_pneumoniae           AGLTAEQ--VAASVIAYEPIWAIGTGKSASQDDAQKMCK-VVRDVVAADFG--QEVADKVRVQYGGSVKPENVASYMACPDVDGALVGGASLEAESFLA-
Erysipelotrichaceae_bacterium      ADMCPKC--VGNMVIAYEPIWAIGTGKSASVEIAENCCR-IVRDQVRVMYG--DEAAENVRVQYGGSVKPNNIVEYMAQPDIDGALIGGASLKADSFIE-
Lentisphaera_araneosa              ADITAEQ--MTNLVVAYEPVWAIGTGVTASPEQAQDTHA-YIRSEFAALYG--QEVADKVIIQYGGSVKPANVAELMSCEDIDGALVGGASLKADDFSA-
Chlamydophila_pneumoniae           EQMDN----GSEFLIAYEPVWAIGTGKVAEASDVQDIHM-FCREVVAERFS--EATAEEISILYGGSVKVDNAQRFGQCSDVDGLLVGGASLEGQSFFE-
Desulfovibrio_vulgaris             ADVDATYV-PQSLVVAYEPVWAIGTGKVAGPAEVVEAHA-LVRSLLEARYG--RD-GAAIRILYGGSVKPDNAAELLSLDNVDGLLVGGASLQAVSFSR-
Mesorhizobium_opportunistum        QFFEEEVK-GATILFAYEPVWAIGDKGIPASSDYADKQ----QGLIKAVAG--SLLPSVPSVLYGGSVNPGNAAELIGQPNVDGLFIGRSAWQAQGYID-
Bartonella_henselae                P---DGAT-AENIIIAYEPVWAVGTGNTATSADVAEVHA-FIHHKMHSRF---GDEGAKIRLLYGGSVKPSNAFELLSTAHVNGALIGGASLKAIDFLT-
Brucella_abortus                   P---DGVT-AENTIIAYEPVWAIGTGLTPTVQDVRAAHA-FMREQLIERF---GAKGAHLRLLYGGSVKPSNAAELLGVADVDGALVGGASLKAADFLA-
Rhizobium_etli                     P---DGAT-AENTVIAYEPIWAIGTGVTPTSGDVEKAHA-FMRAEMAARF---GEEGRKMRLLYGGSVKPANAGELMGIANVDGALIGGASLKAADFLA-
Agrobacterium_tumefaciens          P---DEAT-AENTVIAYEPVWAIGTGLTPTTQDVREAHA-FMRDELVKRF---GDAGKTMRILYGGSVKPANALELMGVENVDGALIGGASLKAADFLS-
Rhodobacter_sphaeroides            P---DAAT-AANTVIAYEPVWAIGTGRTPTTAEIAEVHA-FLRARLAERF---SDA-EGFRLLYGGSVKPSNAAEIFAVPNVDGALVGGASLKAADFGG-
Ruegeria_sp                        P---DGVT-AENTVVAYEPVWAIGTGKVPTLDQIAEVHD-ALRADLVARF---GAAGKDLPLLYGGSVKPGNAAEIFGVSNVDGALVGGASLKAADFGP-
Bradyrhizobium_japonicum           P---DGST-AANLVVAYEPVWAIGTGLTPTVQDVEQIHG-FIREFLTSRF---SVDGAKMRILYGGSVKPSNAAELMAVKNVNGALVGGASLKAADFLA-
Rhodopseudomonas_palustris         P---DGAT-AANLVVAYEPVWAIGTGMTPTAADVEEVHG-FIRQTLTQRF---NAEGDQMRLLYGGSVKPSNARELMSVPHVNGALVGGASLKASDFLA-
Methylobacterium_radiotolerans     P---KGAT-AADTVIAYEPVWAIGSGRTPTPRDIAEVHA-SLREMLDKLV---GDEAQKIRILYGGSVKPGNAKELLSVDNVDGALVGGASLVAEDFLG-
Rhodospirillum_rubrum              P---THAD-SGKVVIAYEPVWAIGTGRVPTTEDVAKVHG-ALRKDLVGAL---GADGNGVRLLYGGSVKPDNAAELMALENVDGALVGGASLKAEDFWA-
Zymomonas_mobilis                  P---PDAT-SDNLVVAYEPIWAIGTGNTPPPESIAEMHA-SIRDRLCLLL---GSEGKKVRILYGGSVTGKNAEELMAIPDVNGTLVGGASLTAEQFVP-
Gluconacetobacter_hansenii         P---QGFT-G---IVAYEPVWAIGSGTAASQQDIADMTQ-FIREELVRQF---GDAGKTIKILYGGSVNGRNAADILPIADVGGALVGNASLAAETFMP-
Thermus_thermophilus               EG-VEPPG-PEALVIAYEPVWAIGTGKNATPEDAEAMHQ-EIRKALSERYG--EAFASRVRILYGGSVNPKNFADLLSMPNVDGGLVGGASLELESFLA-
Thermus_aquaticus                  KG-VEPES-PDRLVIAYEPVWAIGTGKNATPEDAEAMHQ-AIRQALAERYG--EAFASRVRVLYGGSVNPKNFADLLSMPNVDGGLVGGASLELESFLA-
Oceanithermus_profundus            EG-VGLET-GEELVIAYEPVWAIGTGKTASADDAQAMAA-AIRGFLAERYG--ESVAGRTRILYGGSMKPANTTEILAGPDVDGGLVGGASLEVASFSA-
Deinococcus_deserti                EG-VG-----TDVVIAYEPVWAIGTGKTATAEDAEELAE-AIRGALTEQYG--EAAG-TLRILYGGSVKPDNIASICAKPNVNGALVGGASLKVADVVG-
Bifidobacterium_bifidum            RD-LSDKE-AAKLIIAYEPVWAIGTGMVATPDTAQQAAK-AIRDDLGEMFG--PAVADTVRILYGGSVSSKNATHLIGEPDVDGFLIGGASLDPDELAK-
Bifidobacterium_longum             RD-LNEEQ-AAKLIVAYEPVWAIGTGMVATPQSAQDAAN-AIRNDLKTTFG--TKVSDSVRILYGGSVTSKNAAELISQPDVDGFLIGGAALDVEELAK-
Gardnerella_vaginalis              RD-LDSKQ-AERLIIAYEPVWAIGTGMVATAQSAQDAAK-AIRDDLQETFG--DTVSQKVRILYGGSVTSSNAASLICEPDVDGFLIGGASLDIEELAT-
Nocardia_farcinica                 KG-LSAEQ-IAKVVIAYEPVWAIGTGKVASAADAQEVCG-AIRAELAELAG--PEVAAQVRVLYGGSVNAKNVGELVAQPDVDGALVGGASLKGDEFAT-
Mycobacterium_tuberculosis         AG-LLAEQ-IGSVVIAYEPVWAIGTGRVASAADAQEVCA-AIRKELASLAS--PRIADTVRVLYGGSVNAKNVGDIVAQDDVDGGLVGGASLDGEHFAT-
Corynebacterium_glutamicum         AG-LDAAE-LANTVIAYEPVWAIGTGKVASAADAQEVCK-AIRGLIVELAG--DEVAEGLRILYGGSVKAETVAEIVGQPDVDGGLVGGASLDGEAFAK-
Propionibacterium_acnes            DG-IPAEQ-VAKLVIAYEPIWAIGTGETATADDAQEVCQ-AIREAVKELYD--APTAEAVRIQYGGSVKPANVAEIMAKPDVDGALVGGASLKAGDFSK-
Actinomyces_odontolyticus          AG-WKPEE-VAKIVIAYEPIWAIGTGETASAEDAQEVCG-AIRAALAEDFG--AETAESTRILYGGSAKPDNIKELMAQPDVDGGLVGGASLKADSFAA-
Microbacterium_testaceum           EG-VAS---AAEIVVAYEPVWAIGSGQAATPEQAQEVCA-KLRAVVAEKLG--ADAAARTRVLYGGSVKANNIAAFMREPDVDGALVGGASLVVDEFAA-
Moritella_marina                   NT-QGVEA-LEGAIIAYEPIWAIGTGKAATAEDAQRIHA-QIR-AHIAEKS--EAVAKNVVIQYGGSVKPENAAAYFAQPDIDGALVGGAALDAKSFAA-
Photobacterium_profundum           NT-QGVEA-LEGAIIAYEPIWAIGTGKAATAEDAQRIHA-QIR-AHIAEKS--EEVAKNVVIQYGGSVKPENAAAYFAQPDIDGALVGGAALDAKSFAA-
Vibrio_cholerae                    DA-YGVEA-LNGAIIAYEPIWAIGTGKAATADDAQRIHA-SIR-ALIAAKD--AAVAEQVIIQYGGSVKPENAASYFAQPDIDGALVGGASLDAKGFAA-
Shigella_flexneri                  KT-QGAAA-FEGAVIAYEPVWAIGTGKSATPAQAQAVHK-FIR-DHIAKVD--ANIAEQVIIQYGGSVNASNAAELFAQPDIDGALVGGASLKADAFAV-
Escherichia_coli                   KT-QGAAA-FEGAVIAYEPVWAIGTGKSATPAQAQAVHK-FIR-DHIAKVD--ANIAEQVIIQYGGSVNASNAAELFAQPDIDGALVGGASLKADAFAV-
Salmonella_enterica                KT-QGAAA-FEGAVIAYEPVWAIGTGKSATPAQAQAVHK-FIR-DHIAKAD--AKIAEQVIIQYGGSVNASNAAELFAQPDIDGALVGGASLKADAFAV-
Klebsiella_pneumoniae              KT-QGAAA-FEGVVIAYEPVWAIGTGKSATPAQAQAVHK-FIR-DHIAKAD--AKIAEQVIIQYGGSVNAGNAAELFTQPDIDGALVGGASLKADAFAV-
Enterobacter_aerogenes             KT-QGAAA-FEGVVIAYEPVWAIGTGKSATPAQAQAVHK-FIR-DHIAKAD--AKIAEQVIIQYGGSVNAGNAAELFTQPDIDGALVGGASLKADAFAV-
Serratia_symbiotica                KT-LGAPA-MKSAVIAYEPVWAIGTGKSATPAQAQAVHK-FIR-DHIAKHD--TAIAAEVIIQYGGSVNDKNAAELFAQPDIDGALVGGASLKADAFAA-
```

```
Yersinia_pestis                      NT-LGVKA-FEGAVIAYEPIWAIGTGKSATPAQAQAVHK-FIR-DHIAKQD--AAVAAQVIIQYGGSVNDKNAAELFTQPDIDGALVGGASLKADAFAV-
Actinobacillus_pleuropneumonia       DA-LGVEA-FNGAVIAYEPIWAIGTGKSATPAQAQAVHA-FIR-GHIAAKS--QAVADQVIIQYGGSVNDANAAELFTQPDIDGALVGGASLKAPAFAV-
Pseudomonas_aeruginosa               DE-LGVGA-FARAVVAYEPVWAIGTGLTASPAQAQEVHA-AIR-AQLAAEN--AEVAKGVRLLYGGSVKAASAAELFGMPDIDGGLVGGASLNADEFGA-
Marinomonas_posidonica               DV-VGIQA-FENIVIAYEPVWAIGTGLSASAQQAQDVHQ-AIR-ANLASLS--TPVSEKVQILYGGSVKASTSAELFTMPDVDGALVGGASLDAKEFLD-
Xylella_fastidiosa                   AL-VGPQG-VARGLIAYEPVWAIGTGRHADPSQVQAMHA-FIR-GEIARQD--ARIGDSLLILYGGGIKPCNAAELFSQQDVDGGLIGGASLVADDFLA-
Xanthomonas_campestris               SL-VGSAG-FARAVVAYEPIWAIGTGRTATPDQAQAVHA-FIR-GEVAKAD--ARIADSLPILYGGSVKPDNASELFSQPDVDGGLVGGASLVAEDFLA-
Marinobacter_algicola                AD-VGNGL-WQQVVIAYEPVWAIGTGKTATADDAQAMHA-AIR-AVLMSLG--A-PADEVSLLYGGSVKADNAAALFAQPDIDGGLIGGASLDASDFVS-
Aeromonas_caviae                     EK-NGAMA-FDNAIIAYEPIWAVGTGKCATPEQAQEVHS-FIR-SRLAEDT--PVIGEKVRIIYGGSVTPANAQDLFAQSDIDGGLIGRASLDGEAFLG-
Pseudoalteromonas_haloplanktis       EK-LGVAA-LKDSVIAYEPVWAIGTGKTASPEQAQSVHK-FIR-DKIASLN--SDLAQGLTILYGGSVNEKNSELLFAQTDIDGGLIGGASLKADSFTA-
Legionella_pneumophila               AK--GKDC-FRDCVVAYEPVWAIGTGKTATPEQAQKIHQ-FIR-DLVGEIN--DSDAKHLTLIYGGSVNENNAKALFSMPDIDGGLVGGASLNAKQFVE-
Ralstonia_solanacearum               ES-LSLEQ-LGRIVVAYEPVWAIGTGKTATSEQAQAVHA-FLR-GRVAACD--AGVAQRMPILYGGSVKPDNAAELFSMADIDGGLIGGASLKAEDFLV-
Burkholderia_sp                      AK-LSAQE-AARLVVAYEPVWAIGTGKSASAAQAQAVHA-FLR-ARLAAKG-----AADVPLLYGGSVKPDNAEELFRQNDIDGGLIGGASLKDQDFLA-
Bordetella_pertussis                 A--LGRDA-LVRMVLAYEPVWAIGTGRTASPEQAQEVHS-AIR-VALDGLQ-----ASQVRVLYGGSVKGANAASLFAMPDIDGGLVGGASLVAEEFLR-
Nitrosococcus_watsonii               QL-AGVNA-LKKAVIAYEPIWAIGTGRTATPEQAQEVHA-FIR-GHVAVQN--SGIAEELLVLYGGSVKGSNAAQLLAMPDIDGGLIGGASLNAKEFLT-
Coxiella_burnetii                    QMNDNLAS-LEGMVVAYEPIWAIGTGKNATPSQAEEVHA-ALR-DQLHRQD--ATLAESTRLLYGGSVKPDNAAALFEMPNIDGALVGGASLEAEQFLK-
Variovorax_paradoxus                 HV--NGHC-ISEIVVAYEPVWAIGTGKTASPEQAQAVHA-VLR-AQLHHAA--SEHAAGISILYGGSMNAANAAELLAQADIDGGLIGGASLKAPDFLQ-
Halomonas_elongata                   DG-LEAGQ-RSRLVIAYEPVWAIGTGRTATPEQAQAIHA-AIR-RRLARYG--DGLAEGTQLLYGGSMKADNAADLLAQPDIDGGLVGGASLKIDDFLA-
Tenebrio_Molitor                     AKVND----WSNVVIAYEPVWAIGTGKTATPQQAQDVHK-ALRQWICENIDA--KVGNSIRIQYGGSVTAANCKELASQPDIDGFLVGGASLK-PEFVD-
Bombyx_mori                          SAIGDK---WENIVLAYEPVWAIGTGKTATPQQAQDVHH-ALRNWLSANVSG--SVSDAVRIQYGGSVTAANAKELASCKDIDGFLVGGASLK-PEFVE-
Apis_mellifera                       NKINS----WDNVVVAYEPVWAIGTGKTATPQQAQEVHE-KLRNWFSKNVNQ--TVAETVRIIYGGSVTAGNAKDLAKEKDIDGFLVGGASLK-PDFVQ-
Aedes_aegypti                        DKVKD----WSNVVIAYEPVWAIGTGKTATPEQAQEVHA-ALRKWFTENVSA--DVSGAIRIQYGGSVTAANCRELAAKPDIDGFLVGGASLK-PEFIQ-
Anopheles_gambiae                    AQVKD----WSNVVIAYEPVWAIGTGKTATPEQAQEVHA-ALRKWFTENVSA--DVSNAIRIQYGGSVTAANCRELAAKPDIDGFLVGGASLK-PEFIQ-
Drosophila_melanogaster              QKIKD----WKNVVVAYEPVWAIGTGQTATPDQAQEVHA-FLRQWLSDNISK--EVSASLRIQYGGSVTAANAKELAKKPDIDGFLVGGASLK-PEFVD-
Rhipicephalus_microplus              SNVKD----WSKVVIAYEPVWAIGTGKTATPDQAQEVHS-KVRNWLSTNVSA--DVASKVRIQYGGSVNAGNCKELGRKPDIDGFLVGGASLK-PEFVQ-
Ixodes_scapularis                    AKVTD----WNRVVLAYEPVWAIGTGKTASPEQA-EVHA-QLRQWLSKNVSP--DVAKKVRIQYGGSVTAANCQELAKKPDVDGFLVGGASLK-PEFVE-
Anolis_carolinensis                  DNVKD----WSKVVLAYEPVWAIGTGKTATPQQAQEVHL-KLRGWLKSHVSD--AVSQSTRIIYGGSVTGATAKELASQPDLDGFLVGGASLK-PEFVD-
Gallus_gallus                        DNVKD----WSKVVLAYEPVWAIGTGKTATPQQAQEVHE-KLRGWLKSHVSD--AVAQSTRIIYGGSVTGGNCKELASQHDVDGFLVGGASLK-PEFVD-
Orcinus_orca                         DNVKD----WGKVVLAYEPVWAIGTGKTATPQQAQEVHE-KLRGWLKSNISD--AVAQSTRIIYGGSVTGATCKELASQPDVDGFLVGGASLK-PEFVD-
Bos_taurus                           DNVKD----WSKVVLAYEPVWAIGTGKTATPQQAQEVHE-KLRGWLKSNVSD--AVAQSARIIYGGSVTGATCKELASQPDVDGFLVGGASLK-PEFVD-
Canis_lupusfamiliaris                DNVKD----WSKVVLAYEPVWAIGTGKTATPQQAQEVHE-KLRGWLKSNVSD--AVAQSTRIIYGGSVTGATCKELASQPDVDGFLVGGASLK-PEFVD-
Odobenus_rosmarus                    DNVKD----WSKVVLAYEPVWAIGTGKTATPQQAQEVHE-KLRGWLKSNVSD--AVAQSTRIIYGGSVTGATCKELASQPDVDGFLVGGASLK-PEFVD-
Oryctolagus_cuniculus                DNVKD----WSKVVLAYEPVWAIGTGKTATPQQAQEVHE-KLRGWLKSNVSD--AVAQSTRIIYGGSVTGATCKELASQPDVDGFLVGGASLK-PEFVD-
Nomascus_leucogenys                  DNVKD----WSKVVLAYEPVWAIGTGKTATPQQAQEVHE-KLRGWLKSNVSD--AVAQSTRIIYGGSVTGATCKELASQPDVDGFLVGGASLK-PEFVD-
Pan_troglodytes                      DNVKD----WSKVVLAYEPVWAIGTGKTATPQQAQEVHE-KLRGWLKSNVSD--AVAQSTRIIYGGSVTGATCKELASQPDVDGFLVGGASLK-PEFVD-
Homo_sapiens                         DNVKD----WSKVVLAYEPVWAIGTGKTATPQQAQEVHE-KLRGWLKSNVSD--AVAQSTRIIYGGSVTGATCKELASQPDVDGFLVGGASLK-PEFVD-
Pongo_abelii                         DNVKD----WSKVVLAYEPVWAIGTGKTATPQQAQEVHE-KLRGWLKSNVSD--AVAQSTRIIYGGSVTGATCKELASQPDVDGFLVGGASLK-PEFVD-
Macaca_mulatta                       DNVKD----WSKVVLAYEPVWAIGTGKTATPQQAQEVHE-KLRGWLKSNVSE--AVAQSTRIIYGGSVTGATCKELASQPDVDGFLVGGASLK-PEFVD-
Ochotona_princeps                    DNVKD----WSKVVLAYEPVWAIGTGKTATPQQAQEVHE-KLRGWLKSNVSD--AVAQSTRIIYGGSVTGATCKELASQADVDGFLVGGASLK-PEFVD-
Sus_scrofa                           DNVKD----WNKVVLAYEPVWAIGTGKTATPQQAQEVHE-KLRGWLKTHVPE--AVAHSTRIIYGGSVTGATCKELASQPDVDGFRVSGASLK-PEFVD-
Mus_musculus                         DNVKD----WSKVVLAYEPVWAIGTGKTATPQQAQEVHE-KLRGWLKSNVND--GVAQSTRIIYGGSVTGATCKELATPADVDGFLVGGASLK-PEFVD-
Rattus_norvegicus                    DNVKD----WCKVVLAYEPVWAIGTGKTATPQQAQEVHE-KLRGWLKCNVSE--GVAQCTRIIYGGSVTGATCKELASQPDVDGFLVGGASLK-PEFVD-
Xenopus_laevis                       DNVKD----WSKVVLAYEPVWAIGTGKTATPEQAQEVHK-KLREWVKTNVSE--GVAQSVRIIYGGSVTGGTCRELAGQPDIDGFLVGGASLK-PEFIE-
Xenopus_tropicalis                   DNVKD----WSKVVLAYEPVWAIGTGKTATPEQAQEVHK-KLREWLKTNVSE--DVAKSVRIIYGGSVTGGTCKELGAQPDIDGFLVGGASLK-PEFID-
Danio_rerio                          DNVKD----WSKVVLAYEPVWAIGTGKTASPQQAQEVHD-KLRQWLKTNVSE--AVANSVRIIYGGSVTGGTCKELASQKDLDGFLVGGASLK-PEFID-
Ictalurus_punctatus                  DNVKD----WSKVVVAYEPVWAIGTGKTASPQQAQEVHD-KLRQWMSANISE--AVANSVRIIYGGSVTGATCKELASQKDVDGFLVGGASLK-PEFID-
Oreochromis_niloticus                DNVKD----WSKVVLAYEPVWAIGTGKTASPQQAQEVHD-KLREWLKTNVSE--AVANSVRIIYGGSVTGGTCKELASQKDVDGFLVGGASLK-PEFID-
```

```
Nematostella_vectensis           DKIS----DWSKVVIAYEPVWAIGTGKTATPQQAQEVHN-QLRAWLKENVSV--DVAESTRIIYGGSVNAKNCRELAAEGDIDGFLVGGASLK-PEFVQ-
Schistosoma_mansoni              NKIKSAD-EWKRVVVAYEPVWAIGTGKVATPQQAQEVHN-FLRKWFKTNAPN--GVDEKIRIIYGGSVTAANCKELAQQHDVDGFLVGGASLK-PEFTE-
Strongylocentrotus_purpuratus    DVITD----WGNVVIAYEPVWAIGTGKTATPQQAQEVHQ-NLRSWLTEHVNA--AVAANTRIVYGGSVNGGNCCDIGQQPDVDGFLVGGASLK-PEFKQ-
Caenorhabditis_elegans           DKGVS----WENIVIAYEPVWAIGTGKTASGEQAQEVHE-WIRAFLKEKVSP--AVADATRIIYGGSVTADNAAELGKKPDIDGFLVGGASLK-PDFVK-
Caenorhabditis_remanei           DKGVS----WDNIVVAYEPVWAIGTGKTASPEQAQEVHE-WIREFLKEKVSP--AVADSTRIIYGGSVTADNAAELGKKADIDGFLVGGASLK-PDFVK-
Brugia_malayi                    DKKAN----WNKIVIAYEPVWAIGTGKTASPEQAQEVHG-WIREFLKEKVSA--DVAQKTRILYGGSVTAENAAELAKKPDIDGFLVGGASLK-PDFIK-
Trichinella_spiralis             KYKLD----WNNIVIAYEPVWAIGTGKTASPEQAQEVHV-WIRDWLKQNVSA--DAAAKCRIIYGGSVTGDNCRALGKQSDIDGFLVGGASLK-PEFLK-
Ricinus_communis                 ERVKD----WADVVLAYEPVWAIGTGKVATPAQAQEVHF-ELRKWLKENTSS--QVAATTRIIYGGSVTATNCKELAAQPDVDGFLVGGASLK-PEFIE-
Vitis_vinifera                   DKVSN----WANVVLAYEPVWAIGTGKVATPAQAQEVHS-ELRNWFQANASP--EVAATIRIIYGGSVSGANCKELAAKPDVDGFLVGGASLK-PEFID-
Arabidopsis_thaliana             DRVTN----WSNVVIAYEPVWAIGTGKVASPAQAQEVHD-ELRKWLAKNVSA--DVAATTRIIYGGSVNGGNCKELGGQADVDGFLVGGASLK-PEFID-
Zea_mays                         EKIKD----WSNVVVAYEPVWAIGTGKVATPAQAQEVHA-SLRDWLKTNASP--EVAESTRIIYGGSVTAANCKELAAQPDVDGFLVGGASLK-PEFID-
Oryza_sativa                     ERIKD----WTNVVVAYEPVWAIGTGKVATPDQAQEVHD-GLRKWLAANVSA--EVAESTRIIYGGSVTGANCKELAAKPDVDGFLVGGASLK-PEFID-
Fragaria_vesca                   EKVKD----WTNIVLAYEPVWAIGTGKVASPAQAQEVHC-ELRKWLHANTSP--EVAASTRIIYGGSVNGANSKELAGQPDLDGFLVGGASLK-PEFID-
Glycine_max                      AKISN----WDNVVLAYEPVWAIGTGKVATPAQAQEVHA-DLRKWVHDNVSA--EVAASVRIIYGGSVNGGNCKELAAQPDVDGFLVGGASLK-AEFVD-
Cucumis_sativus                  EKISN----WDNVVLAYEPVWAIGTGKVATPAQAQEVHA-ELRKWFQNNVSA--EVAGSVRIIYGGSVNGANCKELAAQPDLDGFLVGGASLK-VNSSY-
Perkinsus_marinus                PVVKD----WNNIVIAYEPVWAIGTGKVATTEQAQETHA-SIRAYLKSKVST--EVAENTRILYGGSVNPGNCGELIKCADVDGFLVGGASLK-PQFTE-
Aspergillus_niger                KELTK-E-QWAKVVIAYEPVWAIGTGKVATTAQAQEVHA-AIRKWLVDAISA--EAADNTRIIYGGSVSEKNCRDLAKEADVDGFLVGGASLK-PAFVD-
Penicillium_chrysogenum          KELSA-E-QWDKVVIAYEPVWAIGTGKVATTEQAQEVHA-AIRKWLGEAISA--QAQENVRVIYGGSVSEKNCRELATQPDVDGFLVGGASLK-PAFVD-
Paracoccidioides_brasiliensis    EEVSP-A-EWNKVVIAYEPIWAIGTGKVATTEQAQEVHA-SIRKWLNEKISP--EAAENIRVIYGGSVTENNCRDLAAQPDVDGFLVGGASLK-PAFVD-
Schizosaccharomyces_pombe        DKV---Q-NWSKIVIAYEPVWAIGTGKTATPEQAQEVHA-EIRKWATNKLGA--SVAEGLRVIYGGSVNGGNCKEFLKFHDIDGFLVGGASLK-PEFHN-
Saccharomyces_cerevisiae         EEVK----DWTNVVVAYEPVWAIGTGLAATPEDAQDIHA-SIRKFLASKLGD--KAASELRILYGGSANGSNAVTFKDKADVDGFLVGGASLK-PEFVD-
Kluyveromyces_lactis             EKVQ----DWTNVVVAYEPVWAIGTGLAATAEDAQDIHH-SIREFLAEKLSR--DVADSVRILYGGSANGKNAVTFKDKADVDGFLVGGASLK-PEFVD-
Candida_dubliniensis             KIVS----DWTNIVVAYEPVWAIGTGLAATPDDAEETHK-GIRAHLAKTIGA--EQAEKTRILYGGSVNGKNAKDFKDKANVDGFLVGGASLK-PEFVD-
Clavispora_lusitaniae            KIVS----DWTNIVVAYEPVWAIGTGLAATPQDAQDTHK-EIRAHLAKTIGA--EQAEKVRILYGGSVNGKNAPEFKDKVDVDGFLVGGASLK-PEFVD-
Chlamydomonas_reinhardtii        D-EVKD---WTKVVLAYEPVWAIGTGVVASPEQAQEVHA-YLRQYCAKKLGA--AVADKLRIIYGGSVSDTNCKDLSKQEDIDGFLVGGASLKGAAFVT-
Volvox_carteri                   D-EVKD---WSKVVIAYEPVWAIGTGVVATPEQAQEVHA-YLRRFCQSKLGT--SVADQLRIIYGGSVNDANCKELSVQEDIDGFLVGGASLKGSAFVT-
Dictyostelium_fasciculatum       NGVVSD---WSKVVIAYEPVWAIGTGKTASPQQAQDAHA-FIRKWLSEKVSK--EVANATRILYGGSVTATNSAELSKQLDVDGFLVGGASLVASDFVT-
Entamoeba_histolytica            NAISK-E-AWKNIILAYEPVWAIGTGKTATPDQAQEVHQ-YIRKWMTENISK--EVAEATRIQYGGSVNPANCNELAKKADIDGFLVGGASLDAAKFKT-
Cryptosporidium_parvum           KDVS--D--LSNLVIAYEPIWAIGTGVVATPGQAQEAHA-FIREYVTRMYNP--QVSSNLRIIYGGSVTPDNCNELIKCADIDGFLVGGASLK-PTFA--
Cryptosporidium_muris            KKVS--D--LSNIVIAYEPIWAIGTGVVATPEQAQEAHE-FIRKCLLNMYNN--NVSSSTRIIYGGSVNPENCNELINCADIDGFLVGGASLK-PSFV--
Plasmodium_falciparum            DLID--N--FDNVILAYEPLWAIGTGKTATPEQAQLVHK-EIRKIVKDTCGE--KQANQIRILYGGSVNTENCSSLIQQEDIDGFLVGNASLK-ESFV--
Plasmodium_vivax                 HLID--N--FDNVVLAYEPIWAIGTGKTATPEQAQEVHK-EIRNIVKEKCGE--KNANQIRILYGGSVNTDNCASLIKQEDIDGFLVGNASLK-SSFV--
Paramecium_tetraurelia           KELT--TALWSKVVVAYEPVWAIGTGRTATPEQAQEVHA-FIRGWLKSQIGT--QAEQATRIIYGGSVTEKNAADLIKQPDLDGFLVGGAALK-PGFA--
Tetrahymena_thermophila          GKLN--EAQWEKIVIAYEPVWAIGTGKVATPAQAQEVHE-FIRSYLTQKVSQ--NVSQKTRILYGGSVTEKNAGELIHQKDIDGFLVGGASLK-PAFA--
Toxoplasma_gondii                PKIS--D--WDRVVIAYEPVWAIGTGKVATPAQAQEVHE-HIREFLKAKVSE--DVANKVRIVYGGSVNASNSTELILQPDLDGFLVGGASLK-KDFL--
Trypanosoma_brucei               KKL--KKADWAKVVIAYEPVWAIGTGKVATPQQAQEAHA-LIRSWVSSKIGA--DVAGELRILYGGSVNGKNARTLYQQRDVNGFLVGGASLK-PEFV--
Trypanosoma_cruzi                QKL--SKEAWAHVVIAYEPVWAIGTGKVATPQQAQEVHE-LLRRWVRSKLGA--DIAAQLRILYGGSVTAKNARTLYQMRDINGFLVGGASLK-PEFV--
Leishmania_mexicana              AKL--TKDAWNQVVLAYEPVWAIGTGKVATPEQAQEVHL-LLRKWVSENIGT--DVAAKLRILYGGSVNAANAATLYAKPDINGFLVGGASLK-PEFR--
Leishmania_donovani              TKL--PKEAWDQIVLAYEPVWAIGTGKVATPEQAQEVHA-LLRKWVSEKIGA--DVAARLRILYGGSVSAGNAKTLYMKPDINGFLVGGASLK-PEFR--
Giardia_lamblia                  KELGESKMLWKEVVIAYEPVWSIGTGVVATPEQAEEVHV-GLRKWFAEKVCA--EGAQHIRIIYGGSANGSNCEKLGQCPNIDGFLVGGASLK-PEFMTM
Trichomonas_vaginalis            PAIPAG--KWDDVVIAYEPIWAIGTGKVASTQDAQEMCK-VIRDILAAKVGA--DIANKVRILYGGSVKPNNCNELAACPDVDGFLVGGASLE-PGFIN-
Sphingobacterium_sp              FHLSASD--FSKIILAYEPVWAIGTGLTASPEQAQEVHA-FIRKQIEAKYGA--AVADETSVLYGGSCNPKNAPDLFSQPDIDGGLIGGASLKSRDFTD-
Pedobacter_saltans               FHLSAED--FSKLVIAYEPVWAIGTGVTASSAQAQEIHA-FIRSEVAAKYGA--EVAENTTILYGGSCNPGNAAELFAQKDIDGGLIGGASLKSRDFVD-
Marivirga_tractuosa              FHLTPEQ--FKNIVIAYEPIWAIGTGKTASSQQAQDMHK-TIREHIAAKFGQ--DAANKTSILYGGSCKPDNAKELFSQPDVDGGLIGGASLKSRDFTD-
Cyclobacterium_marinum           FGFSEEE--IKKLVIAYEPIWAIGTGKTASSEQAQDMHA-AIRKHLSSKYGE--AVAEEISILYGGSCKPSNAKEIFSKADVDGGLIGGASLKSRDFVD-
Leadbetterella_byssophila        FHLSEEA--IQQVVIAYEPIWAIGTGVTASSDQAQEMHK-VLRDHLASKYGA--AVAEEISILYGGSVNAGNAEELFSQPDVDGGLVGGASLKSRDFTD-
```

```
Haliscomenobacter_hydrossis     FHLSPEE--FQKVVIAYEPIWAIGTGRTASPEQAQDMHA-AIRALLTDQYGA--EIADATTILYGGSVNGGNAAVLFSQPDVDGGLVGGASLKAEEFIT-
Parabacteroides_distasonis      FDLSAED--FGKIILAYEPVWAIGTGKTASAEQAEEIHA-HIRATLAAKYGN--EVADNCTILYGGSCNAGNAKELFAKPNVDGGLIGGASLAVDKFMP-
Bacteroides_fragilis            FDLSAED--FSKIVLAYEPVWAIGTGKTASPAQAQEIHA-FIRSAVAEKYGK--EIADNTSILYGGSCKPSNAKELFANPDVDGGLIGGAALKVADFKG-
Prevotella_ruminicola           FNLSEED--FRKIVIAYEPIWAIGTGKTATAEQAEEIHA-YIRSIIAEKYGQ--AVADDTTILYGGSCKASNAPELFAKPDIDGGLIGGASLKAADFKG-
Alistipes_shahii                YNLTEEQ--YKNLVIAYEPVWAIGTGKTASAEQAQEIHA-YIRQVLTAKFG---AAAQETAILYGGSCKPSNAAEIFAKEDVDGGLIGGAALKAEDFLA-
Porphyromonas_asaccharolytica   GHLSHEQ--MEQIVIAYEPVWAIGTGLTATPDQAQEMHQ-HIRQTVASLFDQ--KLADLTTILYGGSCKASNAESLFSQPDVDGGLIGGAALKADTFLP-
Cellulophaga_lytica             FNLKPEA--WSNIVLAYEPVWAIGTGETASPEQAQEMHA-FIRKTIADEYTT--EIAEAVSILYGGSVKPANAREIFSKADVDGGLIGGAALKADDFIA-
Kordia_algicida                 FHLDTAA--WKNIVLAYEPVWAIGTGETASPEQAQEMHA-FIRKTVSEKFGD--ETGNGVSILYGGSVKPANAAEIFAKEDVDGGLIGGASLKAADFVA-
Flavobacterium_psychrophilum    FHIDKSA--WANIILAYEPVWAIGTGETASPEQAQEMHE-FIRETVRKVFGS--DIAEDVSILYGGSVKPDNAKEIFSKPDVDGGLIGGAALKADDFVV-
Neisseria_gonorrhoeae           QGLDTKN--IA---VAYEPVWAIGTGKVATVEQIADMHA-FIYKEILSLCGS---DVK-IRVLYGGSVKADNAADIFAVPYVDGALVGGASLSYDSFTA-
Francisella_tularensis          ENLSVEQ--LAKVVIAYEPVWAIGTGVVASLEQIQETHQ-FIRSLLAKVDER---LAKNIKIVYGGSLKAENAKDILSLPDVDGGLIGGASLKAAEFNE-
Mycoplasma_genitalium           DTIDKSL--IKNLVIAYEPLWAIGTGKTATPEVANQTIK-TIREYINDLYDE--NVANNISILYGGSVDHNNIQKLAIMEQIDGFLVGKASLEIKNFLE-
Ureaplasma_urealyticum          VINYKG---LKKIIIAYEPIWAIGTNLTLDLKHINHMIE-GIKTYLYNCTGL--N----IPILYGGSVNANNIKELCTQKLIDGFLIGNASLDVNNFNQ-
Ureaplasma_parvum               IINYKG---LKKIIIAYEPIWAIGTDLELDFKHINYMIE-GIKTYLYNCTGI--N----IPILYGGSVNDNNINELCNQKLIDGFLIGNASLDVNVFNK-
Buchnera_aphidicola             ILKNLGTSAFKNIIIAYEPIWAIGTGVSADPEHVQLIHV-FIKNYILKYSSI--NR-NDIIIQYGGSINHTNVKKFIEQPDINGLLIGNSSLSAKEFLE-
Campylobacter_jejuni            IDLNYEK-----LIIAYEPIYSIGTGVSAQSTDIAKVLE-FLASLT------------KAPLLYGGSVNENNIKEILSVNHCGGVLIGSAALKVENFIK-
Helicobacter_pylori             IDLNYSN-----LIVAYEPIWAIGTKKSASLEDIYLTHG-FLKQILN----------QKTPLLYGGSVNIQNAKEILGIDSVDGLLIGSASWELENFKT-
Orientia_tsutsugamushi          ISFSFTKN----VIIAYEPIWSIGSDMIPTIDDIYEVVT-MIREIQNRYIPH--NIENSVKIVYGGSVSANNIHQILTAG-VDGVLIGKASLKLESLTT-
Wolbachia_sp                    R-LPTHGE----YTVAYEPIWAIGTGHVPNNDAIAKVIE-VIKLCTS-----------KKHIIYGGSVSSENIENLLNISNLSGVLIGSASLDFDHFYK-
Methanocaldococcus_Jannaschii   --------------IAVEPPELIGTGIPVSKANPEVVEG------TVRAVK---EINKDVKVLCGAGISKGEDVKAALDLGAEGVLLASGVVKAKNVEE-
Methanococcus_voltae            --------------IAVEPPELIGSGIPVSKANPEVVEG------TVSAVH---DINKDVKVLCGAGISKGEDVKSALELGAEGVLLASGVVKAKDVEQ-
Methanothermococcus_okinawensi  --------------IAIEPPELIGTGIPVSKANPEVVEG------TVKEVR---GINKDVKILCGAGISKGEDVSSALELGACGVLLASGVVKSKDVEG-
Pyrococcus_Woesei               --------------VAVEPPELIGTGIPVSKAKPEVITN------TVELVK---KVNPEVKVLCGAGISTGEDVKKAIELGTVGVLLASGVTKAKDPEK-
Pyrococcus_furiosus             --------------VAVEPPELIGTGIPVSKAKPEVITN------TVELVK---KVNPEVKVLCGAGISTGEDVKKAIELGTVGVLLASGVTKAKDPEK-
Thermofilum_pendens             --------------VAIEPPELIGTGIPVSKAKPEVVTS------SVELVK---KVNPNVKVLCGAGITVGEDVAAALRLGTVGVLLASGVVKAKDWEK-
Methanothermobacter_thermautot  --------------VAVEPPELIGSGIPVSRAEPEVITG------SVDAVK---KVNPEVSVLCGAGISTGDDMKAAVDLGAEGVLLASGIILADSPRD-
Methanosaeta_thermophila        --------------VAVEPPELIGSGIPVSKADPEVVRG------SVEAVR---AIEKDVGVLCGAGITHGDDLRAAIELGAVGVLLASGIVKAKDQRK-
Natronobacterium_gregoryi       --------------VAVEPPELIGTGTPVSQADPGVVEN------AVVAAE---NVDDDVSVLCGAGISTGEDVTAADDLGTEGVLLASGVAKADNPRA-
Halobacterium_salinarum         --------------VAVEPPALIGTGTPVSQADPDIVSD------AVAAAE---AVDPSVDVYCGAGITTGEDVVSAGDLGASGVLLASGVAKADDPRA-
Halogeometricum_borinquense     --------------VAVEPPELIGGDDSVATADPDIVRD------AVEAAA---NVDESVEVFCGAGISSGDDVDAAGDLGATGILLASGVAKADDPRA-
Methanosphaerula_palustris      --------------VAIEPPELIGSGVSVAKADPGIIQR------SVAAVH---AVNPKVKVLTGAGIQSGECVKIARDLGTDGVLLASSVVKVEDPAI-
Thermoproteus_tenax             --------------VAVEPPELIGTGRAVSRYKPEAIVE------TVGLVS---RHFPEVSVITGAGIESGDDVAAALRLGTRGVLLASAAVKAKDPYA-
Pyrobaculum_islandicum          --------------VAVEPPELIGTGKAVSKYKPETIIE------TVRLVT---KHFPNVVVITGAGIETGEDVEAALKLGTKGVLLASAAVKAKDHYQ-
Cenarchaeum_symbiosum           --------------IAIEPPELIGSGRSVSSERPELIGE------AAEAIR---GADG-TKLLCGAGITSGADVRKALELGSKGILVASGVVKSSDPAA-
Nitrosopumilus_maritimus        --------------IAIEPPELIGSGKAVSTERPELITK------AANAVK---SANNNTKLLCGAGIVSGQDVSKAVELGSKGILVASGIIKAKNWDK-
Desulfurococcus_kamchatkensis   --------------VAVEPPELIGTGVSVSKAKPEVVTN------SVDMIR---RVNKDVAILTGAGITTGEDAYIAVKLGTIGVLVASGIVKAKDPYQ-
Pyrolobus_fumarii               --------------VAVEPPELIGTGIAVSRAKPEVVTK------AVESVT---RVSPNVPVLVGAGIVSREDARRSVELGARGVLVASAVMKAADPYA-
Acidilobus_saccharovorans       --------------IAIEPPELIGTGVSVSKARPEVITE------GVKAVK---AVA-DIPVLAGAGITYREDVVRAVQLGASGILLASAVMKAADPDK-
Sulfolobus_islandicus           --------------ILIEPPELIGTGISVSKAKPEVITK------AVDEIR---KSE-GIYLIAGAGITTGEDVYKALKLGAHGIGVASAVMKAKEPEK-
Clustal Consensus                             .  **   :*                                     :  *..                *  :. .
```

```
                                             310       320
                                       ....|....|....|....|
Roseiflexus_castenholzii               --IVHFLSPQE---------
Chloroflexus_aurantiacus               --IIHYQ-------------
Nostoc_punctiforme                     --IVNFHLV-----------
Anabaena_variabilis                    --IVNYQ-------------
Synechocystis_sp                       --IVNFQP------------
Cyanobacterium_aponinum                --LVNYQ-------------
Crocosphaera_watsonii                  --IINYQ-------------
Trichodesmium_erythraeum               --LVNYQ-------------
Synechococcus_sp                       --LANYQDQ-----------
Prochlorococcus_marinus                --ISNYKVE-----------
Sphaerobacter_thermophilus             --IVERAAALRL--------
Anaerolinea_thermophila                --IVQAAAEAKA--------
Myxococcus_xanthus                     --IVKAAT------------
Collinsella_tanakaei                   --MVVKSAE-----------
Atopobium_vaginae                      --MVKAAM------------
Eggerthella_sp                         --LVKACL------------
Dictyoglomus_thermophilum              --IVKS--------------
Aquifex_aeolicus                       --IVYSF-------------
Thermodesulfovibrio_yellowston         --IVKY--------------
Dehalogenimonas_lykanthroporep         --IVRKS--VQS--------
Dehalococcoides_sp                     --ICRQAAVIQNKH------
Geobacter_metallireducens              --IVNYKA------------
Desulfobacter_postgatei                --IIRF--------------
Planctomyces_brasiliensis              --IIDAGVELSA--------
Spirochaeta_coccoides                  --IIEFGL------------
Denitrovibrio_acetiphilus              --LVNFNG------------
Prosthecochloris_aestuarii             --IVKAAEKS----------
Chlorobium_phaeobacteroides            --IVNAAG------------
Chlorobium_ferrooxidans                --IIKAAS------------
Chloroherpeton_thalassium              --IIKSAE------------
Treponema_pallidum                     --IARSV-------------
Leptospira_interrogans                 --LF----------------
Borrelia_turicatae                     --IVNKVAK-----------
Thermotoga_maritima                    --LARIMRGVIS--------
Fusobacterium_nucleatum                --IIKAGN------------
Selenomonas_sputigena                  --IVKF--------------
Ruminococcus_sp                        --IVNYK-------------
Clostridium_botulinum                  --IVNY--------------
Clostridium_perfringens                --IVNY--------------
Thermoanaerobacter_wiegelii            --IVNY--------------
Brachyspira_murdochii                  --IVNYIAK-----------
Acholeplasma_laidlawii                 --FTKAAKNK----------
Lactobacillus_reuteri                  --MVNYLKNDQEK-------
Bacillus_subtilis                      --LLEEGQYE----------
```

```
Bacillus_thuringiensis          --LLGAVK------------
Listeria_monocytogenes          --LLEAVK------------
Geobacillus_stearothermophilus  --LVEAGRHE----------
Staphylococcus_aureus           --LLEGAK------------
Enterococcus_faecalis           --LLDAVK------------
Lactococcus_lactis              --LLEMYK------------
Streptococcus_pneumoniae        --LLDFVK------------
Erysipelotrichaceae_bacterium   --IIEKTK------------
Lentisphaera_araneosa           --LILNAAALS---------
Chlamydophila_pneumoniae        --VAKNFNV-----------
Desulfovibrio_vulgaris          --IILA--------------
Mesorhizobium_opportunistum     --ILGRASAAI---------
Bartonella_henselae             --ICDVYRKL----------
Brucella_abortus                --ICETYRNL----------
Rhizobium_etli                  --IYRAYEALLA--------
Agrobacterium_tumefaciens       --IYAAYEQLTA--------
Rhodobacter_sphaeroides         --IVAALSAA----------
Ruegeria_sp                     --IIAALAAS----------
Bradyrhizobium_japonicum        --IAKGCP------------
Rhodopseudomonas_palustris      --IAAGCP------------
Methylobacterium_radiotolerans  --ICAAYA------------
Rhodospirillum_rubrum           --IAQSCA------------
Zymomonas_mobilis               --IIEAGGRLA---------
Gluconacetobacter_hansenii      --IVRAAVDI----------
Thermus_thermophilus            --LLRIAG------------
Thermus_aquaticus               --LLRMAG------------
Oceanithermus_profundus         --MVEAAG------------
Deinococcus_deserti             --MNDALK------------
Bifidobacterium_bifidum         --IARLTLKATRR-------
Bifidobacterium_longum          --IARLALKSTKSRN-----
Gardnerella_vaginalis           --IVQLSSYKNRI-------
Nocardia_farcinica              --LSAIAAGGPLP-------
Mycobacterium_tuberculosis      --LAAIAAGGPLP-------
Corynebacterium_glutamicum      --LAANAASVA---------
Propionibacterium_acnes         --IVTFYEA-----------
Actinomyces_odontolyticus       --MATFYA------------
Microbacterium_testaceum        --IIRYQKHVGV--------
Moritella_marina                --IAKAAAEAKA--------
Photobacterium_profundum        --IAKAAAEAKA--------
Vibrio_cholerae                 --IAKAAAEAKKA-------
Shigella_flexneri               --IVKAAEAAKQA-------
Escherichia_coli                --IVKAAEAAKQA-------
Salmonella_enterica             --IVKAAEAAKQA-------
Klebsiella_pneumoniae           --IVKAAEAAKKA-------
Enterobacter_aerogenes          --IVKAAEAAKKA-------
Serratia_symbiotica             --IVKAAGAAKKA-------
```

```
Yersinia_pestis                    --IVKAAAKAKKA-------
Actinobacillus_pleuropneumonia     --IVKAAEKAKA--------
Pseudomonas_aeruginosa             --ICRAAGS-----------
Marinomonas_posidonica             --IVKAAG------------
Xylella_fastidiosa                 --IARATV------------
Xanthomonas_campestris             --IARAAAAC----------
Marinobacter_algicola              --ICRAVPGKS---------
Aeromonas_caviae                   --IVEAAKGAS---------
Pseudoalteromonas_haloplanktis     --ICNSAKGTV---------
Legionella_pneumophila             --IVKCIN------------
Ralstonia_solanacearum             --IGRA--------------
Burkholderia_sp                    --ICKAAAATTVAD------
Bordetella_pertussis               --IAAA--------------
Nitrosococcus_watsonii             --ICQAAV------------
Coxiella_burnetii                  --IGQQCNQSF---------
Variovorax_paradoxus               --IISATAR-----------
Halomonas_elongata                 --ICQSAG------------
Tenebrio_Molitor                   --IINARQL-----------
Bombyx_mori                        --IVNANQ------------
Apis_mellifera                     --IVNAKQ------------
Aedes_aegypti                      --IVNARQ------------
Anopheles_gambiae                  --IVNARQ------------
Drosophila_melanogaster            --IINARQ------------
Rhipicephalus_microplus            --IINAMQG-----------
Ixodes_scapularis                  --IINARQ------------
Anolis_carolinensis                --IINAKH------------
Gallus_gallus                      --IINAKH------------
Orcinus_orca                       --IINAK-------------
Bos_taurus                         --IINAKQ------------
Canis_lupusfamiliaris              --IINAKQ------------
Odobenus_rosmarus                  --IINAKQ------------
Oryctolagus_cuniculus              --IINAKQ------------
Nomascus_leucogenys                --IINAKQ------------
Pan_troglodytes                    --IINAKQ------------
Homo_sapiens                       --IINAKQ------------
Pongo_abelii                       --IINAKQ------------
Macaca_mulatta                     --IINAKQ------------
Ochotona_princeps                  --IINAKQ------------
Sus_scrofa                         --IINAK-------------
Mus_musculus                       --IINAKQ------------
Rattus_norvegicus                  --IINAKQ------------
Xenopus_laevis                     --IINAKH------------
Xenopus_tropicalis                 --IINAKQ------------
Danio_rerio                        --IINAKA------------
Ictalurus_punctatus                --IINAKV------------
Oreochromis_niloticus              --IINAKA------------
```

```
Nematostella_vectensis              --IVNARL------------
Schistosoma_mansoni                 --ICKARQR-----------
Strongylocentrotus_purpuratus       --IIAAGSA-----------
Caenorhabditis_elegans              --IINARS------------
Caenorhabditis_remanei              --IINARS------------
Brugia_malayi                       --IIHARD------------
Trichinella_spiralis                --IIEARSN-----------
Ricinus_communis                    --IIKSAEVKKSV-------
Vitis_vinifera                      --IIKSAEVKKNC-------
Arabidopsis_thaliana                --IIKAAEVKKSA-------
Zea_mays                            --IINAATVKSA--------
Oryza_sativa                        --IINSATVKSA--------
Fragaria_vesca                      --IINAAVVKK---------
Glycine_max                         --IINAATVKKN--------
Cucumis_sativus                     --KFASFVFVHLFGC-----
Perkinsus_marinus                   --IIAAAAQSA---------
Aspergillus_niger                   --IINARL------------
Penicillium_chrysogenum             --IINARI------------
Paracoccidioides_brasiliensis       --IINARL------------
Schizosaccharomyces_pombe           --IVNVHSL-----------
Saccharomyces_cerevisiae            --IINSRN------------
Kluyveromyces_lactis                --IINSRV------------
Candida_dubliniensis                --IIKSRL------------
Clavispora_lusitaniae               --IIKSRL------------
Chlamydomonas_reinhardtii           --ICNAAGPKAKP-------
Volvox_carteri                      --ICNSAGPKAK--------
Dictyostelium_fasciculatum          --IINTANPKSYL-------
Entamoeba_histolytica               --IINSVSEKF---------
Cryptosporidium_parvum              -KIIESAQ------------
Cryptosporidium_muris               -EIINCTFH-----------
Plasmodium_falciparum               -DIIKSAM------------
Plasmodium_vivax                    -EIIKSAM------------
Paramecium_tetraurelia              -DIVAAANDAR---------
Tetrahymena_thermophila             -DIVAACNIVKH--------
Toxoplasma_gondii                   -DIIASGMKKNE--------
Trypanosoma_brucei                  -DIIKATQ------------
Trypanosoma_cruzi                   -EIIEATK------------
Leishmania_mexicana                 -DIIDATR------------
Leishmania_donovani                 -DIIDATR------------
Giardia_lamblia                     IDILTKTRT-----------
Trichomonas_vaginalis               --IVNSNVHSK---------
Sphingobacterium_sp                 --IVKVFN------------
Pedobacter_saltans                  --IVKTFNN-----------
Marivirga_tractuosa                 --IIKSF-------------
Cyclobacterium_marinum              --IAESF-------------
Leadbetterella_byssophila           --ISKSFPA-----------
```

```
Haliscomenobacter_hydrossis       --IVEATKK-----------
Parabacteroides_distasonis        --IIEAF-------------
Bacteroides_fragilis              --IIDAFN------------
Prevotella_ruminicola             --IIDAWKK-----------
Alistipes_shahii                  --IGKGFSK-----------
Porphyromonas_asaccharolytica     --IITANK------------
Cellulophaga_lytica               --IIDAI-------------
Kordia_algicida                   --IINAI-------------
Flavobacterium_psychrophilum      --IVNGI-------------
Neisseria_gonorrhoeae             --IISAAQNA----------
Francisella_tularensis            --IINQANKICTE-------
Mycoplasma_genitalium             --MARVYA------------
Ureaplasma_urealyticum            --IINACK------------
Ureaplasma_parvum                 --IIDKCK------------
Buchnera_aphidicola               --IIKIAHEHYS--------
Campylobacter_jejuni              --LIKG--------------
Helicobacter_pylori               --IISFL-------------
Orientia_tsutsugamushi            --IIKTVQGLD---------
Wolbachia_sp                      --IIQQVEKKFSLINSKISN
Methanocaldococcus_Jannaschii     -AIRELIKFI----------
Methanococcus_voltae              -SIRDLISEL----------
Methanothermococcus_okinawensi    -SIQELIKHI----------
Pyrococcus_Woesei                 -AIWDLVSGI----------
Pyrococcus_furiosus               -AIWDLVSGIIKE-------
Thermofilum_pendens               -AILDLISPIIKK-------
Methanothermobacter_thermautot    -ALLDLVSKV----------
Methanosaeta_thermophila          -ALEDLVTGV----------
Natronobacterium_gregoryi         -ALDDLVEPL----------
Halobacterium_salinarum           -ALADLVAPL----------
Halogeometricum_borinquense       -ALEDLVEPL----------
Methanosphaerula_palustris        -VLRDLVSLL----------
Thermoproteus_tenax               -KIVELAKPLSELR------
Pyrobaculum_islandicum            -KIFELAKPLTVAAEPP---
Cenarchaeum_symbiosum             -AIAELAQAMS---------
Nitrosopumilus_maritimus          -IISEFAKALV---------
Desulfurococcus_kamchatkensis     -VMKEMAEAMLKGLA-----
Pyrolobus_fumarii                 -KMRELAEGLAPRR------
Acidilobus_saccharovorans         -ALSEFVDALSSVA------
Sulfolobus_islandicus             -VVEDFITSALKAISS----
Clustal Consensus
```
